# Climate change impacts and mental health in poor urban coastal communities in Ghana

**DOI:** 10.1371/journal.pmen.0000284

**Published:** 2025-04-08

**Authors:** Sylvia Hagan, Ernest Darkwah, Yaw Agyeman Boafo, Collins Badu Agyemang, George Ekem-Ferguson

**Affiliations:** 1 Department of Psychology, University of Ghana, Legon, Ghana; 2 Centre for Climate Change and Sustainability Studies, University of Ghana, Legon, Ghana; 3 Department of Psychiatry, Korle Bu Teaching Hospital, Accra, Ghana; Sigmund Freud University Vienna, AUSTRIA

## Abstract

Coastal communities in African countries with lower carbon emissions face greater climate challenges but lack the capacity to address these challenges. The implication is that these communities suffer more from the impacts that climate change brings. Despite sustained research efforts into climate impacts on such communities, the mental health aspects of these impacts are often overlooked. In this study, we explored the lived experiences of climate-related mental health challenges and community coping mechanisms within three poor urban coastal communities in Ghana, West Africa. Fifty-seven community members participated in the study. Data were collected through five focus group discussions and fifteen one-on-one in-depth interviews. Thematic Network Analysis was used to analyse the data. Results showed that rising sea levels have caused loss of livelihoods and properties, which in turn have exacerbated mental health challenges within the communities. Community members’ coping mechanisms include the use of techno-managerial interventions, relocation, spirituality, and social support. The findings contribute to the broader discourse on climate change and its multifaceted consequences, highlighting the interconnectedness of environmental and mental health challenges in coastal landscapes.

## 1. Introduction

Climate change is a global environmental concern that has increasingly received attention within the last decade [1]. Rising temperatures, heat waves, floods, tornadoes, hurricanes, droughts, fires, and glaciers, along with the disappearance of rivers and desertification have all been recorded around the world in increasing proportions [2–4]. Globally, approximately 3.3 to 3.6 billion individuals reside in areas that are highly vulnerable to climate change impacts [1]. Between 2010 and 2020, human deaths due to floods, droughts, and storms were 15 times greater in highly vulnerable regions than those in low vulnerability areas [1].

Climate change impacts often result in climate injustices where those who contribute least to greenhouse gas (GHG) emissions such as people in poor coastal communities in low- and middle-income countries end up becoming the most affected [5]. The consequences of such injustices and vulnerabilities may include climate-induced mental health challenges, which may be comorbid with other, more physically apparent consequences often observed among these populations [6]. Therefore, there is an urgent need to gain a deeper understanding of the climate-induced mental health challenges [7,8] in poor urban coastal communities in Ghana.

Within the framework of the definition of vulnerability provided by the Intergovernmental Panel on Climate Change (IPCC), [9], it can be understood that a community’s sensitivity and ability to adapt to climate change are influenced by factors such as socioeconomic characteristics, location, perceptions of risks and sea level rise [10]. In this light, poor urban coastal communities in Ghana can be described as vulnerable due to their high exposure to climate stressors, persistent socio-economic and infrastructural deficiencies, and low adaptive capacity [9]. The vulnerabilities of these communities particularly to incidents such as flooding are exacerbated by rising sea levels and intense rainfall [11–13]. For instance, Dansoman (Panbros, Glefe, and Gbegbeyise) communities have high sensitivity and low adaptive capacity because they experience high-level sea rise which often leads to extensive infrastructural damage and displacement of residents [14]. Many residents also reside in substandard housing that cannot withstand severe weather conditions [12,15]. Informal settlements in these areas often lack adequate drainage systems, making them especially prone to flooding coupled with poor sanitation, poverty, and insufficient infrastructure which further increase health risks [12].

Over the years, researchers in Ghana have consistently explored climate change-related issues in coastal communities. Many of these studies have focused on climate-induced stressors facing poor urban communities while others have outlined significant climate change risks associated with coastal living [11,14,16]. Others still have extensively examined community vulnerabilities, highlighting risk factors, causes of flooding, and emergency responses in informal settlements [10,12,13,17,18]. However, climate change-induced mental health experiences of people in poor urban communities in Ghana seem to either be conspicuously missing in these studies or be primarily centered on common issues such as depression and perceived powerlessness, along with their community-based predictors [19–21]. While the evidence produced from these studies is valuable and has contributed to existing climate response strategies, the lack of insight into lived experiences of the mental health outcomes of climate change incidents in the Ghanaian context bodes ill for policy and intervention. Therefore, this study augments the existing literature by exploring the lived experiences of mental health issues associated with climate change impacts, particularly sea-level rise, and the coping mechanisms individuals employ in three poor urban coastal communities in Accra, Ghana. The idea is to provide additional context-based insights into the mental health impacts of climate change to support efforts towards Sustainable Development Goal (SDG) 13 (Climate action) and SDG 3 (Good Health and Well-being) in Ghana.

## 2. Method

### 2.1. Research setting and target population

The study was conducted in three poor urban coastal communities in Ghana, West Africa. The communities were Glefe, Gbegbeyise, and Shiabu located within the Ablekuma-West Municipality of the Greater Accra region, which also hosts the capital city, Accra. The municipality’s coastline hosts a significant number of informal urban settlers, including squatters and densely populated slums [12].

Glefe is located between two lagoons, salt mining ponds, and the ocean, making it highly susceptible to flooding [22]. This community faces annual inundation, primarily triggered by heavy rainfall. Such frequent and severe flooding from both natural (heavy rainfall and sea level rise) and human-made (dam spillage) events expose Glefe to climate risks. The economic repercussions of these floods are substantial, exacerbating poverty among residents and increasing their vulnerability to climate risk. Efforts to mitigate these impacts have progressed with the construction of the Dansoman Emergency Sea Defence.

The Dansoman Emergency Sea Defence is one of the reactive coastal management systems in Ghana [23]. This project is part of the government’s efforts to combat coastal erosion, inundation and gradual submersion of land resulting from high spring tides (sea level rise) and extreme weather patterns that affect personal properties and people’s livelihoods [24]. The revetment stretches from the Panbros area to the beach road. The construction materials include igneous rocks and beach sand transported to the site [24]. At the time of this study, the first phase of this sea defence had been completed, aiming to reduce the impact of sea-related flooding, and indicating some level of adaptive capacity.

Gbegbeyise’s exposure to climate risks is substantial due to its location along the shoreline. The community experiences recurrent flooding caused by episodic spring tides and high tides [12,14]. This frequent exposure to flooding events places the community at a high risk of climate-related impacts. At the time of data collection, the second phase of the Dansoman Emergency Sea Defencehad not yet been constructed. This incomplete infrastructure project highlights the community’s limited adaptive capacity.

The Shiabu community experiences increased vulnerability to heavy rainfall due to inadequate solid waste management and poorly designed drainage systems [15]. These shortcomings lead to frequent flooding events. Shiabu’s geographical layout and unregulated sand mining along its coastline contribute to its vulnerability to rising sea levels. Coastal erosion and inundation are significant concerns, threatening homes, livelihoods, and infrastructure. At the time of this study, the second phase of the Dansoman Emergency Sea Defence had not been constructed, indicating limited adaptive capacity. The absence of adequate coastal protection measures leaves Shiabu vulnerable to ongoing and future climate impacts.

### 2.2. Participants

All participants were selected through purposive and convenient methods. Participants were selected based on specific characteristics that aligned with the study objectives. Criteria for participant selection include geographical location, exposure to climate-related stressors, and willingness to participate in the study. Community stakeholders’ selection was based on their lived experiences with climate-related issues and their availability to participate in the study. By targeting participants with direct and relevant experiences, the study was able to generate meaningful insights related to climate change impacts and mental health challenges.

Initially, sixty – six community members were approached face-to-face to participate in the study. Nine community members declined to participate in the research due to time constraints. Eventually, fifty-seven community members volunteered to participate and were involved in focus group discussions (five groups involving forty- two participants) and one-on-one in-depth interviews (involving fifteen participants). S1 Table presents the demographic characteristics of the participants.

All participants were invited to take part in both focus group discussions and individual interviews. While some opted to participate in both, others chose either the focus groups or the individual interviews based on their preference. The focus group discussions allowed participants to express subjective as well as shared lived experiences and meaning-making of the communal situation. Focus group discussions were chosen for their ability to stimulate ideas and issues among participants that might otherwise be overlooked during participant observations or individual interviews [25]. The Ghanaian culture often emphasises communal living, collective decision-making, and shared experiences [26]. Hence using focus group discussions helped the researchers tap into collective knowledge and norms in the communities. Additionally, they allowed the researchers to identify individuals who appeared to have more to share, enabling us to select them for further interviews. The discussions also provided valuable insights into shared experiences and norms within the community of the participants.

Data collection concluded with in-depth, face-to-face interviews conducted with participants identified through participant observations and focus group discussions. The one-on-one interviews allowed in-depth conversations that captured personal experiences and perspectives. The interviews were conducted at times and locations convenient for each participant either in their homes or workplace. Scheduling these interviews at times and locations convenient for participants allowed us to gather additional information, enriching the dataset and enhancing its reliability and credibility [27]. The three data sources (participant observations, focus group discussions, and interviews) formed a robust foundation for triangulation [28]. This approach also provided an opportunity to delve into topics that some participants were hesitant to address during the focus group discussions.

Additionally, the participants included community stakeholders such as administrative staff in the community hospital, the sea defence project manager, assemblymen and unit committee members to get deeper insights. By combining these methods, the authors were able to enrich their findings through multiple lenses. This approach also helps to ensure the integrity and validity of the research outcomes by cross-checking information gathered from different sources [29].

### 2.3. Material and procedure

A semi-structured interview guide was crafted based on key themes identified in prior climate change and mental health studies [30,31]. Sample questions in the interview guide include:. How have you experienced climate-related incidents in the past years? How does climate change impacts (sea level rise) currently affect your life, family, or business? How do you cope with the challenges caused by the sea level rise? How do you feel when you think about climate change impacts (sea level rise)? Tell me how you manage your feelings. These research questions were piloted with five community members before the main interviews were conducted. The recruitment period and data collection took about six months from 15th February 2024 and ending on 20^th^ July 2024.

Participants provided informed consent, which was obtained orally. The verbal consent process was witnessed, and audio recorded by the authors and research assistants. The duration of the one-on-one in-depth interviews typically ranged from 30 minutes to 45 minutes, although two interviews lasted only 6 minutes due to time constraints on the informant’s part. The focus group discussion lasted for 20 minutes - 40 minutes. The interviews were conducted in either English, Twi or Ga (local Ghanaian language) depending on the preference of the participant.

We reached data saturation after interviewing fifteen individuals and five focus groups when novel information was absent. Data repetition occurred reinforcing rather than enriching previously extracted information from participants and additional sampling did not yield new insights [32]. Typically, 10 to 50 participants suffice to achieve data saturation in phenomenological studies [33].

All interviews were recorded and transcribed verbatim. Two research assistants worked with the lead author and fifth author to carry out the transcription and coding. The research assistants were bachelor’s degree holders trained in conducting qualitative research methods in the social sciences. The study adhered to COREQ guidelines, a standardised framework for reporting qualitative research, to ensure credibility and quality in reporting qualitative studies [34] S1Checklist.

The interviews were conducted by the lead author, the second author, and the fifth author. The second author holds a Ph.D. while the lead author and the fifth author are Ph.D. candidates. All the interviewers are involved in mental health research and had training in qualitative research methodology. Neither author lived in the community, allowing participants to share their experiences freely.

Furthermore, the authors’ positionality as Ghanaian researchers precisely from the Akan ethnic group, gender, socioeconomic status, culture, and educational level may have influenced their understanding of the community members’ lived experiences. To address this, the authors were mindful of potential biases that might arise from personal and professional backgrounds [35]. The authors took time to think deeply about their perspectives, making sure they were fair and unbiased.

Before the study began, the lead researcher established rapport with participants, explaining the study’s purpose and addressing any concerns, which helped create a trusting and open environment. Participants were informed about the lead researchers’ background and motivations, particularly the interest in mental health challenges faced by coastal residents due to climate change in Ghana.

### 2.4. Ethics

Ethical clearance was granted by the Departmental Research Ethics Committee of the Department of Psychology at the University of Ghana (DREC) (DREC/026/23-24). Additionally, permission was obtained from community gatekeepers. Informed consent was obtained from all participants orally.

### 2.5. Analysis

Thematic Network Analysis was used to analyse the transcribed data [36] S1 Data. Initially, the transcripts were thoroughly reviewed multiple times to gain familiarity with the data [37]. Additionally, audio recordings were frequently revisited alongside transcript readings and field notes. This active engagement provided insight into how participants experienced life and mental health within their communities.

Next, we developed codes and themes from the data using the Attride-Stirling thematic networks approach which identifies three kinds of themes in thematic analysis: Basic Themes, Organising Themes, and Global Themes [36]. First, we clustered codes that seemed to convey similar meanings into Basic Themes and Basic Themes that centered around similar experiences into organising themes. We used the same approach to cluster organising themes into an umbrella global theme. Throughout this process, we paid attention to our backgrounds and potential biases that could have impacted our interactions with participants and the interpretation of our findings. To minimise these influences, we employed reflexive practices throughout the research process. This included constant self-reflection on our positionalities and discussions among all authors to identify and address any emerging biases [29,35,38] This approach aimed to ensure that participants voices and experiences were represented accurately and respectfully.

Furthermore, we employed several strategies consistent with Creswell and Miller, Steinke, and Whittemore recommendations to ensure transferability, dependability, and confirmability [39–41]. We adopted thick descriptions of context to address transferability by providing participant demographics and context. We used an audit trail to address dependability through a transparent and systematic approach to data collection and analysis, documenting each step of the research process, from the initial coding to the development of themes. We triangulated data from focus groups to address confirmability in validating the findings and minimising personal biases. S2 Table presents the thematic data analyses process and the themes that were developed.

## 3. Results

The results of the data analyses revealed that climate change events, particularly sea level rise, continue to negatively impact the lives of coastal communities in Ghana. The mental health impacts of climate change on these communities were found to be profound and multifaceted, reflecting the broader truth that environmental stressors exacerbate existing socio-economic and psychological vulnerabilities. For clarity and simplicity, we present the results in accordance with the organising themes and basic themes that were developed from the data.

### 3.1. Experiences of climate change impacts (sea level rise)

Although the individual experiences shared by participants in regard to the impacts that rising sea levels have on their lives were wide and varied, one basic theme that seemed to run through all the experiences was that of shock and disbelief about the drastic changes in sea proximity over what the community perceived to be a very short time.

*“The water was very far from our homes. Ooh! Very far. I gave birth to all my children in this house and the sea was not close to us like this” (Participant 12).*

Another participant stated that:

*“I grew up in this community, the sea was not close to us. But now, it is very close to our homes. During the rainy season, it comes to our homes.” (Participant 4)*

Participants’ experiences of the impact of this rapid sea level rise seemed to be that of profound loss of livelihoods and property. Many participants recounted losing their homes and businesses and finding themselves having to adjust by looking for almost non-existent alternative livelihoods. Business owners reported the destruction of their enterprises, which left them without financial safety nets or means to rebuild. One participant said:

*“I used to have a thriving business here, but everything was washed away. I was the largest supplier of soft drinks and alcoholic beverages in this whole area…just ask about me…(tears)… I have lost my business, my investment, years of hard work and dedication that went into building it…… (Participant 9)*

Another participant shared this experience.

*“The sea is destroying our buildings. All houses on this lane have been destroyed by the sea. Nobody can stop the sea from coming and we can’t do anything about it” (Participant 15).*

### 3.2. Climate-related mental health experiences and challenges

The profound loss and shock that the community experienced as a result of the rising sea levels seemed to generate mental health consequences for the community members. Participant accounts highlighted basic themes of overwhelming sense of pain and despair, learned helplessness and hopelessness as well as feelings of anxiety.

The experience of pain and despair was almost unanimous as every participant had such an experience to share. One participant said:

*“…go and ask about my brother over there (pointing to a man seated on a chair in a ruined building),… he used to have a big bar over there... Now everyone knows his loss has taken over his head, his heart is in pain, he has gone mental, he is gone, my brother is gone, he needs the Psychiatric hospital…” (Participant 1)*

Another participant shared this experience

*“The sea has destroyed everything, the building collapsed on me, and I almost lost my legs too” (Participant 14)*

The feeling of pain and despair seemed to be exacerbated by the financial strain caused by the loss of property and livelihoods, as most participants struggled to see a way forward or find meaning in their circumstances. The narrative below is illustrative of this:

*“…I am a fishmonger, and my husband is a fisherman. We used to have* a *thriving fish business here, but the sea has washed away nine of my rooms and my fish business has also collapsed. I have little money…”(Participant 7)*

Another participant also stated that:

*I have used the little money I have left after the sea destroyed my pub to build this small pub. I have lost everything. My life is very empty, and I regret not traveling abroad in 2007 when I was a footballer. Now, I am just here with nothing, nothing. (Participant 14)*

Many of the participants also felt like no matter what they did, they couldn’t stop the sea from getting to their homes. This feeling of helplessness can be overwhelming because it seems like the problem is too big for them to solve on their own. The findings suggest frustration and helplessness due to the lack of governmental and media action to address tidal waves. The narrative below is illustrative.

*“I have put sand in sacks, but the sea just destroys it. I feel like no matter what we do, the sea just keeps coming closer. We cannot do anything about the sea. It is just coming”* (Participant 12)

Another participant also stated that

*“We wrote to the municipal assembly to help us with our situation. But nobody has been here. I voted for this government, but no one is helping us with the sea issue. People come and take pictures here and they go but nothing is done. And we are still here” (Participant 5)*

Regarding the feeling of anxiety, participants experienced persistent feelings of apprehension and a sense of impending doom from the loss of natural landscapes, and the realisation of the irreversible changes occurring in the environment. The constant worry about future storms and flooding disrupts sleep, leading to anxiety. Witnessing the destruction of one’s property and the broader impacts of climate change evokes a profound sense of anxiety. This form of anxiety is often referred to as eco-anxiety [42]. The narrative below is illustrative.

*“As I am going to sleep, I am afraid that I will wake up tomorrow and the sea has taken my shop. I am always thinking. When I sleep, I can’t sleep. Thinking and thinking (Participant 20)*

Another participant also stated that

*“I haven’t slept for about a week because I’m scared that the last room might collapse on my children while they sleep…(Participant 6).*

There is also fear of expanding businesses because of uncertainty about the future.

*“I am afraid to expand my business because I don’t know if the sea will destroy my business tomorrow. So, we are just here. just staying here, no one knows tomorrow” (Participant 10).*

Another participant also stated that

*My mother (owner of the shop) has refused to buy new goods from the shop because she doesn’t know when the sea can destroy the shop (Participant 3).*The findings reflect a deep sense of uncertainty and fear among small business owners about the future due to the threat of sea-level rise. This anxiety prevents them from expanding or investing in their businesses, as they worry about potential destruction from the sea at any time.

### 3.3. Coping mechanisms

Despite the overwhelming challenges, participants identified various coping resources that helped them navigate climate-related mental health challenges, that is psychological and physical coping mechanisms.

Psychological coping mechanisms are strategies individuals use to manage their mental health challenges associated with rising sea levels. These coping strategies included social support, relocation and spirituality. The narrative below is illustrative of social support.

*“I know the people whose houses have been destroyed by the sea moved to live with their family members in Accra. They are no longer here because their family members supported them with accommodation” (Participant 2).*

Another participant also stated that

*“Some people come here to encourage me from time to time. That makes me feel better and strong” (Participant 7).*

Sharing experiences and seeking advice from others in similar situations can reduce feelings of fear and anxiety. Family support helps to manage their experiences and maintain their psychological health.

Also, hope for relocation represents a tangible adaptive strategy for a safer future in the face of climate change impacts. The narrative below is illustrative.

*“My room used to be there too, but I have rented now, I just work here, if the sea persists in destroying my shop, I will go back to my hometown” (Participant 16).*

Another participant added:

*“My mother has another shop. If the sea destroys this one. I will move there and operate our business there”. (Participant 3).*

Another participant also added:

*“My body is here but my soul is not here. I am saving money to leave this community. I am still here because I don’t have money to leave” (Participant 13).*

Moreover, spirituality offers psychological support, helping participants better cope with the mental health challenges posed by the rising sea levels. Stress Relief practices like prayer provide comfort and reduce stress. Spirituality helps participants accept that the problem is beyond their control and belief in a higher power can alleviate anxiety and fear about the future, providing a sense of security. The narrative below is illustrative.

*“When I wake up, I stand close to the sea and pray to the sea to give me some time to stay here till I get money to leave. Do you know the sea can hear us? It listens to us.”(Participant 17).*

Another participant added:

*Nobody can stop the sea from destroying our homes, but we pray that it doesn’t. God is the only one helping us now (Participant 13)*

In the case of techno-managerial interventions, particularly sea defence is a physical coping mechanism the government put in place to adapt to rising sea levels in Ghana. All participants accepted their limitations in addressing environmental issues locally. In Shiabu, the issue of the stalled construction of the second phase of the Dansoman Emergency Sea defence has evoked mixed perspectives among its residents. Some participants seem to find solace in the hope that the government will complete the second phase of the sea defence project. The experiences of the participants seem to underscore broader issues of governance, resource allocation, and community resilience in the face of environmental challenges. The narrative below is illustrative.

*“All the worry is about the sea defence project. They are not continuing. Those are all our problems. If they continue the defence, everything will be ok” (Participant 19).*

Another participant also stated

*“So, we are pleading with the government to finish building the sea defence project early so that we can also work here in peace. The way we are working, and the sea is destroying the work, it is causing us to waste money. Yeah, so we are pleading with them to do the sea defence, they should do the defence for us early” (Participant 10).*

Conversely, some residents express deep displeasure and a sense of injustice towards the government. This highlights the importance of effective communication, transparency in decision-making, and equitable distribution of resources to ensure that all communities, especially those vulnerable to sea level rise, receive the necessary infrastructure and protection. The narrative below is illustrative.

*“The previous government built the sea defence for the people of Glefe, but this new government did not continue the project. I voted for this current government, but they have not been fair to us at all. I will not vote for this government again because they have lied to us (Participant 17).*

Another participant also stated

*“The current Member of Parliament in the area has not helped us at all. We the people of Shiabu are not all happy at all about the discontinuation of the sea defence because it is destroying our homes” (Participant 10).*

Another way participants adapt to the sea level rise is the use of rocks and sand in sacks. While many participants hope for the construction of the sea defence which they believe is a more sustained solution, most participants use their money to buy rocks or put sand in sacks to protect their businesses and houses even though they know it’s not sustainable. The narrative below is illustrative.

“…*That’s why I was forced to spend money to do it (buy rocks) even though the government has not been able to continue the sea defence …(Participant 5).*

Another participant also stated

*“We use the sacks and put sand in them to protect our houses. If not the sacks, the sea would have destroyed the building for a long time. But when the sea gets angry it will break the house, simple! Nothing can stop the sea from destroying the houses if it wants to”. (Participant 7)*

## 4. Discussion

Climate change impacts intersect with other socio-environmental processes to shape local development settings and vulnerability contexts. Coastal areas frequently suffer from rising sea levels [43]. In poor urban coastal communities, climate change is seldom a single stressor. Multiple processes, including urbanisation, socio-economic disparities, and environmental degradation, compound the effects of climate change. The mental health challenges experienced by community members are intertwined with these broader socio-environmental dynamics.

In this study, we explored individual and shared living experiences of climate change impacts and their mental health implications in some coastal communities in Ghana. We found that rising sea levels due to climate change have caused a loss of livelihoods and properties which exacerbates individual mental health challenges. This finding is consistent with other studies conducted in other countries which indicate that climate change exacerbates mental health challenges [44–47]. This study highlights the need for a holistic approach to address climate injustice, integrating both physical and psychological well-being into adaptation strategies.

Findings suggested that hope in the construction of techno-managerial interventions, such as sea defences, plays a significant role in climate adaptation strategies. However, physical infrastructure alone cannot eliminate all risks. Grey infrastructure does not address the root causes of erosion [48]. These structures lack dynamic protection and fail to offer ecological benefits [49]. They contribute to coastal squeeze by obstructing the natural landward shift of the shoreline [50]. Additionally, they can cause sediment depletion and shift erosion to adjacent areas, creating new erosion hotspots [51]. Without regular maintenance, grey infrastructure becomes susceptible to climate change, necessitating significant additional investment to maintain coastal defences in areas with high erosion rates [52]. Effective climate adaptation requires a holistic approach that integrates grey and green infrastructure coupled with strategies to address socioeconomic challenges. For instance, economic inequality can continue to exacerbate the impacts of climate change even with physical protection in place.

Moreover, reliance on physical infrastructure can lead to a false sense of security, potentially encouraging them to practice sand-winning and sea pollution. Sand winning or mining involves extracting sand from various sources, including open pits on agricultural or forest lands, beaches, inland dunes, and riverbeds, primarily for construction purposes [53,54]. This practice is widespread along coastlines in developing countries, including Ghana [55]. Despite being illegal and harmful to the environment, coastal sand mining persists because those involved often overlook regulations and environmental impacts when it becomes a profitable economic activity and a means of livelihood [55,56].

Community members may not realise that removing the sand weakens the natural barrier that complements the sea defence. Over time, as the sand is depleted, seawater can begin to penetrate through or around the rocks, eventually breaching the defences and flooding the community. Additionally, because these communities live close to the sea defences, they may see the sea as a convenient dumping site, leading to increased sea pollution. This practice further compromises the coastal ecosystem, making the community even more vulnerable to future environmental challenges. The development of complementary adaptive measures like planting trees along the sea, education and strict enforcement of environmental policies may reduce the risk of climate change impacts on Ghana’s coastline.

Community members experienced social support and spirituality as a coping resource, reflecting a critical adaptive strategy in the face of climate change impacts. This finding aligns with existing literature that emphasises the role of collectiveness and religiosity in coping with climate change stressors [57,58]. In some Ghanaian communities’ spiritual beliefs and practices are deeply embedded in daily life [59]. Spirituality often serves as a source of resilience, providing individuals with a sense of purpose, hope, and community during times of stress [60]. Many residents engage in spiritual practices, including prayer which not only offers solace but also fosters a strong sense of community. These practices help mitigate feelings of anxiety and helplessness, providing a framework for understanding and enduring the hardships caused by climate change impacts. Additionally, social support has been examined as a critical buffer against the adverse effects of stress and trauma particularly in Ghana [61–63].

Furthermore, this study finding suggests that the hope to relocate is one of the coping strategies. For many in poor urban coastal communities, the possibility of relocation represents a tangible hope for a safer and more stable future. This finding is in line with other studies that suggest that migration is an adaptive response to environmental changes [64,65]. These coping mechanisms help individuals maintain a sense of control, even when faced with significant challenges such as rising sea levels and loss of property.

## 5. Recommendations

Future research should focus on quantifying the burden of climate-related mental health issues, exploring gendered impacts, and evaluating the long-term effectiveness of coping mechanisms such as spirituality and relocation in Ghana.

Understanding the prevalence of climate-related stress and anxiety through quantification is essential for addressing mental health challenges [66]. This enables researchers to identify patterns of anxiety, depression, and trauma linked to climate-induced challenges. National-based and community-based data can inform interventions tailored to address the specific needs of affected populations.

Examining the gendered impacts of climate change is crucial, as men and women may experience and cope with these stressors differently due to social roles [67,68]. In many coastal communities, women are predominantly involved in fish mongering, a livelihood heavily dependent on a steady supply of fish in Ghana. Climate change, through rising sea temperatures, has led to declining fish stocks, directly impacting their incomes and economic security [69]. This creates additional burdens as women struggle to support their families, often with limited access to financial resources or alternative income opportunities. On the other hand, men, who are often viewed as family breadwinners, may experience pressure to maintain livelihoods in the face of climate-induced economic disruptions, leading to increased stress and migration for work. Addressing these gender-specific experiences is vital for designing effective climate adaptation and resilience strategies tailored to the unique needs of both genders.

Moreover, exploring the effectiveness of coping mechanisms, such as spirituality, which offers psychological resilience and relocation, which might provide physical safety, is essential to developing comprehensive adaptation strategies. These efforts can inform targeted interventions, support services, and policies that address the mental health needs of individuals facing the multifaceted impacts of climate change.

In terms of governance, reforms should promote intersectoral collaboration, bringing together environmental, health, and civil society stakeholders to address these challenges holistically. By fostering collaboration across different sectors, it becomes possible to create integrated and comprehensive strategies that encompass all aspects of climate adaptation and human well-being particularly mental health.

Public education campaigns are needed to encourage community members to seek psychological support. Establishing and providing community-based mental health services and culturally sensitive support tailored to the unique needs of these vulnerable populations is essential. Safe spaces for vulnerable groups, particularly youth and women, can provide opportunities for sharing experiences, accessing mental health resources, and building resilience. These services also allow for the decentralisation of mental health care, making it more accessible for community members. By focusing on these areas, future research and policies can create a supportive framework that addresseses the mental health challenges posed by climate change and builds long-term resilience and adaptability within these communities.

## 6. Conclusion

The study employed qualitative humanistic methods to explore the lived experiences of mental health issues associated with climate change impacts in three poor urban coastal communities in Accra, Ghana. The findings revealed climate change impacts particularly sea level rise continue to impact negatively on the lives of coastal communities in Ghana. Community members experienced loss of property and livelihoods due to sea-level rise. The losses in turn create mental health challenges such as despair, helplessness and hopelessness as well as anxiety. Despite these challenges, community members coping mechanisms include the use of techno-managerial interventions, relocation, spirituality and social support. The study highlighted the urgent need for effective climate change adaptation strategies that incorporate mental health remedies in climate-related community-based interventions.

In the coming years, if nothing is done to address these climate change challenges, the compounded stress from displacement, economic losses, and health risks will deepen poverty, exacerbate inequalities, and increase mental health crises. Rising sea levels will likely lead to permanent loss of land and displacement of entire neighborhoods. To mitigate these impacts, targeted solutions are essential. Investments in resilient housing, improved drainage systems, and hybrid (integrated green and grey) infrastructure at the coastal shoreline can reduce physical vulnerabilities. Sustainable livelihoods and access to community-level mental healthcare can reduce socioeconomic challenges. Community-based adaptation programs, such as climate education and psychoeducation should be encouraged to empower residents. Collaboration among community stakeholders, mental health professionals, policymakers, and researchers is crucial for developing and implementing effective strategies to support mental well-being in vulnerable communities.

## Supporting information

S1 Data Interviews. (PDF)

S1 Table Demographic characteristics of participants. (DOCX)

S2 Table Thematic analysis of collected data. (DOCX)

S1 Checklist COREQ (COnsolidated criteria for REporting Qualitative research) Checklist. (PDF)
