# Supplementary material for: Climate change impacts and mental health in poor urban coastal communities in Ghana
Source: PLOS Ment Health. 2025 Apr 8;2(4):e0000284. doi: 10.1371/journal.pmen.0000284 (PMC12798396; doi:10.1371/journal.pmen.0000284)
Supplement: S1 Data — (PDF) [file pmen.0000284.s001.pdf]

CLIMATE MENTAL HEALTH INITIATIVE

TRANSCRIPT

INDIVIDUAL INTERVIEW_1

Date:15th February, 2024

Transcription code

(…) – Incomplete sentence

(xxx) – Not audible

[overlapping talk begins

] overlapping talk ends

(..) pause

'Dressss' lengthening of a word

Becau – cut off, interruption of a sound

I DON'T – Loud sounds/words

(Left hand on the neck) body conduct

Interviewer: First author

Interview starts – Participant 1

Interviewer: Thank you for taking the time to speak with me today. Thank you for agreeing to record this session.

Respondent: Yes, no problem

Interviewer: Can you start by telling me a bit about yourself and what you do? How old are you?

Respondent: I turned 52 years old this year. I've been here for most of my life. I am a food vendor, selling cooked meals by the roadside. But things have changed a lot in the last few years in this community.

Interviewer: I'm sorry to hear that. Can you tell me a bit more about what happened to your community?

Respondent: The sea has destroyed many buildings, houses, and businesses. Some years ago, this community used to be very vibrant, but now everything is gone. All the buildings are gone. We at Shiabu are suffering, but the people in Glefe are fine. In 2016, the government started constructing a sea defence to prevent the water from destroying our homes. They began the project in Glefe, and I was very lucky, the sea defence ended right at my house. So, my building hasn't been affected. But when you go further ahead" (points to the left), "it's very sad. All the buildings there are gone... Go and ask about my brother over there (pointing to a man seated on a chair in a ruined building),… he used to have a big bar over there... Now everyone knows his loss has taken over his head, his heart is in pain, he has gone mental, he is gone, my brother is gone, he needs the Psychiatric hospital...

Interviewer: That sounds really tough.

Respondent: It's very hard. Some people try to buy stones to protect their homes, but it doesn't always work. The sea is too powerful. You put the stones there, you bring sand, and before you know it, the waves wash everything away. It's heartbreaking.

Interviewer: And I imagine buying the stones isn't cheap?

Respondent: No, not at all. The stones are very expensive. Only the people who have money, what I'd call the "seemingly rich" ones, can afford to buy them. For the rest, it's just not possible. Some people save up for months to buy a few stones, but it's never enough. And the sea doesn't care how much you've spent. It will still come and destroy everything.

Interviewer: What happens to those who can't afford the stones?

Respondent: Shakes head They lose everything. Their homes, their businesses,gone. Some have no choice but to leave and move to other areas. But even that costs money, and many don't have it. So, they stay and try to manage, even though the sea keeps coming closer.

Interviewer: Do you think the government should step in and provide more support?

Respondent: Absolutely! The government started the sea defence, and that gave us hope. But they stopped halfway, and now we're suffering. If they had continued the project, maybe things would be different. The people in Glefe are fine because the sea defence protects them. But what about the rest of us? The government says they're working on it, but I don't see any real changes. The rains keep coming, the floods keep coming, and no one is doing enough to stop it. I feel like we've been forgotten. Sometimes I wonder if they even know what it's like for people like us.

Interviewer: It seems like the sea defence made a big difference in Glefe. Do you think extending it to the rest of the community would help?

Respondent: Yes, it would. If they had extended it to cover all of Shiabu, we wouldn’t be in this mess. My house was lucky because the sea defence ended there, but when I look at the rest of the community, it breaks my heart. People have lost everything,homes, shops, everything. then speaks slowly I want to believe there is. But we need help. We can’t do this on our own. The government needs to come back and finish what it started. If they don’t, I’m afraid the whole community will be gone in a few years.

Interviewer: What keeps you going despite everything?

Respondent: Smiles faintly My faith. I pray every day for a better future. Hmm, I see. But, to be honest, we’ve seen many people like you come here, asking questions and writing things down, but nothing has changed. We are still here, and the sea is still destroying everything.

Interviewer: I completely understand why you feel that way. It must be frustrating to keep sharing your story and not see results.

Respondent: Hmm. I really hope so. Because we can’t do this alone. The sea is too strong for us. The stones we buy don’t work, and even if they did, not everyone can afford them.

Interviewer: Is there anything else you would like to share?

Respondent: No, please.

Interviewer: I hope your voice reaches the right people. Thank you for sharing that with me. I want to make sure your voice is heard, and your story is told. Thank you. We are very grateful. Thank you so much.

CLIMATE MENTAL HEALTH INITIATIVE

TRANSCRIPT

INDIVIDUAL INTERVIEW_2

Date: 15th February, 2024

Transcription code

(…) – Incomplete sentence

(xxx) – Not audible

[overlapping talk begins

] overlapping talk ends

(..) pause

‘Dressss’ lengthening of a word

Becau – cut off, interruption of a sound

I DON’T – Loud sounds/words

(Left hand on the neck) body conduct

Interviewer: Second author

Interview starts – Participant 2

Interviewer: Good morning and thank you for taking the time to speak with me today. We're here to discuss some of the important issues affecting this community, particularly related to the ongoing sea level rise and its effects on the mental health of residents in this community. We're hoping to get a deeper understanding of how these changes have affected daily life, the environment, and the livelihood of the people who live and work here.

Respondent: How old are you

Interviewer: 35 years

Interviewer: Have you noticed any sea level rise over the past 10 years?

Respondent: Yes, over the years , I have definitely noticed the changes. The sea has been encroaching further onto the land, and the water levels seem to rise higher than they used to. If you

look at this community you'll see how the sea has affected the land, and many houses and shops have collapsed into the road. The storm hit our houses gradually at first, and over time, they just couldn't stand it anymore. Eventually, the buildings collapsed, and now the water is right up to the road.

Interviewer: How has the sea level rise affected you?

Respondent: I sit outside for too long. Because I'm afraid, if I go inside, I don't know what will happen to me. So, I prayed that God would make me like that woman… that three disciples who were sent to look after something in the house... I have forgotten the quotation but I'm unsure if you know what I'm talking about. He hid them and when the people got there, they couldn't find them.. and later they destroyed the city, but their house was not destroyed.

Interviewer: yeah

Respondent: that has been my prayer... (Baby cries) Stop it ah.. that God should hide me like that.. Until I'm able to get a place, and if it destroys then I know I'm gone. To be honest, it's God that keeps us going. We turn to God because sometimes there's nothing else you can do. When the storms hit, we pray for strength, and even when we lose our homes or livelihoods, we trust that God will provide a way. It's not easy, but our belief keeps us hopeful. We know we can't control the sea, so we leave it in God's hands and do our best to adapt.

Interviewer: you are gone?

Respondent: I'm gone (laughs)... So… I know God listens to my prayers. Because I was living with this sister… So now that is what we are looking forward to: God help us get money to rent a place. I know the people whose houses have been destroyed by the sea moved to live with their family members in Accra. They are no longer here because their family members supported them with accommodation.

Interviewer: When are you expecting the money

Respondent: Oh we are working on it.. God will make a way. Now the sea has moved a little far away.

Interviewer: are you sure?

Respondent: hmm.. Hmmmm! You can ask sister (*name hidden)*; it wasn't like you came to meet today. Honestly, it hasn't been easy, but we're doing what we can to cope.

Interviewer: So you pray it goes down

Respondent: It has moved a bit far... Because it was all over this place. You couldn't have stood here.. You couldn't even see any sand in this place. You couldn't have played football... You couldn't have done anything stood over here.

Interviewer: hmmm

Respondent: the water was all over this place

Interviewer: hmmm

Respondent: But now it has settled down

Interviewer: it has settled down a little. So when you sleep, do you think about what is happening? Does it make you think?

Respondent: At first I used to, but now it is a bit okay for me... Because at first, there was too much pressure.. erhern... Because you had to quickly look for money and leave this place.. But now it looks like it has settled down a bit. Not very okay, but at least…

Interviewer: So because of that you are not in a hurry to..

Respondent: Oh not that I'm not in a hurry but the pressure at first isn't the same as it is now

Interviewer: Because from the look of things, you cannot predict

Respondent: You are right

Interviewer: So what we have to do, needs to be done

Respondent: It's true

Interviewer: Is it your own building?

Respondent: No

Interviewer: Do you rent it?

Respondent: Yes

Interviewer: Who do you live here with?

Respondent: I used to live with my son's father, but he has travelled for work

Interviewer: you were with your mother?

Respondent: No

Interviewer: Your father?

Respondent: No my child's father

Interviewer: Your child's father?

Respondent: Yes

Interviewer: But where is your child's father now?

Respondents: he has travelled...

Interviewer: he travelled? And left you here alone? He should come to take you with him.

Respondent: (chuckles) It is because of work he travelled

Interviewer: He travelled because of work? He should come and take you with him. Isn't there a room in his house?

Respondent: There is a room, but I can't... I don't know how to put it... Unless I look for another place

Interviewer: He goes and comes

Respondent: erhern

Interviewer: That is what you need to do.. Because looking at how it is, life matters more than anything else. You also have a child. What if you sleep and the water comes inside? What would you do?

Respondent: hmmmmm, The water entered the room the last time. I thought, the building was collapsing so I got up. It's my whole life being shaken. I hear the noise, I get up, and my heart starts racing because I don't know what's next. I am just worried about losing my house and my kid. It's everything.

Interviewer: The water was here?

Respondent: There was one night… The water hadn't gone far. So we were sitting here and the water was moving a building. We even thought it was moving the entire place so we had to run away... I was with the child, oh we even fell.

Interviewer: Oh

Respondent: So later we realized it was just that one only and not the whole place

Interviewer: not the whole place

Respondent: Yeah

Interviewer: where do you come from here?

Respondent: No I am a *name withheld*

Interviewer: you are a *name withheld*

Respondents: (laughs)

Interviewer: You come from the *name withheld*

Respondents: Oh no, this is where I was born but if I'm asked about where I'm from…

Interviewer: Ahaaa.. You haven't been there before?

Respondent: I have been there but...

Interviewer: So you are a Ga?

Respondent: (smiles) I am not a Ga

Interviewer: But this is where you have lived all your life. Is this your only child?

Respondent: yes, this is the only one

Interviewer: In this situation, only God can save us or the government can come in to build the sea defence. Until it is done there is nothing we both can do about it. So we can't prevent what is coming but let's look at how to.. Look at what.. other options. Because if we sit here without doing anything, we are playing with our lives.

Respondent: hmm

Interviewer: Because we don't know when it will trigger. Before you know.. The story will be… God saved me

Respondent: Yes oo

Interviewer: So, I've been speaking with people in the community, and I'd love to hear your perspective. Can we start with how you feel about the government's involvement in addressing the issues here, especially with the sea destroying parts of the community?

Respondent: Hmm... To be honest, I don't have much to say about the government. They've not done enough, but it's not surprising.

Interviewer: Not surprising? Why do you say that?

Respondent: Because that's how it's always been. They promise us things, and then when it's time to act, nothing happens. It's been the same story for years.

Interviewer: I see

Respondent: Well, they started the sea defence project some years ago, and I'll admit it helped the people of Glefe at first. But they didn't finish it. They just left it halfway and now look at us. The sea keeps coming closer, and people are losing their homes and businesses.

Interviewer: Why do you think they stopped the project?

Respondent: Who knows? Maybe the money is finished. I think the change in government. The new government didn't continue the project. After we vote for them, we don't see them again until the next election.

Interviewer: mmmm.

Respondent: It's very disappointing. People here are suffering. Some have had to move away because their houses collapsed. Others, like me, are still here because we don't have anywhere else to go.

Interviewer: I understand. How do you feel when you think about the government's role in solving these issues?

Respondent: I don't really feel anything anymore. It's like... I've accepted that they won't do much. But I still hope, somewhere in my heart, that they will wake up and come to help us one day.

Interviewer: That hope is important. If you had the chance to speak directly to someone in government, what would you say?

Respondent: I would tell them to stop making empty promises. If they want to help us, they should come and finish what they started. The sea is not waiting for themit's destroying things every day.

Interviewer: That's true. The damage is ongoing. Do you think the community has lost trust in the government?

Respondent: Oh, most people don't believe in them anymore.

Interviewer: Have there been any local efforts to address the challenges? Maybe community members coming together to find solutions.

Respondent: Yes, some people have tried. We've pooled money to buy stones and sandbags to protect our homes, but it's not enough. We need machines and proper engineers to do the work. This is not something we can solve on our own.

Interviewer: It sounds like the community is doing everything it can with the resources available.

Respondent: We are. But it's not easy. People are already struggling to feed their families, so how can we spend all our money on something the government should do?

Interviewer: That's a valid concern. What do you think the government should prioritize if they decide to help?

Respondent: They should complete the sea defence and make it stronger. That's the main thing. If they don't, more houses and shops will collapse. People will have to leave this place.

Interviewer: If they do come to help, how would that make you feel?

Respondent: I'd feel relieved. It would show that they care about us, even a little. It would also give us hope that things can get better.

Interviewer: And if they don't?

Respondent: Then we'll keep struggling like we always do. It's not like we have a choice. Life goes on, with or without them.

Interviewer: I see.

Respondent: It is. But in this life, you can't give up. We just pray that God touches their hearts to come and help us.

Interviewer: Thank you so much for sharing your thoughts. Is there anything else you'd like to add?

Participant: Just that I hope someone listens to what we're saying. We can't keep living like this forever.

Interviewer: Is there anything else you would like to share?

Respondent: No, Please

Interviewer: I hear you, and I hope so too. Thank you for your time and honesty.

CLIMATE MENTAL HEALTH INITIATIVE

TRANSCRIPT

FOCUS GROUP DISCUSSION 1 (Participant 3)

Date: 15th February, 2024

Transcription code

(…) – Incomplete sentence

(xxx) – Not audible

[overlapping talk begins

] overlapping talk ends

(..) pause

'Dressss' lengthening of a word

Becau – cut off, interruption of a sound

I DON'T – Loud sounds/words

(Left hand on neck) body conduct

Interviewer: Second author

Interview starts – Participant 3

Interviewer: [all Introductions and information on age and gender were done before recording] Good morning, everyone. Thank you all for taking the time to join this important discussion today. As discussed, I'm here to facilitate this session as part of a broader initiative to understand the challenges that our communities are facing due to environmental changes and their impact on our daily lives.

Respondent 1: we want money, I hope you will give us money.

Interviewer: We will talk about the money after the meeting. We are aware that many of you have been living through significant changes in this area, particularly with the rising sea levels and their devastating effects on your homes, businesses, and livelihoods. Today, we want to hear directly from you about how the sea's encroachment has affected your lives and your communities. This focus group will provide an opportunity for all of us to share our experiences, concerns, and ideas on how we can better cope with these challenges. There is no right or wrong answer here – this is a space where we can be open and honest about the struggles, the fears, and the hopes we have for the future. We know that many of you have been personally affected, whether it's through the loss of your homes, your businesses, or even the trauma of watching the land you once knew slowly disappear. We also understand that many people in the community are concerned about their safety and the future of their children, so this discussion will be focused on those aspects too. Feel free to share what you've been experiencing – your thoughts, your worries, and your hopes for what can be done to improve things. Together, we will explore potential solutions, talk about what kind of support you need, and see how we can collaborate to help make life better for everyone. Feel free to share any personal stories or thoughts you have on the situation. Once again, thank you for being here.

Interviewer: Can you share your thoughts on the current situation regarding rising sea levels?

Respondent 2: Uh, yeah, of course. You know, I've lived here my whole life, and seeing the water closer to our homes was just… I'm still in shock about how quickly things have changed. Just a few years ago, the water was really far from our homes. If someone told you the sea would be this close, it would've been a lie. Honestly, I still can't understand.

Respondent 2: Oh, truly. I used to have a thriving business here,like, I worked so hard for it,but everything was washed away in just one storm. All the houses on this lane? They've been

destroyed by the sea. It's just… heartbreaking, you know? Seeing everything we built just disappear like that. We are losing our homes and businesses. Now it feels like everything is crumbling around us. The rising sea level has changed everything for us, and it's hard to see a way forward… Do you know?

Respondent 3: It's like we were watching our lives change before our eyes, and there was nothing we could do to stop it. I felt so many emotions anger, sadness, frustration… even guilt sometimes. Why did I even decide to leave and build here in the first place? Why is this happening to us currently? And the annoying part of it is that the government is not helping us at all.

Respondent 4: I agree with all that my people have said. We are really suffering here. We need help. We need money too.

Interviewer: Thank you all for sharing your thoughts and experiences. What we are hearing is the deep sense of shock many of you are feeling. I can hear the emotion in your voice when you talk about how things have changed over the years. It's clear that this wasn't something you could have predicted. For many of you, the water used to seem distant, peaceful even, but now it's a constant threat. It's hard to believe that the sea, which once seemed so far away, is now so close. It must feel unsettling, especially when you add the thought. I know this is difficult, but by sharing, we can better understand what is really happening here.

Respondent 4: Is the money we want? We need money too.

Interviewer: **(laughs)** The money will come. mmmmmmmmmmmmmmmmmmm How has this affected your community? I want us to dive deeper into these feelings and thoughts. How have these changes affected your sense of security and your daily lives? How do you cope with the fear that the sea might come closer tomorrow? Let's also talk about how this shift has impacted your livelihoods, your relationships with neighbours, and the future of this community.

Respondent 4: We tried to prepare for the storms by using sucks and stones to protect our homes but deep down, it felt like we were fighting against something much bigger than ourselves. The water is strong and when it decides to destroy something nothing can stop it. People whose homes have been destroyed have left this community and we don't even know where they are. Some have relocated to their hometowns and others have rented in Accra. The idea of leaving their homes,

jobs, memories, and everything they have built is incredibly difficult to fathom. It became clear that staying here was becoming too risky.

Respondent 5: This change has affected us all in ways that are hard to put into words. The community has gone through a lot, and we are all struggling in our own ways. Our homes, our businesses, and the land we used to be so proud of are slowly disappearing under the sea. I remember when I first moved here, the sea was far away. It felt safe. We could go about our daily lives without worrying about water creeping up on us. But now, every day feels like a countdown. The water used to be far from us, but now it's so close, and it's hard to ignore the fact that we could lose everything at any time. The water was very far from our homes… the water was not this close to us. If someone told you the sea would be this close, it would've been a lie. That's how I feel every time I wake up and see the sea creeping closer to my doorstep. It's like we've been living in a dream, thinking this would never happen. But now, this is our reality, and it's difficult to accept. The impact on our businesses has been devastating. I used to run a small business, and many others did too. But with the water so close, people aren't coming to buy like they used to. Some of us have even lost our shops, our homes, and our livelihood. Some have moved to other places because it's too dangerous to stay, and I can't blame them. The entire community is now in a state of shock. I've watched many of us lose everything. That's the reality for so many of us. We've worked so hard to build lives here, and to watch it slip away like this, it's heartbreaking. The damage isn't just physical, it's emotional too. People are scared. We don't know what tomorrow will bring. I used to feel safe, but now every time the waves come in, I wonder if this will be the last time my house will stand, or if I'll have to find somewhere else to go. That's how it feels every day now,like we've been blindsided. I don't know how long we can keep going like this. The sea doesn't wait for anyone. It keeps coming, and we can't stop it. So, we hold onto hope that maybe things will get better, but it's hard to see a future here when everything around us is disappearing.

Interviewer: what other way do you cope with this situation?

Respondent 3: We also reached out to the MP and the government for support in this process. However, it's still a complicated issue because of politics. Not everyone can afford to move or wants to leave their homes, so we use the stones in the meantime and pray God protects us.

Respondent 2: It's really tough, and coping is not easy. But we try to make do with what we have. Some of us have started to look for other ways to survive, given that our usual businesses are no longer thriving. I also try to save whatever little I can. Some of my neighbors have moved away. They went to live with their family members in Accra because their families were able to support them with accommodation. It was a difficult decision, but it was the only option for them,

especially when the sea kept encroaching on their homes. They couldn't bear the thought of losing everything, so they left to start fresh elsewhere. As for me, I've stayed here. Some people come by from time to time to encourage me and offer support. They tell me to stay strong, and that things will get better. It helps, but I can't help feeling lonely sometimes. Still, the people who visit remind me that there are others going through similar challenges, and we're not alone in this fight. I'm grateful for their visits, but I wish the situation was different for all of us.

Respondent 6: Every day, we pray that the sea doesn't take what's left of our homes and businesses. It's about holding on to hope, even when everything feels uncertain. We use bags of sand to try and block the water from getting closer to our homes, and some of us even spend money to buy rocks for protection. It's not a perfect solution, but it's the best we can do for now. I am forced to spend money to buy rocks to protect my house. We do what we can, even if it doesn't stop the problem entirely. The future is uncertain, and the sea doesn't wait. We just keep hoping that somehow, things will get better.

Respondent 7: My mother has refused to buy new goods for the shop because she doesn't know when the sea might destroy it. She's scared, you see. The water keeps creeping closer, and every day feels like a gamble. We don't know if tomorrow the day will be the shop will be washed away. I understand her decision, though. Why invest in new goods when everything is so uncertain? It's heartbreaking, but we have to make these tough choices to survive. Luckily, my mother has another shop in a safer area. If the sea destroys this one, I'll move to the other shop and continue running the business from there. It's not ideal, but it's the only option we have at the moment. We have to be prepared for whatever comes our way, and having that backup gives us some hope, even though it's not the same as having both shops thriving here.

Respondent 2: Now, everyone knows the pain of loss has taken over their lives. It's not just physical; it's deep within. The constant worry, the fear of losing everything, has become a daily burden. For many, it's like a heavy weight on their heads. You can see it in their faces, the stress, the exhaustion. Their hearts are in pain because everything they've worked for, everything they built, is slipping away. I can see it in the faces of my neighbours, too. They've lost their homes, and their businesses, and they feel like they're stuck in this cycle of uncertainty. Some people try to stay strong, but you can tell the toll it's taking on their health. It's not easy. And it hurts more because we don't have control over it. We're at the mercy of the sea, and every day we're left wondering when it will be our turn. So yes, the loss is real, not just the material things, but the emotional weight that comes with it. Everyone feels it. And it's hard to escape it.

Respondent 7: I pray to the sea every day, asking for more time. Time to stay here until I can gather enough money to leave. You know, sometimes it feels like my whole life is hanging by a thread, and I just need a little more time to sort things out. But deep down, I know that no matter how much I pray, I can't control the sea. It's in God's hands now. We all pray that the sea doesn't

destroy our homes, and that it won't take everything away from us like it did to others in this community. At the end of the day, it's our faith that keeps us going. God is the only one helping us now. Sometimes, I wonder how people who don't believe make it through this. It's the prayers, the hope, and the belief that we won't be forgotten by God that gives us strength to face another day. I can't rely on anyone else to fix this for me, but I know that God will provide. Whether it's the sea, the land, or the storms we face, my trust is in Him.

Interviewer: Thank you for sharing that. It's clear that despite the challenges, you've found strength in your family community and God. Now, I'd like to ask, apart from support, if have you received any help from the government or other organizations.

Respondent 3: If the government continues with the sea defence project, I truly believe everything will be okay. We have seen the impact of the rising waters, and we know that without proper protection, it will only get worse. We are all pleading with the government to finish building the sea defence. It's not just for me but for all of us here. The water is taking everything we've worked for, and every day we wake up unsure if our homes will still be standing by the end of the day. We can't keep relying on the little measures we have now, like the sandbags and rocks. Those are temporary solutions. What we need is real, lasting protection. If the sea defence is completed, we can start to feel safe again. We hope and pray that the government listens to us, completes the defence, and ensures that we can live and work in peace without the constant fear of losing everything to the sea.

Respondent 1: Some people do come around, you know. They come, talk a little, take pictures, and maybe even ask how things are, but nothing happens after that. They just leave, and it's like they're doing it for their own benefit, not for us. That's why some of us don't want to talk about this sea issue anymore. It's really annoying. They come with all their cameras, take some nice shots, and then just leave. It feels like they're not really here for us. They don't see what we're going through. No real help comes from it, and that's why many of us are just tired of talking about it. Nothing ever changes.

Interviewer: I can imagine how frustrating that must be for you. When people come around, take pictures, or talk about your situation but nothing actually changes, it can feel like they're just passing through without really understanding or helping It's understandable that you feel frustrated, and I appreciate you sharing this with me. Does anyone want to share anything else?

Respondent 1: No, we want to go and continue what we are doing

Interviewer: Thank you all for your openness and participation in today's discussion. You've shared powerful insights.. We've heard your concerns about the lack of support, the frustration with empty promises, and the emotional toll this situation takes on you and your families. Your resilience and hope, despite the difficulties, are truly inspiring. But it is evident that more needs to be done, and

it is essential that the right actions follow the discussions. This conversation is just a starting point for bringing attention to these pressing issues, and we must continue to advocate for support. Once again, thank you for sharing your experiences. We hope that your voices are heard.

[All participants agree to end the session]

CLIMATE MENTAL HEALTH INITIATIVE

TRANSCRIPT

FOCUS GROUP DISCUSSION (Participant 4)

Date: 24th February, 2024

Transcription code

(…) – Incomplete sentence

(xxx) – Not audible

[overlapping talk begins

] overlapping talk ends

(..) pause

'Dressss' lengthening of a word

Becau – cut off, interruption of a sound

I DON'T – Loud sounds/words

(Left hand on neck) body conduct

Interviewer: Second author

Interview starts – Participant 4.

Interviewer: (Allow participants to introduce the background.) Good morning, everyone. Thank you for joining today's discussion. I am grateful for the opportunity to sit down with all of you today. Ad discussed, we're here to talk about the challenges you're facing in your community, especially as it relates to the rising sea levels, and how it's affecting your homes, businesses, and overall well-being. Our goal for today is to have an open, respectful conversation where everyone can share their thoughts, concerns, and ideas. This is a safe space where you can express how these issues have affected you personally and collectively as a community. Please feel free to speak openly, and know that your voices are important. There are no wrong answers here. We are just here to learn from each other and understand the issues better. This conversation will be recorded, but only for research and documentation purposes, and your identities will remain confidential.

Facilitator: To get started, could each of you tell us about your experience living in this community? How long have you been here, and what changes have you noticed over the years?"

Participant 1: I've been in this community for about 15 years. The water was far, and we never imagined it could come this close. Over time, the sea started to rise, and slowly, it began taking people's homes and businesses. Now, every time it rains, I fear for my shop and my house. I never thought I'd see this day. I grew up in this community, the sea was not close to us. But now, it is very close to our homes. During the rainy season, it comes to our homes. It's worrying because we don't know how long this will last. Many of the community members have already left because they couldn't take it anymore. Those who could afford it have relocated to other towns or moved in with family members elsewhere. Some of us want to leave too, but we don't have the means. It's not like we want to go, but what choice do we have when the sea keeps destroying everything? If this continues, the whole community will be empty. People can't keep living in fear, and it's hard to see a future here with no help in sight.

Participant 2: I've lived here for 20 years now. When I moved here, the land was dry, not like this. We used to have business was thriving. But in the last 10 years, things started changing. The water started creeping up on the land, and little by little, it destroyed people's shops and homes. The sea didn't used to come this far. But now, it's hard to even farm here, and sometimes, the sea floods my house. It's hard to even sleep at night.

Participant 3: I've been here since I was a young girl, so I've seen a lot of changes. The water used to be far, far away, so we could have our beaches and houses near the shore. But as time passed, the sea started to get closer. At first, we didn't notice it much, but now the waves are almost at our doorsteps. The storms have also gotten worse. We have to constantly fight against erosion. I'm worried about the future. Even now, those of us staying here are just holding on because we have no alternative. If we had the money, I'm sure most of us would have left by now. Every time the sea rises, it's like a reminder that staying here isn't safe.

Participant 4: Ah, I've been living here for over 30 years. When I first came here, the place was so quiet, and peaceful. The sea was far, far away. We could build our homes without fear. I used to work, and the business was good back then. But over the years, the water has slowly been coming closer and closer. At first, it was just small waves, nothing to worry about. But now, it's really affecting us. My house is no longer safe, and the fish catch is smaller. I don't know what will happen next.

Facilitator: Many of you have spoken about the rising sea levels and how they've been affecting your homes. In such a difficult situation, we often look for ways to cope. Can you share with us what steps you've taken to protect your homes and businesses? What strategies are you using to manage the challenges you're facing?

Participant 2: Well, we have no choice but to find a way to survive, right? For me, I've had to move my important things to higher ground inside my house. I even put big rocks around the house to try and block the water from coming in. It's not easy, but what can you do? My business is suffering because of the sea, so I've started selling small items in my shop, things that

people still need, like food and soap. It doesn't bring much, but at least it helps me get by. We also pray to God every day for the water not to rise higher.

Participant 3: I have been trying to protect my house by using sandbags around it, filling them with sand to stop the water from coming in. It's hard work, but I don't want to lose everything. I've also been trying to keep my business running by moving things to safer areas when it rains heavily. I know I can't stop the sea, but I do what I can. Some of the younger ones have been helping me, but they are also struggling. I've been asking the community to help with gathering rocks and sand for the defence, but we don't have enough support from the government yet."

Participant 4: Honestly, I've tried everything I can think of. I've been putting big stones near the edge of my yard, and I've used bags of sand to hold back the water, but it feels like it's never enough. The sea keeps coming closer. As for my business, I've had to stop doing the farming I once did. Now, I sell small household items in my shop to survive, but that too is becoming hard with the water flooding around. We are all waiting for the sea defence to be completed, but it's not moving fast enough. I just hope it's not too late for us. Sometimes, I also think that, this government won't do the sea defence for us. They keep making promises, but nothing happens. We've waited too long, and we can't keep living like this. As a community, we've decided that if they won't help us, we will vote them out. Maybe the next government, under a different political party, will take our cries seriously and come to our aid. We need leaders who will act, not just talk. Enough is enough.

Participant 5: The thing is that protecting my business has been really tough. I've been shifting my things around, trying to move anything valuable to higher ground when the water rises. I've also joined the other shop owners in putting up sandbags to protect our property, but it's not easy. We have few resources to buy stones or pay for proper defence measures. Sometimes, I wonder if it's even worth it because the water seems to keep pushing further. But we continue, hoping the government will come through with the sea defence project. In the meantime, I'm praying that things don't get worse.

Participant 4: exactly as my brother said**.** I agree with my brother**.** My biggest hope is that the sea defence project will be completed soon. If they finish building the sea wall, it will help us a lot. Right now, every time it rains heavily or when the tide comes in, we're all living in fear that our homes will be washed away. I just want to live without worrying constantly about the water rising. If I could change one thing, it would be to have more support from the government for our businesses and our families. They should help us protect our livelihoods better, and also make sure we have the resources to rebuild after every disaster.

Participant 2: My hope for the future is simple – that the water stops coming closer to our homes. We've seen so many people lose everything, and it's painful. I just want the younger generation to have a better future here. If I could change one thing, it would be to have a proper sea defence that really protects us, and I mean something strong enough to withstand the tides. I also hope the government can provide more support in terms of education and job opportunities so that we don't have to rely so much on the sea and the land. A strong, united community is what I dream of for the future. The people in Glefe who have the sea defence are not any better than us. Look at their situation, they are still struggling with the water, just like we are. The sea

doesn't choose; it affects all of us. We all vote in this country, so why should some communities get help and not others? We deserve the same level of protection and support. If they can defend Glefe, then they can defend this place too. It's only fair.

Interviewer: Is there anything else you would like to share?

Respondents: No, please.

Facilitator Closing Remarks: Thank you all for your time today and for sharing your experiences with such honesty. Your voices are incredibly important in understanding the depth of the challenges you're facing and in highlighting the need for more support and action. Thank you again for your participation.

CLIMATE MENTAL HEALTH INITIATIVE

TRANSCRIPT

FOCUS GROUP DISCUSSION-4 (Participant 5)

Date: 7th March, 2024

Transcription code

(…) – Incomplete sentence

(xxx) – Not audible

[overlapping talk begins

] overlapping talk ends

(..) pause

'Dressss' lengthening of a word

Becau – cut off, interruption of a sound

I DON'T – Loud sounds/words

(Left hand on neck) body conduct

Interviewer: Second author

Interview starts

Facilitator: Good afternoon, everyone. Thank you for joining today's discussion. We are here to talk about your experiences living in this community and the impact of the sea on your lives and livelihoods. Let's create a space where we can share freely. Who would like to start by telling us how the sea has changed things around here?

Participant 1: Good afternoon. I remember when the water was very far from our homes. The sea was not this close. If someone had told me years ago that the sea would come this close to us, I would've called it a lie. Back then, we could walk for miles before even seeing the shoreline. The sea was not this close. It felt unimaginable, but now, here we are. The water is almost at our doorsteps.

Participant 2: That's true. We never thought the sea would get this far. Now, all the houses on this lane have been destroyed by the sea. My own house was washed away completely. Now,

everything has changed. The sea has destroyed the land we used to live and work on. We've lost so much.

Participant 3: I used to have a thriving bar business here, but everything was washed away. My business collapsed. I've tried to restart with the little money I had left after the sea destroyed my house, but it's been tough. No one is helping us with this water issue. It's hard knowing that no matter how much effort you put in, the sea can take it all away in an instant. I've tried to restart with the little money I had left after the sea destroyed my business, but it's been tough. No one is helping us with this water issue. It feels like we're on our own

Participant 4: My story is similar. We used to run the bar business together. Everything is gone. Now, I'm forced to use the little money I earn to buy rocks and put sand in sacks to protect my house. But even with that, I don't know if the water will destroy my business tomorrow. It's a constant fear.

Participant 1: I'm scared too. I haven't slept for about a week. I'm afraid the last room I have left might collapse on my children while they sleep. That fear doesn't leave me. Sometimes, I lie awake praying that the structure will hold through the night. It's a very heavy burden.

Participant 5: Hmm, I am in sorrow. In my youth, I couldn't do anything for the next generation. What to eat is even a problem, you know. There isn't any joy left in me. I had built a shower for rental all over this place. And the sea took it all away. It was from where the storm has reached now. All over that place.

Facilitator: That place?

Respondent: Yes

Participant 5: Then it started a long time. It started a long time.. It started a long time When it started like that, they said they would give us the…

Facilitator: sea defence

Respondent 5: Sea defence, and truly, they started building from somewhere.

Facilitator: hmmm

Participant 5: Then we said no…

Facilitator: they are getting closer

Participant 5: they are getting closer. By the time they got to this place the damaged ones weren't …

Facilitator: too many

Participant 5: Sister did you pass through that place when you were coming? You didn't take him through that route?

Interviewer: Yes, I saw it. I passed there. I have just arrived

Participant 5:: The main road, the gutter before you get

Facilitator: before you get to the road

Participant 5: To the road.. It's not a small issue at all. Even sitting here right now, I don't know what will come next.

Facilitator: Because at this point it has also reached here

Participant 5: Oh1 it reached here

Facilitator: Because even at night it (Sea) will rise..

Participant 5: oh!

Facilitator: the levels will rise high

Participant 5: Mine is all finished, it's left with theirs

Interviewer: Oh so your buildings are finished?

Participant 5: oooh! Mine is all finished. It's left with my land. The pipe too is not flowing

Interviewer: hmmm

Participant 5: It's been almost a month now.. if i wake up… Where we live, there is a lot of noise which i don't like. So if i wake up then i…

Facilitator: So you don't currently live here?

Participant 5: No, no, no. We don't live here. There is nothing to live here for

Facilitator: So when it's morning then you come here

Participant 5: i come here

Facilitator: Then you come for…

Participant 5: I come… Because if you are staying here all you will do is to think about the buildings the sea has washed away

Facilitator: The case is serious oo.. It is serious

Participant 5: hmmm

Facilitator: So how many children do you have?

Participant 5: I have 11. Some are deceased. So now the remaining… i have seven men and four women. My last born is even

Facilitator: hmm

Participant 5: They are all alive

Facilitator: hmm

Participant 5: My last born is even…

Facilitator: Are they all working?

Participant 5: Yes, they are all hardworking

Facilitator: Then you will thank God

Participant 5: The reason i even thank god.. If i call them they send me something

Facilitator: Yeah, then you use it to sort yourself out.

Participant 5: yeah

Facilitator: because i was just imagining if they were all living here while it happened, how you would have survived it? eei! Because if it was human doing, you would go to stop them

Participant 5: hehe hehe hehe (laughs) that is the issue

Facilitator: but it is not the work of any human

Participant 5: it is not the doing of human

Facilitator: And if it was the work of a human, you would go to ask him.. to stop him

Participant 5: Ei. Even you, if you are not careful he can harm you

Facilitator: the sea collapses the buildings you cannot go to him to ask him why he did

Participant 5: No no. Sometime ago.. The beginning of it all

Facilitator: hmmm

Participant 5: My legs, If you observe it from afar, you can see that it.. it all got rotted.. It all rotted. It broke and twisted leaving only the bone.

Facilitator: hmmm

Participant 5: Because the flesh was torn, it was left with the bone only. It's not a small case.

Facilitator: All this because of the water

Participant 5: It collapsed on me. I didn't know it had washed away all the foundations of the building. I didn't know the depth of damage..

Facilitator: hmmm

Participant 5: There was a pathway to the other end. So, i passed through it. So, when i got to mine and I stopped to look at how, it was doing. Suddenly it, collapsed.

Facilitator: hmmm

Participant 5: My luck was.. the direction i ran to, it didn't reach there. The moment i ran to the place, my leg got stacked under the building.. There was no one present at the time, No one was there and i was suffering

Facilitator: It would have knocked you out

Participant 5: While I was running to the other side of the building, it

Facilitator: hmmm

Participant 5: I didn't know the depth of damage. The water was fast approaching. And if it happens like that it digs a large hole like twice the depth of my height.

Facilitator: it has dug a hole under the building?

Participant 5: Yes, it digs a hole.. So the building is left hanging. The hole becomes bigger and you are left hanging on the surface. So this year, while it was behaving the way it is i was clever not to give myself to it anymore

Facilitator: hmm

Participant 5: So we left there to sleep at home

Facilitator: hmmm

Participant 5: By the time we came back it had already [finished its work].

Facilitator: hmm

Participant 5: So, if I were here the things i used to fetch water were drowning.. But if i decided to chase it, I don't know what would have come out of it.

Facilitator: Yeah

Participant 5: So, I have to give thanks to God. The troubles are so many.. I just can't tell you all of them.

Facilitator: hmm.. So now, are you able to sleep?

Participant 5: I sleep, I sleep, but I get body pains because of the woods I put together and the sand I collect. Every day I collect sand…

Facilitator: hmmm

Participant 5: Every day I collect sand. I have been collecting sand to support my building.. To see if it would.

Facilitator: But all was in vain.

Participant 5: But… even now because I no longer do that, it gives me body pains. If i wake up in the morning.

Facilitator: Too much thinking could also cause that. Because from what you are saying you come to sit here watching and thinking about it.. That is what we are talking about today. That too much thinking and worrying is not the best. Right now, we don't want to lose what is left of us to survive on. That is why we are pleading with you to let go. Let's stop thinking too much.. I know it's not easy. You would come to sit here for fresh air. But because of this, you are perching with someone. You would have lived in your own house and have no problems with nobody.

Participant 5: I was with my tenants

Facilitator: You were with your tenants.. And now you are paying rent to someone

Participant 5: When it happened like that,

Facilitator:: Look at that! now all the lands are gone

Participant 5: Oh they are finished.. They are finished. Before you would be able to do something unless they.. They do the

Facilitator: Sea Defence

Participant 5: If they complete it there will be a large piece of land remaining so those of us behind, if we can get back our land

Facilitator: the land

Participant 5: Then little by little… But since we don't have it back, we can't do anything about it. Now we pray they build it for us

Facilitator: hmmm

Participant 5: That is the number one thing

Facilitator: If you have life, you have the strength to…

Participant 1: strength

Facilitator: do whatever you want to do

Participant 5: to do

Facilitator: If you work towards it

Participant 5: towards it

Facilitator:: you can do it. These are our words of encouragement to you.

Participant 5: hmm

Facilitator: While you are here, many others are still living in those buildings.

Participant 3: Yes

Facilitator:: While the sea is coming

Participant 5: yes

Facilitator: That sounds very distressing. Do you receive any support from the government or other organizations to help with these issues?

Participant 1: Some people come here to encourage us, but nothing is done. We are pleading with the government to finish building the sea defence. If they continue the defence, everything will be okay. We pray that they don't stop the work.

Participant 6: Yes, the sea defence is our only hope now. We pray that God helps us because, right now, He's the only one helping us. The government needs to act fast before it's too late.

Facilitator: Aside from the sea defence, how are people coping with these challenges?

Participant 4: Some have moved to live with their family members in Accra. They are no longer here because their families supported them with accommodation. As for me, I'm saving money to leave this community. My body is here, but my soul is not. I pray to the sea to give me some time to stay here till I can afford to leave.

Participant 7: That's what I'm planning too. I will go back to my hometown and operate my business there. This place no longer feels safe. But for now, we use sacks and sand to protect what we have left. It's not a permanent solution, but it's all we have.

Participant 8: For me, I've lost everything. My house, my small shop, even my livestock. I almost lost my legs trying to salvage some of my belongings during a flood. Now, I just sit and pray that the sea doesn't take more. I keep asking, When will this end?

Facilitator: It seems like many of you are thinking about leaving. What would it take for you to feel secure enough to stay?

Participant 2: If the government continues the sea defence, everything will be okay. We are pleading with them to finish the work. If they can protect the coastline, we can rebuild our lives here. We wrote to the municipal assembly to help us with our situation. But nobody has been here. I voted for this government, but no one is helping us with the sea issue. People come and take pictures here and they go but nothing is done. And we are still here.

Participant 3: Exactly. But until then, we have to rely on ourselves and our prayers. We just hope the sea doesn't destroy more before the defence is completed. Every day feels like we're waiting for the worst to happen.

Facilitator: Thank you all for sharing your experiences. It's clear that the sea has deeply impacted your lives, but it's also clear that you have hope and resilience. Your voices are important, and I hope that by sharing your stories, we can bring more attention to the challenges you face. Before we wrap up, is there anything else you'd like to add?

Participant 5: Just that we are begging the government to help us quickly. The sea doesn't wait for anyone, and we are suffering.

Interviewer: Is there anything else you would like to share?

Respondent: No, please.

Facilitator: Thank you. Your stories and insights will be heard. We'll end the session here, but I appreciate your time and courage in sharing today.

CLIMATE MENTAL HEALTH INITIATIVE

TRANSCRIPT

Focus Group Discussion (Participant 6)

Date: 28th April, 2024

Transcription code

(…) – Incomplete sentence

(xxx) – Not audible

[overlapping talk begins

] overlapping talk ends

(..) pause

'Dressss' lengthening of a word

Becau – cut off, interruption of a sound

I DON'T – Loud sounds/words

(Left hand on neck) body conduct

Interviewer: Second author

Interview starts – Participant 6

Facilitator: Good afternoon, everyone. Thank you for taking the time to join this discussion. Our focus today is on the changes we've been seeing in our environment, particularly how the sea has been affecting our lives. This is a space for us to share our thoughts and experiences, so feel free to speak openly. Let's start with this question: *How has the sea changed over the years in this community?*

Participant 1: Ah, where do I even begin? The sea… it was far from here when I was growing up. The water was very far from our homes. . I feel the pain every day. When the waves first came, I tried to save some of my things. I got caught in the rushing water, and I thought I wouldn't make it out. Since then, it's like the despair doesn't leave me. I can still hear the sound of the water in my head. You don't feel safe, even on land.

Participant 2: Yes, yes! The water was not this close to us. I remember as children, we would walk for a long time just to reach the beach. But now? The sea is almost at our doorsteps. I haven't slept for about a week because I'm scared that the last room might collapse on my children while they sleep.

Interviewer: Is this your house?

Participant 3: Yes, Yes, Yes. Mine was part of it too, but now it has collapsed so I had to rent. I’ve thought about starting over, but what’s the point if the land itself is disappearing? Why would anyone invest in a place where the ground beneath you isn’t even stable? It’s like we’re stuck in one place, unable to move forward.

Interviewer: You have rented?

Participant 3: Yes, so now this is where I live. If it gets here then…

Interviewer: Where are you from?

Participant 3: I’m from *name withheld*

Interviewer: So you would rent again

Participant 3: No, with the current rent rate in Accra, I can’t…If you don’t have GHS 7000 you cannot rent. Even in this area that hasn’t developed, rent is expensive.

Interviewer: In this situation, only God can save us, or the government can come in to build the sea defence. Until it is done there is nothing we both can do about it. So, we can’t prevent what is coming but let’s look at how to.. Look at what.. other options. Because if we sit here without doing anything, we are playing with our lives.

Participant 4: Hmm. It’s true. If someone had told me the sea would come this close, I would’ve called it a lie. It’s hard. I used to have a thriving business here, selling provisions. But everything was washed away when the waves got stronger. Now, I’ve had to move my stall inland, but business hasn’t been the same.

Participant 5: Same here. All the houses on this lane, where I grew up, have been destroyed by the sea. My family had to move to my uncle’s place in another town. It’s not easy. It makes me feel powerless. What can we do? The sea is bigger than us. It’s frustrating, really. Sometimes I wonder if it’s punishment for something. Why is the sea eating away at our land? For me, it’s my faith. I pray every day that things will get better.

Participant 3: Just that we need more people to hear our voices. I haven’t slept for about a week. I’m afraid. Every time the waves get loud, I think they’ll come again. I’m scared that the last room I have might collapse. I have little money now. I tried to save what I could, but the sea destroyed almost everything. I used the little money I had left to move my family inland for safety.

Participant 4: It's the same for me. Sometimes I lie awake at night, just listening to the sounds of the sea. Even when it's calm, I can't trust it anymore. Hmm, the sea doesn't just take things; it takes your future. But when the water rose, it destroyed everything. I've been trying to pick up the pieces, but the little money I had left after the disaster is barely enough to survive.

Participant 5: Yes, it does. I want to expand my business again, but I'm afraid. What if I invest all my money, and then the sea comes back? It feels safer to just do nothing. Why would anyone want to expand or invest in a place where the land itself is disappearing? We're stuck.

Participant 4: We need action, not just words. The water issue, the collapsing houses,we've been talking about these for years. We need real help. Even when things seem hopeless, I pray. I pray that the water will stop rising. I pray for strength to keep going. And I pray that one day, we'll have a better life.

Facilitator: Thank you for sharing that. As we wrap up, if there was one message you'd want decision-makers or leaders to hear, what would it be?

Participant 4: We need action. Not promises, not words,action. The water issue, the collapsing houses, the lost livelihoods,we've been talking about these things for years. We need real help. And when I tried to get help, when we all cried out for help nothing was done. No one is helping us with this water issue. We talk, we write letters, we go to meetings, but nothing changes. It feels like no one cares about us.

Participant 2: Yes, they need to listen to us. We are the ones living through this every day. we're real people, and we deserve to be heard.

Participant 4: Yes, many people have left. Some of my closest neighbors moved to live with their family members in Accra. They are no longer here because their family members supported them with accommodation. I don't blame them,it's hard to stay here when the sea keeps coming for what little you have left. But for some of us, we don't have anywhere else to go.

Participant 3: That's true. Some people come here to encourage me, saying I should think about leaving too. My mother keeps telling me to go back to my hometown. She says I should move there and operate our business there, but it's not that simple. How do you start over when you've lost so much? My body is here, but my soul is not here anymore. I'm just trying to save enough money to leave this community.

Facilitator: What keeps you here for now?

Participant 2: For me, I stay because I have no choice. I pray to the sea to give me some time to stay here till I get money to leave. It's funny, isn't it? We pray to the same sea that's destroying our homes. But what else can we do? God is the only one helping us now. Every day, I pray that the sea doesn't destroy my home.

Participant 5: (Interjects) It's not funny at all, it's survival. We all pray. We pray to God, to the sea, to anything that will listen. But prayer alone isn't enough. If they our leaders, continue the

sea defence, everything will be okay. We are pleading with the government to finish building the defence. That's the only thing that can save us now.

Facilitator: The sea defence seems like a critical solution. Do you feel hopeful that it will be completed?

Participant 1: (Shakes head) Hope? Hmm. We hope because we have no other choice. We hope that they continue to build the defence for us, but the truth is, we don't see much progress. The work is slow, and we are suffering in the meantime.

Participant 3: And while we wait, we're forced to find our own ways to protect ourselves. I have spent what little money I have to buy rocks to protect my house. Some of us use sacks, we fill them with sand to create a barrier around our houses. But how long can we keep doing this? The sea is stronger than us.

Participant 2: It's true. These temporary things don't last. I have used sacks too, but when the waves are strong, they wash everything away. It's like pouring water into a basket, you can't win against the sea.

Participant 4: The government?. We need action, not words. If they had finished the sea defence years ago, we wouldn't be in this situation.

Participant 5: Exactly! We are not asking for much,just protection for our homes and businesses. If they continue the defence and finish it, we can rebuild our lives. But every delay pushes us further into poverty and despair.

Facilitator: I hear you. As we wrap up, what message would you like to send to decision-makers about the urgency of your situation?

Participant 1: My message is simple: finish the sea defence. Don't wait until the entire community is gone before you act. We need help now.

Participant 3: And don't just finish it,talk to us. Involve us in the process. We are the ones living with the sea every day, and we know what we need.

Participant 2: Yes. Let them know that we are not just numbers in a report. We are people with families, with hopes, with dreams. We deserve to be heard, and we deserve action.

Interviewer: Is there anything else you would like to share?

Respondent: No, please.

Facilitator: Thank you all so much for being part of this discussion. Let's continue to stay strong and support each other through these challenges.

CLIMATE MENTAL HEALTH INITIATIVE

TRANSCRIPT

Focus Group Discussion_5 (Participant 7)

Date: 14th April, 2024

Transcription code

(…) – Incomplete sentence

(xxx) – Not audible

[overlapping talk begins

] overlapping talk ends

(..) pause

'Dressss' lengthening of a word

Becau – cut off, interruption of a sound

I DON'T – Loud sounds/words

(Left hand on neck) body conduct

Interviewer: Second author

Interview starts – Participant 7

Facilitator: Thank you all for joining this discussion. We're here to talk about how the rising sea levels and its effects have changed your lives. Let's begin with how this situation has affected your homes and livelihoods.

Participant 1: Ah, my brother, the water was very far from our homes. I remember when we could walk long distances before even seeing the waves. But now… the water is not this close to us anymore; it is here, right in front of our doors. If someone had told me years ago that the sea would be this close.

Participant 2: Hmm, the same here. I used to have a thriving business here. People would come from far to buy fish from me. But everything was washed away, everything. I am a fishmonger,

and my husband is a fisherman. We used to have a thriving fish business here, but the sea has washed away nine of my rooms and my fish business has also collapsed. I have little money. All the houses on this lane have been destroyed by the sea. I had to move my business into a small corner of what's left of my house, but it's not the same. …

Participant 3: The pain is too much. Now everyone knows his loss has taken over his head. It's not just about losing things; my heart is in pain every day. There are times I feel like I'm losing my mind. One time, during the last flood, I almost lost my legs too. The water swept me away, and I was lucky someone pulled me out.

Participant 4: I feel the same. My business has collapsed. I don't have enough money to restart it. The little money I have left after the sea destroyed everything has gone into feeding my children. I feel trapped. The sea doesn't stop at the shoreline anymore. It's moving in, little by little, every day. And when the rain combines with the rising tides, it's like the whole community is under attack. whole road became a river. Cars couldn't move, people were wading through water up to their knees. The houses nearby, our houses, were flooded. If nothing is done soon, the water will take everything, our homes, our businesses, our lives. It just keeps coming.

Participant 5: And it's not just about the money. No one is helping us with the water issue. They come and take pictures, they write things down, but nothing is done. Every time it rains heavily, or the tide rises, we are back to square one. You see, the problem started when they built the sea defence wall in Glefe. At first, we thought it was a good thing. It was protecting the community, holding back the waves. But what we didn't realize is that the sea doesn't just stop because of a wall. It moves. The sea defence has stopped the water from coming straight into Glefe, but now it's pushing the waves to the Shiabu, where there's no defence. And that's where the real problem is. The water is now rushing toward Shiabu, flooding homes and destroying everything. It's like the sea is angry, looking for new places to claim. In Shaibu, we don't have any walls to protect us. When the waves hit, they come with full force. People have lost houses and shops they rely on to survive. I remember last month, one night, the water rose so high it flooded the main road. The defence wall is supposed to protect, but it's only protecting one side while sacrificing the other. It's like we've been forgotten here. The water keeps coming, and with every tide, we lose more land, more homes. How can we live like this? We need the government to do something, build another defence, or find a way to balance the impact. Because right now, the wall is just shifting the problem from one place to another. And if nothing is done, Shiabu will disappear under the waves.

Participant 3: I haven't slept for about a week. I am afraid. The walls of my house are weak now, and I'm scared that the last room might collapse. Every night, I sit awake, watching the water and praying nothing bad happens.

Participant 6: It's not just fear, it's everything. I wanted to expand my small business, but how can I? I am too scared to invest what little I have, knowing the sea might take it all away again.

Facilitator: Have any of you thought about leaving?

Participant 5: (Nods slowly) Many people have already left. Some moved to live with their family members in Accra. They are no longer here because their family members supported them with accommodation. I'm happy for them, but for those of us still here, it's a struggle.

Participant 1: I've thought about going back to my hometown. Maybe I could move there and operate my fishing business. But the truth is, I don't have the money to leave. I'm saving every pesewa I can to leave this place.

Participant 3: (Nods) Me too. Every day, I pray to the sea to give me some time to stay here until I get enough money to leave. But I also pray that the sea doesn't destroy our homes before that happens. God is the only one helping us now. Some people come here to encourage me from time to time. That makes me feel better and strong.

Facilitator: now let's talk more about the defence?

Participant 7: The sea defence? Hmm, we are pleading with the government to finish building it. If they continue the defence, everything will be okay. But for now, we're on our own.

Participant 1: Yes, we hope they finish it, but we can't wait forever. I am forced to spend money to buy rocks to protect my house.

Participant 5: I use sacks filled with sand. We use the sacks and put sand in them to protect our houses. If not the sacks, the sea would have destroyed the building for a long time. But when the sea gets angry it will break the house, simple! Nothing can stop the sea from destroying the houses if it wants to. We all do. But it's a temporary solution. When the waves are strong, the sacks are no match for the sea.

Facilitator: It sounds like you're taking matters into your own hands, but it's not sustainable. What message would you like to send to the government and other stakeholders?

Participant 6: We don't believe in the government. My message is simple: act now. Don't wait until we're all gone before you complete the sea defence. Our lives and our futures are at stake.

Participant 2: (Nods) And listen to us. Involve us in the process. We know this land better than anyone. We need solutions that work for us, not just for the people in offices.

Interviewer: Is there anything else you would like to share**?**

Respondent: No, please.

Facilitator: Thank you all for sharing your stories and insights. Let's continue to support one another as we work towards a better future.

CLIMATE MENTAL HEALTH INITIATIVE

TRANSCRIPT

INDIVIDUAL INTERVIEW_8

Date: 24th February, 2024

Transcription code

(…) – Incomplete sentence

(xxx) – Not audible

[overlapping talk begins

] overlapping talk ends

(..) pause

'Dressss' lengthening of a word

Becau – cut off, interruption of a sound

I DON'T – Loud sounds/words

(Left hand on neck) body conduct

Interviewer: Second author

Interview starts – Participant 8

Interviewer: you can speak with us, right?

Respondent: yeah

Interviewer: Oh, then let's get some chairs and sit down. I can even sit here.

Respondent: ok

Interviewer: Oh, I can even sit here, Let me sit down. Let me sit down is not anything.

Respondent: Oh ok.

Interviewer: oh, you let's finish with this one first. Please do this one too we are using it for small research. I want to record it so that when we go, we can listen to it well.

Respondent: Yeah

Interviewer: Oh, I have heard you. Oh, ok thank you. So, give her a maximum volume so that she can speak well. You, it's just a short conversation. I have not stayed around the seashore before I want to know how

Respondent: You have never stayed around the seashore before?

Interviewer: Oh no. So errm as I was saying we are doing errm we want to know your experiences living by the sea. How do you cope with the sea? So, the first thing is that I thank you for agreeing to speak with us. Not everyone would want to speak with us. How the sea is disturbing you people and if there is something nice that has motivated you to continue to stay here and if something is disturbing you too, have you seen it, we want to speak with you so that you can explain how this place is. And as I said, I will record the interview so that we can go and listen to it well later. Please ok, do you agree for me to record it?

Respondent: Oh, I don't have any problem.

Interviewer: oh, ok thank you. Errm right now errm I will ask that you you I can really see you are a female. Are you married?

Respondent: Yeah, I'm staying with my husband.

Interviewer: You are staying with your husband, you are staying here with your husband, right? Please how many are your children?

Respondent: My children are two. They are two females.

Interviewer: Your children are two females, right? How old is the firstborn?

Respondent: The firstborn is 8 years

Interviewer: Oh ok. What about the younger one?

Respondent: The younger one is 6 years old.

Interviewer: 6 years, 6 years? Ok Please err what is your age?

Respondent: I'm 28 years old.

Interviewer: 28 years? Oh ok. Errm and how long have you stayed here?

Respondent: Oh, I've really stayed here for long.

Interviewer: so, if you take a critical look at it, how long have you stayed here?

Respondent: I was very young when we came here.

Interviewer: you were very young and you are now 28 years. So, let's take it like when you carefully observe have you stayed here more than 20 years?

Respondent: Oh yeah that one will be up to 20 years.

Interviewer: is that so?

Respondent: oh yeah. We have our own house here.

Interviewer: oh, you have your own house? Is your house closer to this side?

Respondent: oh yeah, it's in front there. It's just here.

Interviewer: oh really? Your house is here.

Respondent: Oh yeah. It's because my mother works here that's why I'm here

Interviewer: who built the house?

Respondent: My mother and her husband

Interviewer: And so here, let's say you are a wife or let's say a daughter or let's say a mother or what are you here?

Respondent: I'm a mother here

Interviewer: you are a mother here. Are you a mother in this house? Are you staying here with your husband?

Respondent: I don't stay at this place with my husband.

Interviewer: you don't stay here, right?

Respondent: Yeah, this is my mother's workplace.

Interviewer: ok. Errm apart from your children, apart from your children, do you have any dependent or any other child who stays with you?

Respondent: Oh no.

Interviewer: no, so you stay with only your children?

Respondent: Oh no my children only

Interviewer: ok. So, if you really observe here that you are staying if you observe the water with your eyes, you said you have stayed here all your life. So, if you observe the water with your eyes and hear that you are staying, has something changed? Have there been any changes to the sea, the climate, and what this place is like?

Respondent: For this place, at first, all this place, all this place was filled with water but a certain Assembly member came into office and built sea defence so it has really helped and this place has become nice and it has made the water gone back and the compound has become nice

Interviewer: mm so when you observe the living conditions here has it improved?

Respondent: Oh yeah.

Interviewer: Has it improved?

Respondent: Oh yeah it is not like the first.

Interviewer: so, when you observe, the climate, heat, and coldness of the weather and how the water has overflowed or how the water has risen or gone down, have there been any changes?

Respondent: eeeeeeeeeeeeeeeeeeeeeei! Oh yeah for the heat as it was it is the same. Of late, there is heat in our rooms and at the same time, what I will say that has been good here is that the water at first, the way they destroy things because of the sea defence has stopped those things in this area.

Interviewer: Since, since, the time you came to stay here and today, when you observe the heat and the air that blows, have there been any changes?

Respondent: Heat and air?

Interviewer: yeah

Respondent: hmm ok me me I can say that like it depends on every year and what it brings. So, I can say that this year is heat came so we are managing the heat like that.

Interviewer: So, what was on last year? Were you not feeling the heat?

Respondent: Last year, there was heat but it wasn't like this one, ok me like this I'm not the only one. I'm also pregnant so I don't know how some feel but me like this I can see I'm really feeling the heat. The heat is really killing me.

Interviewer: oh, ok so like you, like you, when we say you, you like you can talk for someone but has the heat become too much, or is it the same as in previous years?

Respondent: For this heat, I wish was sleeping in a fridge. I can say that like this year there is heat so we are managing the heat like that.

Interviewer: Last year you didn't feel the heat?

Respondent: Last year, there was heat but it wasn't like this. But ok me like this I'm not the only person. I'm also pregnant so I don't know how someone is feeling it but I can see that I'm really feeling the heat and it really killing me.

Interviewer: So, when you talk about yourself, maybe you can't speak to someone but for you in particular when you observe, has the heat increased or it's the same as it was?

Respondent: for this heat,, I wish I was sleeping in a fridge (laughs). I was sleeping in someone's fridge. Every day I will sleep in someone's fridge.

Interviewer: And so, you will say that you will say that, the heat keeps increasing

Respondent: yeah, the heat is high. It's really high too much.

Interviewer: So, when you observe this year and last year which one are you going to say that there was too much heat?

Respondent: oh, ok as you can see, I wasn't pregnant last year. It's this year I'm pregnant. So, it this year that I'm not really feeling comfortable at all and so I'm really feeling the heat this year.

Interviewer: This year you are really feeling the heat more?

Respondent: yeah really. I have taken my bath but still, I'm sweating eeeeeeeeeeeeeeeeish

Interviewer: Sweating. Ok and the water the water here when you observe the rising when you observe, has the water risen?

Respondent: hmm when it comes to the issues with this water, I don't really it well because we have fishermen and they know the water and sometimes when they are on the water, they say the water has gone far. Sometimes too they say it means the water has become full. The fishermen know compared to me because I don't work on the sea. They know because I don't do that work.

Interviewer: arh we were asking those of you here because you have stayed with the water for a long was it like this? Has it always been like this since time immemorial?

Respondent: oh no it was like this.

Interviewer: it wasn't like this right?

Respondent: yeah, I haven't seen this one before.

Respondent: At first the water overflows that why they brought the sea defence but still it overflows. When the water overflows, it can come to the back of the face. When they initially built the fence, it was tall but because the water has become more it has become short now.

Interviewer: So, when you observe, has the water increase or decrease

Respondent: the water has increased

Interviewer: Does it keep increasing and increasing or overflowing overflowing?

Respondent: Yeah, sometimes it decreases sometimes it decreases. The seashore has some sand but the sea overflowed and took away the sand. Sometimes it overflows and sometimes too it doesn't overflow.

Interviewer: ok. So, since you came to stay here, has the water taken you before? Has the water come to your houses before?

Respondent: where the water gets to, they have demolished all the buildings around that place.

Interviewer: ok so the water the way when you observe the water come come and sit here some. When you observe, when you observe, the water is like, like the way the water can overflow and destroy other people's houses, when you observe, does it really worry you?

Respondent: Oh yeah it was really disturbing during those times that it was destroying other people's houses because when you observe it only this place that maybe I will say because of the sea defence they've made that is why but when you go inside it very sad around the hospital, and shiabu it destroys the buildings to the roadside. Where there is no sea defence it destroys houses and it's so sad. Even with the sea defence, the water can overflow its banks. The sea defence is very helpful.

Interviewer: So, when you observe, it is the sea defence that has helped you right?

Respondent: oh yeah it's the sea defence but even with the sea defence the water sometimes flows over it to the back when the water overflows, but the sea defence has really helped because if they did not build the sea defence, like by this time the water would have flown to this side.

Interviewer: oh ok. So, as you said when the sea is angry it sometimes flows over the sea defence and when you also look the sea defence too is going down, has it brought any danger to you that in future it can worry you?

Respondent: Oh yeah, it has brought some danger to us like the way even the gutters become full when the sea overflows.

Interviewer: Oh ok. So, as you are here, are you afraid that, do you have any fear that in the future the water can sack you from here?

Respondent: We have not thought about that one because of the sea defence, I don't think someone will have that thought that they say can do that one day. What people think about is that during that time maybe it will be fine.

Interviewer: ok. Errm so before they built the sea defence here, has this water taken away your things?

Respondent: Yes. It has there was something here. There were buildings here. But the water has taken it all when it overflows its banks.

Interviewer: Have you lost anything at home because of the water?

Respondent: yeah, that was before they built the sea defence, it demolished other people's houses. But by this side, even when they built the sea defence, but still destroyed other people's houses but this side, the sea defence starts from here (pointing to the defence on the left side) but this side (pointing to the defence on the left side) to the sea defence. The sea defence sometimes goes that side, the water goes inside people's houses, and it destroys their buildings but for this side, it has

destroyed more than the other side because there is no sea defence there that is why it has destroyed the houses there. It will still continue to destroy the houses of people and stand at the roadside; you will see that the water has overflowed to destroy the houses of people.

Interviewer: hmm ok and so now don't you have any problem concerning the water?

Respondent: oh, not really

Interviewer: like you staying here with your husband and you are also raising your small children here. The behaviour of the water and what is happening, hasn't it brought any problem upon you.

Respondent: oh no for us we can say that the sea defence has helped us small but our neighbours there, we will say that the water is really disturbing them. We can say that the water is disturbing there because for us we don't have any problem.

Interviewer: so, you don't have any problem concerning the water right now?

Respondent: oh no but this side and this side that they have a problem because the water overflows there.

Interviewer: so, when the water behaves that way, what are your working issues like? Since the water started when you didn't have the sea defence and the time you had the sea defence, your working conditions and the job you have, have there been any changes?

Respondent: The time there wasn't sea defence and when the water overflowed into someone's room, the person could not leave for work.

Interviewer: and has the water destroyed jobs here before?

Respondent: Oh yeah, it has destroyed other people's jobs before.

Interviewer: I'm talking about jobs like for instance the stores here or the companies or people cannot go to work again?

Respondent: for that one, it is the people who cannot go to work because when it becomes full, when sometimes the water becomes full, it passes the lorry road and the people who work here sometimes open their water. Like the way the last time they said they opened the Weija water and that one really destroyed properties and nobody could go out.

Interviewer: hmm do you know someone who works on the water? Like those who go for fishing or something?

Respondent: For they, they are there.

Interviewer: Do you know some and do you, you sometimes hear some of them say the water has destroyed their jobs before?

Respondent: Like when the water is full, they don't go. They don't go fishing in the sea and at the same time when the water gets full too, they don't go. And so now the water is full, they won't go. They have days the check and when the water is not full, they go.

Respondent: when the water becomes fuller, and it's like they have some house there, they sometimes drag it to this side. On this side, the water is not really there but still when it's full, when they open the Weija, it sometimes blocks the way.

Interviewer: hmm do people sometimes come here to speak to you like Doctors and Nurses, do they come here to speak with you about your health and mental health issues, has someone been here like that before?

Respondent: oh no

Interviewer: Nobody like that has been here before, right? Hmm ok. So now you feel like what else do you feel like if the government wants to do something for you, what else do you feel the government can do to support you?

Respondent: oh, what we know the government can do to support us is that they were constructing a gutter that places for us that will help our environment that will help the bridge, the bridge we have been walking on to cross the water at this site. The bridge right now is spoilt. When you do the least, it can collapse, that is why they are constructing the gutter but for now they have stopped it. And the road there too to the other side too, the water has flooded the road to the extent that it is destroying. So, when the drivers go and they are not careful and they become careless, they can skid into the water and that really disturbs us here.

Respondent: And the road too is not just one one one one and also, they are like rough road, there is no coal tar on it, there is nothing on it and there are only stones stones only. When you are pregnant and you are going to the hospital, you will die on the way. When you are there and you are pregnant and you are going to the hospital, you will die on the way because that place you don't know whether it is road or something because it is like a rough road and because of the the water the holes and when the water overflows, it passes there, it passes there. And the cars too when the water overflows, they don't pass here. They pass that way and the bridge that passes to work too has now been destroyed so it's not every car that can pass on it. So, when the water overflows, some of them use this site to pass through houses to cross the water. Now the houses they pass, they have constructed gutters, but they have not yet constructed a bridge and this bridge too has been destroyed.

Interviewer: hmm wow, so for now, like you like you that you are pregnant, does it cause any fear in you about the problems you are talking about?

Respondent: Yeah, sometimes maybe you don't know where like you don't know what can happen to you on your way. Sometimes when you are there you will be thinking about if you were there like those there, you think about them.

Interviewer: so, most of the problems are affecting those at that place and those at the other place and those who are not covered by the sea defence they have most of the problems right?

Respondent: Yeah. Recently, the water there they call it something something faana. They said they call it faana, that one they don't have any bridge on it but they use ship on it and it killed a lot of children, a lot of children drowned in the water and died. They said they are how many? They are how many like six or so. Six children from school going to Faana and they were crossing the way to that place the boat subsided and they died.

Interviewer: so, when you observe, the living conditions here, when you compare it to other places in Accra's living conditions, will you say that your conditions are better than others or that the living conditions here are harder?

Respondent: Oh yeah, it is frightening. It is really frightening. That faana side is really frightening. They don't really get this at that place. They really need things. And that place is a town like this our town but they cross the water but it is not the sea. They cross it but they don't use the bridge. There is no bridge on the water xxx and something like this and those is no school at this place too so unless they go to school in the other town, Korle Bu side before they can go to school or unless last stop before they cross the bridge.

Interviewer: Before they can cross. Why do you believe there is no school here? Looking at the nature of this place and how the water.

Respondent: the water really destroys things that's why we don't have the school here.

Interviewer: The school here. So, you said as you are young, do you have any dream that in future you will leave here or you will leave and come back to this place

Respondent: Yeah, I will leave here in the future.

Interviewer: you will leave here. Why do you want to leave here?

Respondent: The environment is not good for me.

Interviewer: hmmm how how why is the environment not good for you?

Respondent: the water is destroying a lot of things that is why.

Interviewer: hmmm, do you think you can develop your future here?

Respondent: I feel can develop my future here but because of the water I can't.

Interviewer: because of the water you can’t. When you leave here, when you leave here in future will you return?

Respondent: oh yeah if this place becomes ok like the way Because if this place becomes ok like the way other places are comfortable and if this place becomes the same because of the water because of the water most of us don’t keep the environment clean. You can see that someone has done something that is not supposed to be done here. You can see that even in the park every time in the morning zoom lion sweeps on it but when it gets to afternoon, what is not supposed to be on it will be on it and because most of them pass here they see this place to be dirty so you will not get time to sweep and so I have to leave here.

Interviewer: hmm wow, so the dirt does the sea breeze bring them or it’s the people who bring the dirt to this place?

Respondent: oh, you said the air that blows from the sea or the.

Interviewer: yeah, I’m talking about the dirt which comes here, you see like you said, when they clean in the morning, by 12 it will come back.

Respondent: Have you seen that some people can go and pour rubbish into the seashore and its surroundings and when it gets inside the sea and sea returns them to the shore? For instance, they can sweep and throw the rubbish into a waste collection vehicle and the vehicle takes the dirt away, all that dirt cannot go into the gutters and enter back into the sea, and it cannot return. That is why

there are many rubbishes in the sea, but the sea has returned most of them to the shore. It depends on what we do.

Interviewer: continue, continue, continue.

Respondent: Have you seen that when we keep our environment clean, the rubbish we create will not go into the sea? When it enters the sea like that, the sea removes them and keeps the environment dirty.

Interviewer: talk.

Respondent: so that is what makes our environment dirty here. Because

Interviewer: continue

Respondent: that is why the environment is not good. Someone will say that this environment is not good for us, so I won't stay here. Becauseeee

Interviewer: Talk, talk,

Respondent: so that's why others will say Glefe, Glefe, I have to leave Glefe. It's not because of anything apart from the water and the environment. And people too as you can see, they didn't finish constructing this gutter, and the bridge too is spoilt and they are not refurbishing it and someone will even say that my child goes to school in the other town, and in case he closes and the bridge collapses and he falls in the water, what will I do? So, I have to be wild and do what to leave here. So many people can't stay here.

Interviewer: Is there anything else you would like to share?

Respondent: No, please.

Interviewer: I've heard you. Aunty, we thank you. Thank you wai.. Alright, thank you very much ok. Alright, we thank you. Maame (mother), we thank you wai.

CLIMATE MENTAL HEALTH INITIATIVE

TRANSCRIPT

INDIVIDUAL INTERVIEW_9

Date: 30th May, 2024

Transcription code

(…) – Incomplete sentence

(xxx) – Not audible

[overlapping talk begins

] overlapping talk ends

(..) pause

'Dressss' lengthening of a word

Becau – cut off, interruption of a sound

I DON'T – Loud sounds/words

(Left hand on neck) body conduct

Interviewer: Second author

Interview starts – Participant 9

Interviewer: As I mentioned earlier. Thank you for taking the time to speak with me today. We're conducting a study to better understand the experiences of people living by the sea. Which work do you do? And how old are you?

Respondent: I am 54 years old, and I do business

Interviewer: Business? Please can you explain? Which business exactly do you do?

Respondent: I have a shop that sells alcoholic drinks and beverages. I offer a variety of drinks, including malts and minerals. My shop is located right in front, and I manage everything myself.

Interviewer: Please do you have, which faith do you belong to?

Respondent: I'm a Pentecostal.

Interviewer: And so, you are a Christian?

Respondent: I'm a Christian.

Interviewer: ok. Ok.

Respondent: What I'm saying is there is no lie in it.

Interviewer: oh ok, I've really understood you.

Respondent: I have four children and three of my nephews with me, so there are seven of us together. My firstborn is in **name withheld**, my second born is **in name withheld**, and my third and fourth born are both at **name withheld**. I have stayed here to give birth to my children and take care of them. This place wasn't like this at first, but when they started the defence project, it brought a lot of pressure to those of us living here. When I look at the stones I have bought, one trip used to cost GHC 2,500, but now it costs GHC 3,000. I have bought stones for more than 40 trips, so just imagine how much that adds up to. A machine used to take 75 million, but now it takes 100 million for 10 thousand. Currently, when I look at the stones, it now requires eight trips. I only have three trips left to complete because if I don't do my best, where will my children and wife sleep?"

Interviewer: ok. Please in this community, do you come from this place?

Respondent: I'm not from here.

Interviewer: How long have you stayed here?

Respondent: 30years.

Interviewer: 30 years?

Respondent: Yeah

Interviewer: Where do you really come from?

Respondent: I'm from *Name withheld* **.**

Interviewer: oh ok

Respondent: *Name withheld* .

Interviewer: *Name withheld* .

Respondent: Yeah.

Interviewer: That is where you come from?

Respondent: Yeah.

Interviewer: And you came to stay here,what brought you to this place? Was it the seashore, or was there another reason that made you decide to settle here?

Respondent: I'm a hustler. I don't have a mother or father,I never saw them after I was born, and I had no one to cater for me. I came here to hustle when I was in class six. Back then, I used to sell yogurt. That's how I started hustling. Later, I became a mate and eventually learned how to drive. When I started driving, I realized I couldn't look down on myself. I had to push hard to make a living. So, I rented a store for 10 years for distribution. I kept hustling, and one day, the owner of the place told me he wanted to sell it. I worked hard and took loans from the bank to add to what I had and managed to buy the place. That's when I moved here with my wife and children, and we've been staying here ever since.

Interviewer: So, were you the one who built this place, or did you add to what was already here?

Respondent: Yeah.

Interviewer: hmm wow!

Respondent: I used this place as a spot so that when I earn something small from here, I can also make a little extra from there. For example, if people buy things from here, they can buy something small from the spot too. That way, my wife, children, and I can have something to live on.

Interviewer: Please let's pause so that you can attend to the man. Maybe he is buying something or something like that.

Respondent: Oh no, you can ask.

Interviewer: So, we're still on the conversation. It seems that when you came here, you've really made significant investments in this place. You've put a lot of money into it, haven't you?

Respondent: For the money, it's more than billions. When you calculate it,3,000 times 40 for three machines,it comes to 100 million a day, right? Ten thousand is 100 million, isn't it?

Interviewer: yeah, ten thousand is 100 million.

Respondent: So now, the ones I've bought,the machines that will do the arrangement,that's where the remaining money is going, so the machines can come and arrange everything.

Interviewer: ok. So now, you've been here for 30 years?

Respondent: 30 years.

Interviewer: Since you’ve been here, have you noticed any changes in the water, sunshine, or rainfall, over the past 10 years?

Respondent: The changes that have occurred are due to the sea defence project they started but couldn't continue. This has created problems because the water used to be far away, but now it seems to have been pushed closer. As they’ve pushed it, it has increased the pressure on those of us without the defence. They began building from Takoradi, and it has affected James Town and Sakomono as well. Do you understand what I'm saying? You can see that they’ve made some improvements at the beach in Sakomono, but as they’ve pushed it, it has caused more pressure on us here. Because they can’t continue the project, we are feeling the effects more strongly."

Interviewer: So, what brought the sea defence?

Respondent: About the sea defence? The MUI or MA realized that at some point, we would need a defence to protect those of us on the seashore.

Interviewer: Was the water overflowing in this place?

Respondent: The water wasn’t flowing to this place. We just saw that they’ve started the defence.

Interviewer: Did they inform this community of the reason for building the defence?

Respondent: Yes, they came to ask us if the defence would help and whether we liked the idea. We told them that we do like it.

Interviewer: why did you agree that you like it?

Respondent: The reason we said we like it is that, over time, the water might move closer. With the defence in place, it can help protect against that. Do you get what I mean??

Interviewer: Yeah, well understood.

Respondent: So, When the defence is in place, it can provide protection. So, what we are pleading with the government is to continue the defence they have started.

Interviewer: They should continue. It’s not like they should stop it; instead, they should keep going, right?

Respondent: Oh no no they should rather continue.

Interviewer: they should continue so that it protects all of you.

Respondent: They should continue so that it will protect us. That’s all.

Interviewer: They shouldn’t protect some and leave others?

Respondent: yeah, they should protect all of us.

Interviewer: I have understood it

Respondent: Because when you go, you like around Shiabu, it has collapsed all to the road.

Interviewer: has it demolished all the buildings to the roadside?

Respondent: Yeah, it has destroyed all the buildings up to the road. So, is the government just waiting for the entire country, all the people you're leading, to move away to where? That’s why we are pleading with the government to have pity on us and continue the work they’ve started. We won’t stand in their way. Do you see the company where I bought the stones from? This is the place. Back then, I had a machine that would pick you up and take you there.

Interviewer: hmm ok

Respondent: Ermm, The changes we’re seeing are because of the lack of proper defence. When the sun is shining, people used to come here to enjoy the fresh air. The air in this area is cool, and whether there’s electricity or not, someone might just want to sit by the beach to relax and enjoy the breeze. At the *name withheld* shop, we don’t allow smoking of weed or cigarettes, so people feel comfortable coming here to clear their minds. For example, after a long day at work, when you’re tired, you could come here to relax and make plans.

Interviewer: hmm Hmm, let’s also talk about rainfall. Since you’ve been living here since your childhood, have you noticed any changes in how it rains? Or is it still the same as it used to be?

Respondent: Right now, have you seen that the weather has changed?

Interviewer: The weather has changed right?

Respondent: Let’s take it back to how it used to be. In the past, like in June and July, when I was selling yogurt, that was the time it rained the most. But now, have you noticed how the weather has changed? It doesn’t even rain in June and July anymore. Instead, it rains at different times of the year, and it’s unpredictable. When I came to Accra in the ’90s, June and July were known for heavy rains, but it’s no longer like that.

Interviewer: Hmm ok. So, with the changes that have occurred and the rainfall pattern shifting, what about the sun? Have you noticed any changes with the sun shining as well?

Respondent: Oh, for the sun, it shines, it shines alright. The sun shines.

Interviewer: from your childhood and as you have seen the changes, have you seen any changes to the sunshine too?

Respondent: There have been changes to the sunshine too

Interviewer: how?

Respondent: Now, have you noticed that the Harmattan doesn’t come at its usual time anymore? It shows up when you least expect it. Do you understand what I’m saying? That’s where your knowledge and the psychology you work with are helpful. Over time, things will continue to change, so it’s up to us to adjust our standard of living. This way, whenever the timing changes, we’ll know how to adapt and live accordingly.

Interviewer: Yes, as you're talking about everything happening here, like the sea defence that was brought in, and before that, the water,have you been living here for a long time?

Respondent: Yeah

Interviewer: So, do you know some of the fishermen who go to the sea to go for fishing?

Respondent: Yeah, I know most of them.

Interviewer: Do you speak with some of them?

Respondent: Yes

Interviewer: In your conversations with them, have they been able to tell that there have been changes to their work on the sea?

Respondent: Have you seen that at first, they go to the sea every year but now the government has brought some rules that like one month they don’t go. When they don’t go, they complain but afterward, they say that it has helped them. Do you understand what I’m saying?

Interviewer: Yeah.

Respondent: Afterwards, they will say it has helped them because when they go to the sea, they get fish because, if they continuously go there like that, they sometimes remove the small fishes from the sea, but when they leave some days, you will see all the fishes are big when they bring them.

Interviewer: ok. So now the quantity of fish, they get and what they used to get at first, have there been any changes?

Respondent: Yeah, that one if I get them to answer this question, I would like it because I don’t go fishing just that I just observe with my eyes. What I have observed with my eyes is that, when they rest and later go, they get big fish to bring them. Do you understand what I’m saying?

Interviewer: Yeah

Respondent: So, they can really answer best. Do you understand what I’m saying?

Interviewer: I have understood it.

Respondent: So, they can best answer it because what I see with my eyes is what I'm saying.

Interviewer: if we put everything together, the sea defence has come, the sunshine has changed, and the rainfall season too has changed. If we put everything together and you look at what is happening here, has it left you any worries?

Respondent: All the worry is about the defence they are not continuing. That is all our problem. If they continue the defence, everything will be ok.

Interviewer: Have you lost something here?

Respondent: You said?

Interviewer: Have you lost something here?

Respondent: I used to have a thriving business here, but everything was washed away. I was the largest supplier of soft drinks and alcoholic beverages in this whole area…just ask about me…(tears)… I have lost my business, my investment, and years of hard work and dedication that went into building it. Yes, I have lost my shop. My first used to go to the front there.

Interviewer: oh oo

Respondent: Have you understood? It used to be at the front there. All have collapsed before I brought the stones here.

Interviewer: Was it caused by the water or in your mind it's because of the defence that has pushed the water?

Respondent: it has pushed the water because it has not like this since we've been here.

Interviewer: Before they brought the defence, there was nothing disturbing you in your shop?

Respondent: No, no, no.

Interviewer: hmm o ok. So, has it brought any panic or put any fear in you?

Respondent: This situation has brought fear to me. The problem is that when I go for loans to support my business, I need to ensure that my family and I have a place to live, as well as a place to sell my goods. The reason I'm struggling is that if the building collapses, where will my wife, children, and I go? Where will we stay? Where will I work? My work is here; everything I do is here, in my house. If I wait for everything to collapse, and someone has rented a place, where will they get the money to rent again? That only brings more problems. That's why I've been forced to spend all my money on this,buying stones to help with the defence even though the government hasn't been able to continue the work. So, I need financial support to keep going. I've spent all my

savings on this, and now I need help to continue. If someone can assist, I would appreciate it because doing this work requires support from others. It's not something I can do alone. I used to take loans in advance for the stones and for the well-being of the area. Even my car, which I used to supply goods, was taken as collateral for an advance loan. Now, I can't use the car for supply anymore because they've taken it. If it weren't for what happened, the sea defence situation wouldn't have been like this. I've been working for 12 years without any major problems, but when I asked for help, they told me they couldn't assist. And another thing,when I work with you, you deduct insurance from me. But if something goes wrong with my work, why can't you help me? Why do they take insurance payments from us, yet when there's a problem, they say they can't assist? Why are they cheating people like us who haven't had the opportunity to go to school further? Why?

Interviewer: Hmm so I will say it like this your children, do you fear for the future of these children?

Respondent: You said?

Interviewer: Do you have any fear for the future of your children here?

Respondent: Oh, if the government only comes to do the defence for us, I won't fear. If they can complete the sea defence, then I won't have any fear. The defence will protect us because, if not for the defence that pushed the water back, this place wouldn't be like this. So, if the government can finish the defence to help us, there will be no more fears. As for the children, we need to take care of them so they can have a better future.

Interviewer: Do you sometimes get some leaders in government or health workers like Doctors and nurses leaders to speak with you to comfort you?

Respondent: They don't seem to care about us. My MP is **name withheld** , and I'm also a member of the **name withheld** and a chairman. I've brought these stones here, but why can't any leader help by sending a machine to arrange them? When you ask, nobody listens. Yes, we're speaking the truth. You're recording me, and I'm telling the truth. I don't fear. My MP's **name withheld**.

Interviewer: hmm….. Have you noticed how the situation in the community, as you mentioned, is affecting your brother? It's causing him stress and leaving him in a dilemma. He has lost something important. With the ongoing work here, have you observed that the community is making people have sleepless nights or causing other difficulties?

Respondent: That's a lot to take in because when you go further to Shiabu, you'll see that all the shops and houses there have collapsed. Let me show you or send you the evidence so you can see it for yourself. It has all collapsed up to the road. I'll take the photos and send them to you right now. For me, whatever I say, I have evidence to back it up, and that's why I'm saying it,otherwise, I wouldn't have mentioned it.

Interviewer: So, the government, apart from doing the sea defence for you, what else would you like the government to do that will help the community here?

Respondent: For our community, a crucial focus should be on defence. We urge the government to continue with the defence initiatives that have already begun. The progress seen in Takoradi and the developments from Takoradi to Axim demonstrate the effectiveness of these efforts. It is essential that the government continues with Phase 2 of the defence plan, as Phase 1 has already been implemented successfully. By completing Phase 2, we can ensure that our community has a safe place to live and work. Access to a secure selling space is vital for our livelihoods. Without a place to sell goods, it becomes challenging to think about how to support ourselves and pay taxes. We respectfully ask the government to prioritize and expedite the continuation of the defence plan for the benefit of everyone in our community.

Interviewer: hmmm, Father, we thank you

Respondent: I also thank you.

Interviewer: It's like what is happening….

Interviewer: so let's go on and continue

Respondent: I'm at Dansoman Beach. I started this beach in 2011, and it was big and nice. However, the problems began in 2018 due to the sea defence project that started in Glefe. The Glefe community has been suffering because the pressure from the waves has increased. We heard that they would construct a sea defence to protect us from the waves. They began the project and reached the Royal Hospital, but now it has stopped. I went to ask the contractor what was happening, and he said the defence work would continue. I expressed my concern that stopping the project would negatively affect us. I've spent my whole life here, and I need this project to proceed. The contractor mentioned that the project is not funded by the government but rather by the World Bank. If that's the case, where is the money? They claim there are funding issues, but I don't understand why that is the case. Recently, I saw the Minister **name withheld** with the contractors here, discussing the project. I asked them about the impact of halting the defence work on our community, especially on the fishermen. Since the work stopped, the waves have become a serious problem, directly affecting my business. My hotel is suffering; it's a significant issue for me. The rooftop area I had is no longer usable, and I'm really concerned about the future of my business.

Interviewer: ok

Respondent: I started with just a small investment, and it has affected me greatly. I have lost a lot, including my properties. I had to sell my house and my land to keep things going.

Interviewer: so, at first you used to have a beach house,

Respondent: Yeah

Interviewer: And the sea came for it,

Respondent: Yes

Interviewer: How did that make you feel?

Respondent: I feel bad

Interviewer: Tell us about it.

Respondent: Yeah, I feel bad because all my resources I invest here all that I have...

Interviewer: Does it make you think a lot?

Respondent: Right now, I don't have any problems because I focus on what I can control. I'm grateful for my life and have a positive mindset as I plan for the beach. Every morning, I make it a point to clean the area and keep the beach tidy. I've seen many beautiful beaches during my travels, and as a Ghanaian, I believe I can do something better here too. I've started again, and I know this will benefit the community.

Interviewer: Is there anything else you would like to share?

Respondent: No, please.

Interviewer: Thank you so much. Have you got it? I think it's ok

CLIMATE MENTAL HEALTH INITIATIVE

TRANSCRIPT

INDIVIDUAL INTERVIEW_10

Date: 25th May, 2024

Transcription code

(…) – Incomplete sentence

(xxx) – Not audible

[overlapping talk begins

] overlapping talk ends

(..) pause

'Dressss' lengthening of a word

Becau – cut off, interruption of a sound

I DON'T – Loud sounds/words

(Left hand on neck) body conduct

Interviewer: First author

Interview starts – Participant 10

Interviewer: you can speak with us, right? Good afternoon! Thank you so much for taking the time to speak with me today. Before we start, I'd like to explain a bit about the purpose of this interview. I'm here to understand the challenges your community is facing because of the sea's rise and how it impacts your livelihood. Your story will help us bring attention to these issues and possibly find solutions. Is it okay if I record our conversation?

Respondent: Good afternoon. Ah, okay, that's fine. You can record. We are happy to share, but please don't forget about us after this, eh?

Interviewer: I understand, I'll do my best to make your story heard. Before we dive in, let me start by asking some background questions. Can you tell me your age, and what you do?

Respondent: I am 45 years old. I used to live here, and I run a small business. Now, I live in another town. I came here to sell and go back. I sell provisions, fish, meat and others. That's what I've been doing for years now.

Interviewer: Thank you!

Respondent: Hmm.... I have been here my whole life. I know this community very well.

Interviewer: Can you tell me a bit about your business?

Respondent: Yes, I have two shops. I have one here and another one in the main town where I live. The second shop is right in front of my house. I sell provisions there as well. I run this business with my daughter, who is still in school. She comes to support me sometimes when she's free. When she's around, she manages this shop, and I take care of the one at home. She has been very helpful. Honestly, if not for her, I wouldn't have a thriving business. She's so supportive, and I'm very proud of her. I've made her understand that her support is important because, without it, I wouldn't be able to pay her school fees. She takes it seriously, and she's always willing to help. She even comes up with new ideas for the business sometimes. She's truly a blessing, and I'm grateful for her.

Interviewer: tell me more about this community? I can see your shop is at the verge of the collapsing

Respondent: Ah, my sister, this community is not what it used to be. It used to be a vibrant place, full of life, with so many businesses thriving. People came from far and near to buy goods here, but now everything is collapsing, literally. You see, the sea has been our biggest problem. It's eating up the land, and many buildings and shops have been destroyed. Even my shop, as you can see, is at the edge of collapsing. I am always afraid that one day I will come here and find that the sea has taken everything away. Before, we didn't have this much trouble. The sea wasn't coming this close. But in recent years, it's like the water just keeps moving closer and closer. The rains come, the waves get bigger, and it's washing everything away. Hmm... the sea is worrying us too much. Sometimes it comes and takes the sand away, and the ground becomes weak. Even my structure here,it's not safe.

Interviewer: I'm so sorry to hear that. Do you feel like there's anything you can do to protect your business?

Respondent: What can we do? Some people buy stones to block the water, but the sea still finds its way. Even if you put sandbags, the waves just wash them away. And not everyone can afford to buy stones. Only the people with money can do that. We, the small businesspeople, just watch and pray. You see my shop is empty. I used to have a lot of things in here,provisions, drinks, toiletries, even some small household items. But look at it now, it's very empty.

Interviewer: What happened? Why is the shop so empty?

Respondent: Hmm, I have moved most of the things to my other shop in the main town. You see, I don't trust this place anymore. The sea keeps coming closer and closer, and I don't know when it will finally destroy this shop. As I'm here, I don't even know what will happen tomorrow.

Interviewer: I see.

Respondent: It is. Let me show you something, come and see. Look at the foundation of this shop. It's off! The ground has been eaten away by the sea, so the shop is just hanging. Can you imagine? A whole building, just hanging there. That's why I only keep a few things in the shop now. If I lose those too, I don't know how I will survive. The sea is unpredictable. Sometimes it's calm for a while, and then, without warning, it comes aggressively. That's why I've moved most of the items to the other shop. I only keep the basics here, things people need urgently, like bread, canned goods, and water. It's too risky to keep everything here.

Interviewer: How long has it been like this?

Respondent: Oh, it's been getting worse over the past six months. Before, the sea was far from here. It wasn't this bad. But now, every high tide, the water comes closer and takes a little more of the land.

Interviewer: So, you've had to take precautions to save your business.

Respondent: Exactly. That's why I moved most of the goods to my shop in town. At least that place is safe for now. My daughter helps me run that one. But even this, I don't know how long I can keep it open.

Interviewer: Do customers still come here?

Respondent: Not as many as before. People from the mainland, where the sea has yet to destroy their homes, come and buy from here. The business here is good, but the only problem is the water. That's why I can't close this shop. The business here is not excellent, but it's good. People do buy. Honestly, it's stressful running two shops, especially with the constant fear that this one might not survive. But what can I do? The people in this area depend on me. If I close this shop, they'll have to go very far to buy things. So, I keep it open for now, even though it's not as profitable as it used to be. Sometimes I think of giving up entirely, but then I remember how much people here need this shop.

Interviewer: How does this uncertainty affect you?

Respondent: It's hard, my sister. Very hard. Imagine working every day, but you're not sure if the place you're working from will still be standing tomorrow. It's like you're just living on edge. I can't even think of expanding the business because, really, what's the point?

Interviewer: What do you do to cope with all this?

Respondent: Hmm, I just pray. Every morning, I pray to God for protection.

Interviewer: Have you thought about relocating completely?

Respondent: Oh, I want to! I've been thinking about it. I will leave and only manage the other shop.

Interviewer: Has anyone in the community been able to move?

Respondent: A few people have. Those who have some money, or maybe family members abroad, they've managed to leave. But for most of us, we're stuck here. We just keep managing, hoping that one day, things will change.

Interviewer: And what do you think the government can do to help?

Respondent: The government? Hmm. They started building a sea defence some years ago, but it didn't reach here. This new government has not continued the project. I voted for them because I thought they would help us, but now, I regret it. They have not been fair to us. I will not vote for this government again because they have lied to us. They only did it for Glefe, the next community. We are pleading with them to come and finish the project. If they can complete the sea defence, at least we'll have some peace of mind. So, we are pleading with the government to finish building the sea defence early so that we can also work here in peace. The way we are working, and the sea is destroying the work, it is causing us to waste money. Yeah, so we are pleading with them to do the sea defence, they should do the defence for us early.

Interviewer: Do you think they'll listen to your please?

Respondent: I don't know. Sometimes it feels like they've forgotten us. But we keep talking, we keep pleading. What else can we do? But the truth is, we need help. Without help, this shop and everything else here might not survive another year. So, we are pleading with the government to finish building the sea defence early so that we can also work here in peace. The way we are working, and the sea is destroying the work, it is causing us to waste money. Yeah, so we are pleading with them to do the sea defence, they should do the defence for us early

Interviewer: Is there anything else you would like to share?

Respondent: No, please.

Interviewer: Thank you so much for opening and sharing your story. It's not easy to talk about these things, but your voice is important, and I'll do my best to make sure it's heard.

Respondent: Thank you too. I hope so too. We're just living on hope now.

CLIMATE MENTAL HEALTH INITIATIVE

TRANSCRIPT

INDIVIDUAL INTERVIEW_11

Date: 20th July, 2024

Transcription code

(…) – Incomplete sentence

(xxx) – Not audible

[overlapping talk begins

] overlapping talk ends

(..) pause

'Dressss' lengthening of a word

Becau – cut off, interruption of a sound

I DON'T – Loud sounds/words

(Left hand on neck) body conduct

Interviewer: First author

Interview starts – Participant 11

Interviewer: So good morning once again.

Respondent: Good afternoon, how are you?

Interviewer: I'm fine thank you. We are conducting research on climate change impacts and mental health in your community. Some questions I will ask you include: How have you experienced climate-related incidents in the past year? How does climate change (sea level rise) currently affect your life, family, or business? How do you cope with the challenges caused by the sea level rise? How do you feel when you think about climate change (sea level rise)?

Respondent: Please have a seat

Interviewer: As I was saying, we are conducting a small study to understand how changes in the weather and water levels are affecting people. Specifically, we want to learn how these changes are impacting daily life, living conditions, and overall well-being. Our goal is to hear directly from those who are experiencing these changes. The interview will take about 30 minutes of your time. Participation is completely voluntary, meaning you can choose not to take part if you wish. However, if you're willing to participate, I'd be very grateful. May I kindly ask for your permission to conduct the interview with you?

Respondent: Oh yeah do the interview, I'm listening.

Interviewer: ok, thank you.

Respondent: alright.

Interviewer: And so first of all I want to know your age, marital status, your kind of faith you belong, the work you do,

Respondent: Alright, alright, I do dreadlocks, I'm not married

Interviewer: ok. What about your age?

Respondent: I'm just here. I'm here. I'm at the beach here.

Interviewer: ok but like your age like how old are you?

Respondent: I'm 29 years.

Interviewer: 29 years?

Respondent: yeah

Interviewer: oh, ok ok. Alright ok now, which faith do you belong to? Maybe some are Christians, some are Muslims and some are traditionalists, so what is your own?

Respondent: Oh, I don't go to church but I believe in God.

Interviewer: Oh, ok ok ok. Please howe long have you stayed in this area?

Respondent: I was born here.

Interviewer: ok you were born here?

Respondent: Yeah, I'm from here.

Interviewer: So, for like how many years if you calculate, how many years?

Respondent: if I calculate it like more than 19 years.

Interviewer: 19 years? Oh, that is a long time. That's a very long time. So, for instance, in your household, are you the one in charge? Are you the family head? When it comes to titles or authority, what is your role?

Respondent: where do I stay?

Interviewer: No, You see, at home, there's usually someone who looks after the family,maybe the head of the household or someone in charge. So, where you are staying right now, who holds that role?

Respondent: I'm the head there.

Interviewer: Okay

Respondent: I'm the head there.

Interviewer: You're the head of the household, like the husband who oversees the home, takes care of things, and looks after the children?.

Respondent: yeah, sake of I'm the man, I take care of the house. yeah

Interviewer: Oh, ok so are you the only male in the house?

Respondent: Yeah, I'm the only male in the house so I take care at home.

Interviewer: like you have a younger sibling?

Respondent: naa only me

Interviewer: so, you don't have siblings?

Respondent: I have siblings, but they are not with me.

Interviewer: ahhhhhhhhhh

Respondent: Everyone is someone else

Interviewer: oh, ok ok ok ok ok so you are the only one staying here?

Respondent: Yeah, I'm the only on staying here.

Interviewer: oh, ok ok ok but do you have a child?

Respondent: I have one child.

Interviewer: You have one child? How is he?

Respondent: my child is like three years.

Interviewer: Three years? Male or female

Respondent: Female.

Interviewer: oh nice, nice please right now when look at the climate change, the weather, the water levels whether its rising or falling, like how have you experienced it over the past 10 years so far?

Respondent: like the water the sea or the weather?

Interviewer: like the sea, the weather…

Respondent: The sea has not been beneficial to us at all because we have been waiting for the sea defence project to be completed for a long time, but it hasn't been done. This has been a major concern for us, as the sea is causing destruction. The water is eroding the beach, and it's worsening over time. If you like, I can show you a video from now and compare it to a video from 2015 to show you how this place used to look. Back then, where the water has now reached was actually the location of our park. Many buildings around here have collapsed. This place has been deteriorating for a long time, not just recently. In the past, the source of the water was at the same level as where the sea defence now stands. That area used to be where our park was, but now it's all been destroyed because they started the sea defence project, got to this point, and then stopped

Interviewer: Oh ok

Respondent: We are pleading with them to complete the sea defence project as soon as possible so we can continue our work without delays. The way the water is destroying our work is causing us to lose money, and it's deeply frustrating. We need them to finish the sea defence quickly to prevent further losses and help us stabilize our livelihoods.

Interviewer: do you see the changes in the climate, like the weather, the rain, and the rainfall, when you compared to the previously like this has the way it rains changed. whether the weather has become hot or cold, so condition like you how do you see it?

Respondent: As you already know, the world has become much hotter now because of the changing weather. Yeah, it's hard to feel happy under these conditions, how can you feel happy when so much is troubling us? Yeah

Interviewer: But have you seen that in respect to this place, this place, ... you will see that it will rain here maybe it rains maybe previous years, or have you seen any changes like it doesn't rain again or still it rains or it rains seriously but the amount you get, that is how you will see the changes that has happened or the way...

Respondent: As it was at first, it is the same thing.

Interviewer: it's the same?

Respondent: Yes

Interviewer: Ok,

Respondent: It's the sea that is disturbing us here. When it comes to rainfall and everything else, we are okay, but the sea is the real problem. You see, we are very close to the sea, and that's what's causing all the trouble.

Interviewer: ok

Respondent: So, it's the sea that is disturbing us, not the road people. It's the sea we have, and that's the reason for all this trouble..

Interviewer: Oh ok.

Respondent: It is the sea that is really disturbing us.

Interviewer: and this thing too, the hotness and the coldness of the environment,

Respondent: Oh

Interviewer: like the way the weather becomes hot, whether like there is heat or normally here is cold, during the nights and things, have you look at it? Has there been changes or that's what you've …

Respondent: The heat is unbearable right now. I stood up and went to the top, but when I came back, I couldn't take it anymore because the sun is too scorching. There's so much heat in the world.

Interviewer: So, when you compared to the previous years and today, how will you rate the heat level?

Respondent: Oh

Interviewer: The time, is it more or last time previously it was more or how do you see it?

Respondent: Oh, the way I see it is different from how everyone else feels, and it's also different from how I'm feeling too. Yeah.

Interviewer: So, I want to know you are feeling...

Respondent: Oh, it is like this for me for now. I'm okay with everything else, but the water is what's disturbing us. That's our problem. With the sun and other things, we don't have any issues. The water is the only thing that really matters to us

Interviewer: And... have you experienced or seen anything regarding how the weather or climate has seriously affected the community members here? What consequences have they faced?

Respondent: Oh, no no no for this place, the weather hasn't affected anyone sake our weather is ok we are ok here

Interviewer: So here like, when the water comes, it doesn't cause flooding or...

Respondent: Oh, when it rains too much, it causes flooding even the roads become full of water, the gutters become full and stop flowing. yeah, the gutter becomes full ...

Interviewer: So, when it happens like that, what do those in this community do?

Respondent: Oh, we do prepare ourselves for now everything is ok. Small small everything will be ok.

Interviewer: so, when they prepare themselves, do they leave the area?

Respondent: No, you see, at first when it rains, the water enters the rooms and other places. When they came to work on the gutter, they demolished all the buildings, saying they would renovate it, but they haven't completed the work. So now, when it rains and the gutter gets full, the water starts flooding into our rooms again. We are pleading with them to come and finish the gutter for us, so that when it rains, we won't have to worry. They've demolished so many buildings for this project, but nothing has been done. When it rains and you're driving, we can't even pass through because the gutter overflows and spills onto the road. The gutter disappears, and if you're in a car, you could fall into it. Yeah.

Interviewer: So right now, when it rains, does the water flow into the sea or your homes? Or does it do something else?

Respondent: Yeah

Interviewer: or it doesn't come to your home?

Respondent: For the sea, there's a specific time when it acts up. It's not just the rain that causes the sea to become destructive. The sea has its own cycles, which is why we need defences. Yeah, because for six months, the sea can be calm, and for the next six months, it can cause destruction. It's a cycle that we have to deal with

Interviewer: I hope you study it?

Respondent: Yeah, this is where we are. Someone has been here for about 25 years. Yeah, and so we continue to live here

Interviewer: hmm ok

Respondent: So, the defence is what is important to us. For the sea, by six months' time, we don't get peace here. When you even come here, you can't sit down.

Interviewer: does it disturb?

Respondent: The water? Oh, right now it’s quiet, but in six months, you'll see. Come back in six months, and you’ll notice that when the water fills up, it covers this whole area

Interviewer: Wow

Respondent: As we sit here, when the water fills up, it covers this whole area. Yeah, this place turns into a sea; all of this becomes part of the sea.

Interviewer: you can see that the water comes here

Respondent: Yeah yeah

Interviewer: but how the climate change affects you?

Respondent: yeah, the water. Do you remember I mentioned that we don't have a problem with weather change? What really matters to us is the water. The defences are important because it's the water that is disturbing us; it's the water that's causing our issues. Like the way it is disturbing us?

Interviewer: hmm.

Respondent: You see, we have wasted a lot of money here. We've spent more than 2 billion every year, and the sea has taken so much from us, yet we're still wasting money. God has chosen you to help us, and we will give thanks for that. Right now, for those of us here, the water is what matters most; it's the water that's really disturbing us. Dansoman Beach wasn't supposed to be like this. When you visit, you used to feel the beauty of it, but now the water has destroyed Dansoman Beach and everything around it. Have you seen the defences they built? If they had properly implemented them, we might have been better off. But instead, the water comes and brings pressure on us. Because we’ve pushed everything to one side, all the pressure has shifted here, which is why the water is disturbing us. We really need those defences.

Interviewer: hmmm

Respondent: Yes, we have really lost a lot. We've lost more than billions; I can say we've spent over 2 billion every year. We've been here for more than 15 years, so if you calculate that, we’ve spent about 30 billion. If we had taken that money to the city, we could have built an entire estate. But we want the seaside to be nice.

Interviewer: so, So, when changes happen like this, how do you adjust? How do you position yourselves?

Respondent: You already know that wherever your money goes, you have to be steady there. If your money is going somewhere and it's being wasted every day, how would you feel about that?

Interviewer: How are you are managing all these it?

Respondent: Oh, we feel bad, but we continue to manage little by little. This morning, we talked about the defences. That's why I asked you to sit down when you arrived; it's the only thing that matters to us. Considering the money, we've wasted here, if the defences had been in place, you would have felt a significant difference when you came here. You would even want to leave quickly because of how it is.

Interviewer: really? When you look at all these things, does it affect your sleep? Does it disturb does it disturb your sleep or when you sleep, you sleep?

Respondent: Lady, do you know that wherever your money goes, you can’t find peace? If you're wasting money on something that's always being destroyed, can you really sleep? You can’t. We're not in a happy mood; we haven’t experienced happiness in a while. Our situation is better now, but the water keeps coming. In *name withheld* , they’ve demolished everything, and now the sea is close to the street. The street is right here, and the sea is right there. We’re defending ourselves little by little. If we hadn’t taken action, the sea would have reached the street a long time ago. We've done concrete work all around this area. Even if the land goes down, at least we have the concrete to support it. You can see that it has eroded all around, but we’re still working on the defences. We are still spending money, and we hope you can help us. God has chosen you for us.

Interviewer: so, when you look at how now things are going, in future when you look at the community, how do you see the future?

Respondent: Oh, I have some faith that in future, this place will be better. We know...

Interviewer: so, your future how is the rate?

Respondent: Oh no that one you want to know the future issue, when it happens you will see it. It hasn’t happened so we can’t talk about it.

Interviewer: but you have hope that.

Respondent: Yeah, I believe in many things. You see, I have 'Believe' tattooed on my hand.

Respondent: Ok let me ask you something, if you lost your hundred dollars how do you feel?

Interviewer: oh, I will feel sad

Respondent: And if you feel this way about a hundred dollars, how would you feel about twenty billion? Just look at how you’re feeling now. Can you even sleep when you think about billions? Even your stomach will feel uneasy because you’ll be worried every day. We have to think about

it because things haven’t been good; we’re wasting money. When we waste money, we don’t see any benefit from it. We should be making a profit, but instead, we’re just putting money into the ground. No profit comes from that. After investing in the ground, the sea just takes it away, and we gain nothing. The only thing that can bring us profit is our defences.

Interviewer: hmmm

Respondent: You see, when you’re committed to something and have faith in it, no one knows what might bring you down. You’ve made a promise to yourselves that you will see it through, whether someone helps you or not. For us, we’ve decided that we are going to do it.

Interviewer: There are people in the community facing various challenges, while others may be better off. Considering all these factors, what do you think the future of this community will look like, especially with the current weather patterns and rising concerns? How do you see the future holding up under climate change and these circumstances?

Respondent: If the community is to improve, it will depend on the condition of the beach. The beach is crucial; it’s what draws people to the community. If we can protect the beach, then those behind us won’t face any issues. So, our main focus is on defending the beach

Interviewer: How has this situation affected businesses in the community? Specifically, how has it impacted you personally and those around you,both the boys and the girls? How has it affected their jobs and overall business here?"

Respondent: Everyone has a different job they do.

Interviewer: ok

Respondent: My job is different from what others do, so I can't speak for how it has affected everyone. I can only discuss what is impacting me. Currently, we are not in a good condition. Those who live in the town are not affected compared to those who live close to the beach.

Interviewer: When the water overflows and floods, is everyone still able to go to work?

Respondent: "No, when the water comes, it doesn’t reach the roadside unless we break down certain barriers. We, the people living by the seaside, are the ones who suffer when the water rises

Interviewer: So, do all the people at the seaside work here?

Respondent: No, we are the only people here. The owner is not here with us.

Interviewer: Oh, I see. So what strategies do you use to cope? How do you encourage yourself not to let things worry you, despite what has happened?

Respondent: Everyone has their own way of solving problems. For us, we haven't finished addressing ours because of various issues. Our problems haven't gone away. We'll only be okay when those issues are resolved. But for now, we are managing and in a good condition.

Interviewer: Are you in a better condition?

Respondent: Oh, yes. Last year was destructive, and now that we've entered a new year, it feels like it might bring more destruction. Every year, the sea causes damage. It seems like each year brings new challenges.

Interviewer: What do you do to encourage yourself? It must be tough to lose money and face so many challenges. How do you cope with all the stress and difficulties during these times?

Respondent: I feel connected to God, and that gives me strength. I believe that even though we've faced losses, we will eventually recover. Everything will be alright. Yes, I believe everything will be okay. This place won't always stay the same. Dansoman Beach will return to its old ways.

Interviewer: Apart from that, what are you and the community doing to address the issues you're facing? How are you managing or coping with these challenges?

Respondent: I mentioned earlier that everyone lives their life differently. Here, we feel isolated; no one else is with us. The townspeople aren’t part of our experience. We’re the only ones feeling these challenges because no one supports us. We wake up every morning to clean this place ourselves. We don’t know what issues others face, so we can only speak for ourselves.

Interviewer: "Since you’re here and no one comes to support you, what do you do to address those problems? Do you feel you don’t need that much help?

Respondent: We continue, we continue we continue to move forward small so we are waiting for maybe our helper. That’s why we have reinforced these areas with concrete. We believe that using concrete will help protect this place until we can complete our efforts. If we don’t take these measures, we risk losing everything, so we need to safeguard ourselves as best as we can

Interviewer: Do doctors, government officials, and other leaders ever come to speak with you about what’s happening here?

Respondent: No oo,

Interviewer: Nobody comes?

Respondent: No, nobody has come here to talk with us like you are doing now. If someone had visited, I wasn’t here, but I haven’t seen anyone come to engage with us in this way before.

Interviewer: When the issues arose, did they provide any guidance on what you should do?

Respondent: WHO? No, nobody cares about us. They wish the whole place would be destroyed so they can sell it to others. We have to think about ourselves because no one else does. Even in this community, nobody considers our needs. We've relied on our own resources to handle things; it's not their money, so they don't think about us at all. The people in the community don't help us. It's the local residents who want this place to be destroyed. They are the ones who don't want to see any development here.

Interviewer: Hmm

Respondent: Yeah.

Interviewer: Oh, I see. So, what do you think the government could do to help? If someone wanted to make a difference here, what actions do you believe would solve the issues we're facing?

Respondent: the defence.

Interviewer: is it only the defence?

Respondent: Yeah, it is only the defence that is important to us. It is only the defence, when they build it…

Interviewer: Everything will be fine?

Respondent: Yes, everything will be fine if they can provide security for us. Once they handle the defence, we can take care of the rest ourselves. Defence is the only thing that's truly important to us.

Interviewer: Do you ever think about your situation here compared to others? How does it feel to live here? Do you sometimes feel that your life is more difficult or unfair? I'm curious about your thoughts on this.

Respondent: I think about how everyone has their own life. If someone doesn't like how I'm living mine, that's their problem, not mine. I'm living for myself, and they can live their own life.

Interviewer: I mean, in this area, it rains a lot, and we spend a lot of money here. Meanwhile, others live in places where the environment is more conducive. Do you sometimes feel deprived or that it's unjust? I'm wondering if you feel that way compared to people living in other areas who don't have these issues.

Respondent: That's true. As I've said, we're really on our own here. Neither the MP nor the assemblyman pays attention to us, and even the chief doesn't seem to care. They don't support

what we’re doing; instead, it feels like everyone wants to see this place destroyed. No, we focus on our own issues. We’re protecting ourselves because we believe that if we take care of our own needs, we’ll be better off.

Interviewer: Is there anything else you would like to share?

Respondent: No, please.

Interviewer: Oh, ok ok I thank you. I thank you that you’ve done the interview with me

Respondent: I also thank you. Alright

CLIMATE MENTAL HEALTH INITIATIVE

TRANSCRIPT

INDIVIDUAL INTERVIEW_12

Date: 1st June, 2024

Transcription code

(…) – Incomplete sentence

(xxx) – Not audible

[overlapping talk begins

] overlapping talk ends

(..)   pause

'Dressss' lengthening of a word

Becau – cut off, interruption of a sound

I DON'T – Loud sounds/words

(Left hand on neck) body conduct

Interviewer: First author

Interview starts – Participant 12

Interviewer: Thank you for agreeing to speak with me today. Can you tell me a bit about how the sea has affected this community over the years?

Respondent: *(sighs)* The water was very far from our homes. Ooh! Very far. I gave birth to all my children in this house and the sea was not close to us like this. The water was very far from our homes when I first moved here. Back then, you couldn't even see the sea from here. It was peaceful. The water was not this close to us. If someone told you the sea would be this close, it would've been a lie. I have lived here for over 40 years and worked hard to build my life in this community. I started with nothing, and over the years, I managed to put up six rooms, one after the other. It wasn't easy, but this was home. This was where my children grew up, where I built my dreams. Now, all that hard work feels like it's being swallowed by the sea. Everything I've sacrificed for is at risk, and I don't know if I can save it. It's heartbreaking.

Interviewer: That must be such a shocking change. How has this affected your daily life?

Respondent: oooooooooooooooooh! Everything has changed. But everything was washed away. The sea came and destroyed everything,my house. All houses on this lane have been destroyed by the sea. Now, we are just holding on, but I don't know for how long.

Interviewer: How are you managing to make ends meet?

Respondent: I have no source of income now. I used to rely on the rent I got from my six rooms, but those rooms have all collapsed because of the sea. Now, I depend on my children to support me. My daughter, who use to live with me before she married, sews dresses. You can see her right in front of the house. She's the one who feeds me and keeps things going, but even she struggles because clients are few, and people in the community don't have much money to pay for clothes. Every day, I worry about the future. I used the little savings I had after the sea destroyed my home to try and fix parts of it, but it's never enough. I feel like I'm stuck, just waiting for the sea to take away what little is left of my life. Where my daughter is sewing right now is just as risky as where we're sitting.

Interviewer: mmmmmmmmmmm

Respondent: The sea can destroy this place at any moment. It's frightening because if the water comes, her business will collapse too. I'm already sleeping in two of the remaining rooms, and she's using one of them for her sewing. That's all we have left,just these three rooms,and even they are barely standing. The walls are weak, the foundation has shifted, and every time I hear the sound of the waves, I pray that the sea will spare us just a little longer. She has managed to set up her sewing machine and tries to make clothes for people in the community, but how can she thrive when the sea could destroy everything? Sometimes, when it rains heavily, the water creeps in. We have to move things around to prevent them from getting soaked.

Interviewer: mmmmmmmmmmm.

Respondent: The stress is too much, and I can see it in her eyes. She doesn't say it out loud, but I know she's worried about what will happen to both of us if the sea finally takes this house. I wish we had the means to move elsewhere, but for now, we are stuck here, just hoping and praying. So, her business is not doing well, and the little she earns is what we use to eat. It's a hard life, my child. Very hard.

Interviewer: mmmmmmmmmmm

Respondent: Hmm, my child, sometimes I sit and think to myself, "Maybe I should have built somewhere else, far away from the sea." But how could I have known? All my life, I have been here. I was born not far from here, and everything I have ever worked for, my whole life, has been in this place. My spirit is tied to this land; I cannot leave.

Interviewer: I see

Respondent: When I was in my prime, I was very energetic and worked so hard. I made sure to build this house with my children in mind, so they could have a place to live and call home. I built six rooms, you know. I was so proud of what I achieved. But now, the sea has taken almost everything. All the buildings are gone,just like that. How is that fair?

Interviewer: It must feel so unfair to see everything you worked for destroyed.

Respondent: It's not fair at all. After all my sacrifices and hard work, to end up like this? Sometimes I feel like I'm being punished for something, but I don't know what I did wrong. And look at the remaining ones,the rooms I'm managing to live in. I can't carry them away, can I? No, I can't. It looks like the sea will take these ones too, and I don't have the strength or resources to rebuild again. All this is too much for me to bear, but what choice do I have?

Interviewer: I can see how much this place means to you, and I understand why it's so hard to leave.

Respondent: Hmm, my dear, it's not just the buildings. It's the memories, the life I've lived here, the laughter, the struggles. How do you leave all of that behind? This is where I raised my children, where I built my life. Leaving would feel like abandoning my soul. But staying… staying feels like waiting for the sea to finish what it started. It's a painful place to be.

Interviewer: mmmmmmmmmmmmmmmmmmmmm

Respondent: When I came to buy this land, this place was very far from the sea. If it was close, I wouldn't have bought the land, and I wouldn't have built here. At that time, the sea was so far away that it didn't even cross my mind as a threat. This was a peaceful place, a good place to settle down, work hard, and raise my children. But now… look at it. The sea has taken over everything. If someone had told me years ago that the sea would come this close, I wouldn't have believed them. It would've sounded like a lie.

Interviewer: It's heartbreaking to think about how much things have changed over the years.

Respondent: Yes, my child. I feel cheated. Cheated by time, by nature, by everything. I worked so hard, using all the money I had to buy this land and build my house. Six rooms. Six good, solid rooms! I didn't just build for myself; I built for my children. I wanted them to have a home they could always come back to, a place to call their own. But now, what's left? Nothing. The sea has destroyed everything.

Interviewer: That must be so painful. After all your hard work, to see it washed away like this.

Respondent: Painful is not even enough to describe it. The sea didn't just take my buildings; it took my dreams, my pride, my peace of mind. Some days, I sit and look at the ruins, and I think, What was it all for?" If I had known this would happen, I would have built somewhere else. I wouldn't have risked everything here.

Interviewer: And yet, you're still here. Why haven't you left?

Respondent: Hmm. It's not easy to just pack up and leave, my child. This place is my home. My spirit is in this land. This is where I built my life, raised my children, and struggled to make ends meet. Even now, I can't bring myself to leave. Where would I go? What would I do? Some people have left,they've moved to live with family members in Accra. But I don't have that option. My daughter and I are managing here with the little we have. But even those rooms are at risk. The sea could destroy them any day now.

Interviewer: It must be hard living with that uncertainty every day.

Respondent: Very hard. Every night, I go to bed afraid. I haven't slept properly for weeks. I'm scared the last room might collapse while my children and I are sleeping. And if that happens, where will we go? What will we do?

Interviewer: I see

Respondent: I am, my child. And the worst part is, I feel powerless. Nobody is helping us with the water issue. We plead with the government to finish the sea defence, to protect us, but nothing is done. So, we're left to fend for ourselves. I've even spent money buying rocks and using sacks filled with sand to try and protect my house. But how much can I do on my own?

Interviewer: That must be so frustrating, feeling like no one is listening.

Respondent: It is. We pray every day that the sea doesn't destroy what's left of our homes. God is the only one helping us now. Sometimes, I wonder if He's the only one who even hears our cries.

Interviewer: Do you ever think about leaving, starting over somewhere else?

Respondent: I think about it all the time. But where will I go? I don't have the money to start over. All the money I had is gone,used up trying to repair what the sea destroyed. My children help me now, but I can't keep relying on them forever. I want to leave, I want to go back to my hometown and start fresh, but how?

Interviewer: It's a tough situation, Ma.

Respondent: It is. Some days, I feel like I'm just waiting for the sea to take everything. But I still pray. I pray to the sea to give me some time to stay here until I can save enough money to leave. I pray that God keeps us safe. And I pray that the government hears us and finishes the sea defence. If they do, maybe,just maybe,things will be okay. But for now, all I can do is wait and hope.

Interviewer: mmmmmmmmmmm

Respondent: My daughter, who sows here, is married. So, in the evenings, she goes back to her husband's house. It's just me here alone at night. Sometimes, it gets so quiet that I can't help but think about everything I've lost, everything that's been taken away by the sea. Her husband is a

good man, but they're just starting their life together. They don't have much either. How can I go and stay with them when they're already struggling to make ends meet? I don't want to be a burden to them. They're trying to build their own future, and I can't add to their worries.

Interviewer: Do you visit them often?

Respondent: I do. I visit them from time to time. They're always happy to see me, and I enjoy spending time with them. But at the end of the day, I always come back here to sleep. This is my home, even if it doesn't feel like it anymore. Sometimes, I wish I could just leave everything behind and stay with them. But my spirit won't let me. It's like a part of me is tied to this place. No matter how bad things get, I can't bring myself to leave completely. Yes, I want to leave. My body is here, but my soul is not here. I am saving money to leave this community.

Interviewer: It must be hard, being here alone at night.

Respondent: It is. I lie awake most nights, listening to the sound of the waves, wondering if the sea will finally take what's left. It's a constant fear. But what can I do? This is all I have. My daughter tries to encourage me, and I know she wants to help. But I also know they're doing all they can just to survive.

Interviewer: mmmmmmmmmmm.

Respondent: It is. But I have no choice. I pray every day that God gives me strength and that He protects my daughter and her husband. I also pray that the sea doesn't destroy what little we have left. Until then, I'll keep managing, even if it's hard.

Interviewer: That uncertainty must be so difficult to live with. What are some of the ways people in this community are trying to protect their homes and businesses?

Respondent: Some of us are forced to spend money to buy rocks to protect our houses. I have put sand in sacks, but the sea just destroys it. I feel like no matter what we do, the sea just keeps coming closer. We cannot do anything about the sea. It is just coming. Who can stop the sea! No one is helping us with the water issue. The government comes here, takes surveys, makes promises, but nothing is done.

Interviewer: Have there been moments when people in the community came together to support each other during these tough times?

Respondent: Some people come here to encourage me. They tell me to stay strong and keep praying. We all try to support one another, but everyone is struggling. There's only so much we can do.

Interviewer: You've mentioned praying. How does your faith play a role in coping with everything that's happening?

Respondent: (smiles faintly) God is the only one helping us now. We pray that the sea doesn't destroy our homes. Nobody can stop the sea, but we hope that God hears our prayers and keeps us safe.

Interviewer: What do you think would help make a difference for this community?

Respondent: If they continue the defence, everything will be okay. We are pleading with the government to finish building the sea defence. It started, but they stopped, and since then, the sea has been destroying everything. We hope that they continue to build the defence for us.

Interviewer: If the sea defence project is completed, do you think it will solve most of the problems here?

Respondent: Yes, it will help a lot. The water won't come so close, and we can rebuild our homes and businesses without fear. Right now, it's like we're living on borrowed time.

Interviewer: You've endured so much. How do you keep going despite everything?

Respondent: (takes a deep breath) I don't know. I just take it one day at a time. But sometimes, I think about everything I've lost, and it's hard. Now everyone knows his loss has taken over his head, his heart is in pain. That's how I feel sometimes.

Interviewer: mmmmmmmmmmm

Respondent: Sometimes, too, I'm just grateful for life. I look around, and I see that some people have lost their lives. And as much as I've lost, I'm still here. My buildings are gone, yes, but I'm still here to talk about it. I can still wake up every day and try to keep going. So, I give thanks to God for life. There's a man here, you know, his building collapsed on him. His legs were broken, and he's still struggling to recover. I can't even imagine going through that. For me, yes, my buildings are gone, but I didn't lose my life, and I didn't lose my health. I'm still here, even though things are hard.

Interviewer: That's a very strong way to look at things.

Respondent: Well, what can I do? Some days, I feel so helpless. But when I think about it, it could have been worse. There's always something to be grateful for, even in the worst situations. Sometimes, I think to myself, "What if I had built somewhere else?" What if a fire or gas explosion destroyed everything? Maybe it wouldn't have been the sea but something else. Maybe I'd be dealing with a different kind of loss. So, in a way, I have to accept that sometimes life is like that. Things happen that are beyond our control. But whether it's the sea, fire, or anything else, we give thanks to God. He's the one who protects us in the end. Even when we don't understand why things happen the way they do, we have to trust that He has a plan for us.

Interviewer: It's admirable that you can find peace in the midst of all of this.

Respondent: It's not easy, but what else can I do? Life keeps moving forward, whether I'm ready for it or not. I just try to take each day as it comes, be grateful for what I have, and pray that tomorrow will be better than today. And even if things don't improve right away, at least I can say that I did my best. That's all I can do now.

Interviewer: What's your biggest hope for the future?

Respondent: My biggest hope for the future is that things will get better for my children, and they'll have opportunities I didn't have. I look at them and wish I could be as strong as I was when I was younger, back in my prime. If I were as strong as I used to be, I would work hard to restore everything I've lost. I would rebuild it all, but now, I'm weak, and I can't do the hard work like I once did. My body isn't as strong anymore, and that's something I have to accept. But I hope my children will be able to do better, and maybe they'll find a way to fix what the sea has taken from us. My hope is that they won't have to struggle like I did.

Interviewer: Thank you for sharing your story with me. Your resilience is truly inspiring, and I hope your voice reaches the people who can help make a difference.

Interviewer: Is there anything else you would like to share?

Respondent: No

Respondent: Thank you. I hope so too. We are holding on, but we need help before it's too late.

CLIMATE MENTAL HEALTH INITIATIVE

TRANSCRIPT

INDIVIDUAL INTERVIEW_13

Date: 15th June, 2024

Transcription code

(…) – Incomplete sentence

(xxx) – Not audible

[overlapping talk begins

] overlapping talk ends

(..) pause

'Dressss' lengthening of a word

Becau – cut off, interruption of a sound

I DON'T – Loud sounds/words

(Left hand on neck) body conduct

Interviewer: Second author

Interview starts – Participant 13

Interviewer: you can speak with us, right?

Respondent: yeah

Interviewer: Oh, then let's get some chairs and sit down. I can even sit here.

Respondent: ok

Interviewer: Oh, I can even sit here. Good afternoon, thank you so much for agreeing to talk to me today. Before we begin, I want to confirm that this conversation is part of a study to understand the challenges facing people in this community due to the rising sea levels. I'd like to hear your

story and experiences, but you're free to stop at any time or skip any questions you don't feel comfortable answering. Is that okay with you?

Respondent: Ok, go ahead. Yes, that's fine.

Interviewer: Great. Let's start with your age and a bit about yourself.

Respondent: I am 46 years old, and I've lived in Shiabu my entire life. I'm a mother of three, and I used to have a small trading business here before everything changed.

Interviewer: Thank you. Could you describe what life in this community used to be like?

Respondent: Ah, this place was a beautiful community. The water was very far from our homes. We used to feel safe. If someone had told me years ago that the sea would be this close, I would've called it a lie. Nobody could imagine it. Back then, the sea was like a distant neighbor, far away.

Interviewer: And now?

Respondent: Now? (shakes head) The sea is right here, almost inside our homes. It's shocking. Sometimes I stand and look at how close the water is and feel like I'm dreaming. It's hard to believe this is happening to us.

Interviewer: How has this affected your daily life?

Respondent: Everything has changed. I used to have a thriving business here. My shop was full of goods. People would come from far to buy from me. But now? Everything was washed away. My shop, my goods, everything. The sea didn't spare me. It destroyed everything. I have used the little money I have left after the sea destroyed my shop, my goods, my everything. I tried to rebuild, but each time the sea comes, it destroys it all over again. I've used most of my savings to buy small items to sell, but with the water coming closer every day, it feels like I'm wasting my money. It's difficult to even buy more stock because I don't know if my shop will still be here tomorrow. I can't make progress. I put in so much effort, but it all feels like it's going to be washed away again. It's not easy. My son wakes up crying sometimes, saying he heard the walls cracking. I try to comfort them, but I can't even comfort myself. How do you explain to a child that their home might not be here tomorrow?

Interviewer: That must've been very difficult.

Respondent: It still is. All the houses on this lane have been destroyed by the sea. My shop was one of them. Now, I sell small things from my veranda, just managing. But it's not the same. Nobody can stop the sea from destroying our homes, but we pray that it doesn't. God is the only one helping us now.

Interviewer: How do you feel about staying here?

Respondent: My body is here, but my soul is not here. I am saving money to leave this community. I don't want to stay here anymore. I feel trapped. I am still here because I don't have the money to leave. If I could, I would leave today. I'm afraid. Every night I lie awake, listening to the sound of the sea. It's loud and so close now. I'm scared that the last room might collapse on my children while they sleep. The walls are weak, and every time the sea gets rough, it shakes the whole house.

Interviewer: Have you thought about where you'd go?

Respondent: I've thought about it, but where would we go? I have family inland. I would move there, but it's expensive to relocate. Even renting a small place is beyond what I can afford right now. So, I'm stuck.

Interviewer: What do you think about the government's efforts to help?

Respondent: (sighs) The government? Hmm. Nobody can stop the sea from destroying our homes, but we pray that it doesn't. God is the only one helping us now. We've begged the government to finish the sea defence, but nothing is happening. It feels like many people are losing hope. Some have already left for other places, trying to find peace. But for those of us still here, we don't have the option to just leave. We're stuck in this situation, hoping that something changes. I want to leave, but I don't have the money to go anywhere else. I just keep praying and trying to survive day by day.

Interviewer: Have they started any work here?

Respondent: They started in another community, Glefe. The sea defence helped them. But here? We're still waiting. It feels like we've been forgotten.

Interviewer: How are you coping with the constant threat of the sea?

Respondent: It's not easy. Some of us try to build barriers with stones and sand, but it's not working. The sea doesn't care about those stones. It still destroys the buildings. And not everyone can afford to buy stones. Only the richer people in the community can do that. The rest of us just pray.

Interviewer: Do you feel the community is coming together to support each other?

Respondent: Hmm, yes and no. People here are helpful, but everyone is struggling in their own way. Some people have already left, those who could afford it. The ones of us left behind, we just survive day by day.

Interviewer: What would you say to the government if you had the chance?

Respondent: I would plead with them to come and finish the sea defence. We are suffering. The sea is taking our homes, our businesses, our lives. They should do something before it's too late. I just want them to know that we need help. The sea is not waiting, and neither are we. People here are suffering, and it's time for the government to step up and make sure we're not forgotten. We can't wait any longer. My children deserve a chance to live in peace. They deserve a chance to live in a place where they don't have to worry about the sea coming to take away everything they know. But they just tell us to wait. They say they will work on it, but years go by, and nothing changes. I have already used all the money I had left to try to survive. I can't keep waiting.

Interviewer: Do you think they will listen?

Respondent: I don't know. I used to believe they would. But now, I'm not sure. All I know is that we can't do this alone. We need help.

Interviewer: What keeps you hopeful?

Respondent: Honestly, I don't know. I just keep moving because I have to. God. I pray every day that the sea doesn't destroy my home while I'm still here. I also think about my children. I want a better life for them. They keep me going. I have to. If I give up, then what? I still have some life in me, and I'll keep fighting, even if it's just for my children's future. I know we can't control the sea, but we can fight for better conditions for ourselves. My children need me to be strong, even though I feel weak inside. Sometimes, I pray for strength. That's all I can do,pray and hope. My oldest wants to be a nurse. She tells me that one day, she'll take care of me the way I've taken care of them. My youngest wants to be a teacher. They have big dreams, and I want to give them the chance to achieve those dreams. That's why I keep fighting, even when I feel like giving up.

Interviewer: Is there anything else you'd like to share?

Respondent: I just want people to know that we are not lazy or just sitting here waiting for help. We've tried everything we can to survive, but this is bigger than us. Nobody can stop the sea from destroying our homes, but we pray that it doesn't. God is the only one helping us now, but we need help from people too.

Interviewer: Thank you so much for sharing your story with me.

Respondent: Thank you for listening. I hope this will bring some change for us.

CLIMATE MENTAL HEALTH INITIATIVE

TRANSCRIPT

INDIVIDUAL INTERVIEW_14

Date: 15th June, 2024

Transcription code

(…) – Incomplete sentence

(xxx) – Not audible

[overlapping talk begins

] overlapping talk ends

(..) pause

'Dressss' lengthening of a word

Becau – cut off, interruption of a sound

I DON'T – Loud sounds/words

(Left hand on neck) body conduct

Interviewer: Second author

Interview starts – Participant 14

Interviewer: you can speak with us, right?

Respondent: yeah

Interviewer: how old are you?

Respondent: 42 years

Interviewer: How are things happening here lately because of the sea compared to 10 years ago?

Respondent: I went through a tough time (muffled cry)

Interviewer: hmm.. It hasn't been easy

Respondent: serious, I went through a tough time

Interviewer: hmm

Respondent: (lip smack) I invested my life.. I put my all in this place

Interviewer: hmm

Respondent: The way I planned that it would be... so, it

Interviewer: hmm

Respondent: I also realized that it... I planned so many things.

Interviewer: hmm

Respondent: I am from *(name withheld)*. This isn't where I was... I travelled... I was abroad... I am a footballer... I was in Russia. I am a footballer; I came down in 2010.

Interviewer: hmm

Respondent: I was supposed to go back, but family. Problems from home… and I wanted to put things in order before I go back. Through that, I had an effect. There was a fight I went to settle, and they injured me...

Interviewer: hmm

Respondent: So, it caused my traveling to be delayed… I told them and they gave me 3 months...

Interviewer: hmm hmm

Respondent: Later on, a house I own, my brother said he is taking it from me... My own house. If I return it will cause a lot of problems.. so, I said no, I will fight for this... So, it raised a lot of issues... It went to court... They took the house from me... They sacked me

Interviewer: They sacked you out of your own house.

Respondent: It is behind this one... But because of... There when I went… When I came back, you know I am a trainer… So, the beach is where I lived before I travelled. When I travelled, when I was there I saw many things. So while I haven't returned I decided to do something for trainers. So… I cleaned this place. The place was full of garbage. There I didn't see any garbage.

Interviewer: hmm

Respondent: So, every day I had to sweep... I realized that no, this place I can do something.

Interviewer: hmm

Respondent: So, there is a house behind me, and I went to speak with them. The place was destroyed... They said they were not concerned if I could do it. I went to see the chiefs. But there was someone I had to see so I went to sit with that person... I paid, and I started. The players too. So we train every morning... People weren't coming but I had to do it cos I hadn't returned, I'm hurt.

Interviewer: hmm

Respondent: The house owner behind me told me that they wanted to sell their house to me.. and I said, if they wanted to sell the house… so they sold it, and the hotel. It was all over here... I bought the house before I built this one, in 2015 (Sniffs Nose). It was far away.

Interviewer: The sea wasn't here?

Respondent: No, it wasn't here, no no. If someone told you the sea would be this close, no no no.. the sea wasn't here. Canoes were here, a lot of canoes, fishermen. So I hoped to establish, and I put all, my resources into making it big. The whole of Ghana, commanders, police, soldiers, everybo.. lockdown I accommodated every service institution. Oh lockdown police, soldiers, law, everybo.. it was big. [hmm] This is their place… The water never reached here, since I opened [hmm] 2011, it's been 13 years, .. no, I have done no wrong (no) I entertain people.

Interviewer: hmm

Respondent: For 2015, and 2012, the project came that they will do a defence.

Interviewer: hmm

Respondent: We all said okay, if there will be a defence, we don't have any problem. Then.. it was Glefe who saw it.. they had problems.

Interviewer: hmm

Respondent: So when they started, they started from this place. So the more they did it, the more it shifted here. So 2016, they reached Royal Hospital, and there was a change of plans. They wanted to divide it but when they got there, there came a new government to continue… We saw the project went down so we went to ask and they said they had completed the first phase so it is left with the second phase which they are yet to complete. [extend the second phase to complete

it] but if they don't do it, the pressure comes here and it is a long distance so it will affect us, but he said he will protect me first because the pressure that it comes with will affect me.

Interviewer: hmm

Respondent: So he came to see what I have done, it wasn't easy. Here, they will come to sit, when they are on break. So 2020, and uh our madam, minister, ***name withheld***, she came here. I was up there on the rooftop when I saw her speaking with the fishermen so, I came down. They wanted to stop her and ask for a harbor, but the plan was not that…

Interviewer: the harbor?

Respondent: Yes, but they changed her mind. Then the former minister of fisheries... Err err err err. He was also in. At that time he was *a* ***political party name withheld*** so during his time, we started. So when we went there, he told us that my Ga people, at this moment the work.. the fishes [are not good] so allow the defence to pass. They said oo no, it is their turn to be given a harbor. Ah! Do we do a harbor in Accra? So he said when it gets to the bank of the Chemu River, they will open.. aa they said no.

Interviewer: hmm

Respondents: So I told the minister that what they are saying, if the sea is destroyed, they will set off their machines and the boats and go o. They won't get any effect. But for me whatever happens, what machine can they give to me? They said oh forget ***the participant name withheld*** is a ***political party name withheld***. And I said are you making this politics? We are ***political party name withheld*** and it's our turn ***name withheld*** wanted to talk and they said they are ***political party name withheld*** members.. ***political party name withheld***.. so this is what you are trying to tell us? So this is what you're trying to tell us? eerhn? You.. you don't have anything here oo.. the canoes you will take them away..

Interviewer: hmm

Respondent: And mummy said ***name withheld*** don't go... Don't go. So ***name withheld*** told them that if they did… this place would destroy

Interviewer: It will all destroy

Respondent: About 2 years, then we started... Then mummy came here. Earlier, they went to call the regional commander and MCE, (***name withheld)*** and others that they should come and demolish... Oh! What is this? Hehe, but the time they will demolish- they said I'm disturbing them.. so they came, with the minister. I said ah! Mummy and she said, ***name withheld*** it's not me oo it's your people. So my people the fishermen**..**

Interviewer: What are they up to?

Respondent: It's no- So today look at it, spoil… every ah.. ah.. It has spoilt everything.

Interviewer: All the roads?

Respondent: it's almost 4 meters before you reach the sea. Today, it's just coming. Everything is destroyed. I have used the little money I have left after the sea destroyed my hub to build this small pub. I have lost everything. My life is very empty, and I regret not traveling abroad in 2007 when I was a footballer. Now, I am just here with nothing, nothing. It's destroyed but we are not doing anything about it. So it caused me a problem, a deep one. When it got to 2002, I had to sell my property to protect it because it's a business place. So I have to protect it. So I had to buy stones, holders, excavators, spring machines, and pullovers. Another problem I had to sell my cars that were used for work. I put them all here, and they are all gone. (shaky voice, sniffed nose) "The sea has destroyed everything, the building collapsed on me, and I almost lost my legs too. Everything is gone. My hotel... And I had planned for it to be big because I travelled oh I was at the beach. This isn't my problem oo, up to now still, they don't get...

Interviewer: So you bought all of it

Respondent: All, I bought them all. A lot is gone. go and ask about my brother over there (pointing to a man seated on a chair in a ruined building),… he used to have a big bar over there...

Interviewer: Oh

Respondent: So right now you need more stones to protect it. It's not easy oo, 'it's not easy'. I've gone through a lot. Luckily, you see me. I don't like material things it's not done anything... Just because I wanted to do it. Erm it's nothing, just because I wanted to do something but if I can't what will I do?

Interviewer: Yes

Respondent: Then I withdraw. That is what has been motivating me. In God's own time when he says he will do it, he will do it.

Interviewer: All your lands have you sold them?

Respondent: I have sold, I have sold.

Interviewer: You didn't reserve any?

Respondent: It was left with one. But even that one, I have given it to someone and they are also not paid. I have changed ownership to them. They haven't given me even one cedi, that is also another problem you know. They haven't given me anything.

Interviewer: Because at least if you had saved something small then you know you have a property somewhere. Oh property!

Respondent: I will leave the same way I came, I won't take anything along. My brother was a millionaire in Ghana, when he died he went with nothing.

Interviewer: he went with nothing.

Respondent: He was in the fridge for 3 years while the family was fighting over his properties. It's been 3 years, Saturday.. customs officer. We buried him this Saturday. After we buried him, What are you fighting over for? The one who owned them is no more and has left them all. You need to find work to do. Future... Future... It's not me, you are doing it for your future. What at all, where will I sleep? How much will I eat?

Interviewer: Hmm

Respondent: But the future, the children you will bring forth is what you pursue. That is what they will say. Our father, what did… That is why we do what... If not no one would work for properties. They are killing people because of their properties.

Interviewer: Yeah, yeah, yeah

Respondent: If you know God, those things don't come your way. He has a wife, he has children. So the family taking away the properties, what will the woman use to.. what... That is what I tell our sisters but they don't agree with me. You too when you are with your husbands... Assuming they treated you the same way. We don't want the truth.

Interviewer: hmm

Respondent: The problem is too much. When the water comes, it pulls the sand away. It pulls it away. So now I have locked it, so when it comes, it hits it like a defence

Interviewer: hmm

Respondent: So this is what is... Here is the fishermen. Where they used to fish (pointing to the sea). If not all this place would have gone.

Interviewer: hmm

Respondent: I protected it.

Interviewer: hmm

Respondent: They called me mad man, what can I do? If I was a madman I wouldn't have.. I wouldn't invest my money.

Interviewer: At least that is your passion. That has been your passion.

Respondent: hmmm

Interviewer: That is the joy that… So for us, deal with mental health. Do you get me?

Respondent: hmmm

Interviewer: Too much sorrow is not good. And for how long are we going to remain sorrowful? Do you have children?

Respondent: yes, I have children.

Interviewer: So they depend on you. They take strength from you and so they need you to stay alive. They need you to be strong. And too much sorrow weakens us as a person. Do you get what I mean? It doesn't allow us to do what we want to do. And we can't wait to a point where the thoughts become too much. We don't want to get to a point where there will be too much thinking to the point where we have to go to the hospital for medication. It shouldn't get to that. When you take it out to a point... You also need to look at the long term, what you are doing can it stop it?

Respondent: (laughs) For that it's God

Interviewer: For how long? So then we need to look beyond just this. So that if anything happens, it doesn't affect us. Then what will we do if the sea is coming? It is very powerful. If it decides to come, the sea defence won't be enough, we can't say it's 100%.

Respondent: No, no, no.

Interviewer: Whatever you are using to protect it, it's still not safe.

Respondent: No, no, no.

Interviewer: That is why we still need to look into the future. We don't need to put everything in here, because if we put everything in here and it will be able to protect it, we don't have a problem. Because you know you know it has protected it for you. But to put everything in here and it still can't protect it but you lose then it becomes a big issue. So you have to consider all of these things. So that there are other places you can develop or other opportunities you can explore that can help you. So that you don't put all your eggs in one basket.

Respondent: No, no, no.

Interviewer: Because that way if they all destroy then you lose it all then your entire life is destroyed.

Respondent: Yeah

Interviewer: Which shouldn't be the case. Because we are fighting against nature. And when you are fighting against nature, the chances of you winning are very small.

Respondent: hmm

Interviewer: To put all our resources here, when we know that we cannot sustain it will certainly affect us. So rather than putting everything, we should be looking at other things as well. So now it takes a little bit. I understand that since 2011 you started this. For 13 good years, you have lived your life here, and it's not easy. So to go and start something somewhere else, it's not going to be easy like that for you.

Respondent: hmm

Interviewer: But you can give it a try like how you started 13 years ago with this one, you can give it a try and start small, and you can tell a better story than this. Because for this one, this is not how it was supposed to be but it is rather unfortunate we are in this.. so for another 13 years, we can say something promising, that will give you more happiness and you feel human again.

Respondent: hmm

Interviewer: Because the sorrow is occupying a larger space. So from today onwards, I will encourage you to let things go. There are things you can't control, alright. Human beings sometimes are like that, so try your best. The best way is to try your best and focus on a brighter future and look at other ways you can do other ways you can support and look at other things in another way. Okay! Little by little, it will be well.

Respondent: hmm

Interviewer: So long as we are alive things can get better and we can regain everything that we have lost. If we believe, we need to take the right steps. We can regain everything.

Respondent: hmmm

Interviewer: So long as you have life and strength, ah! You understand? So don't let this discourage you but rather be fighting and look at other options, alright.

Respondent: hmm

Interviewer: If God willing they come to do the defence then you can recover everything, and you can rebuild everything. For now, you can't continue to put all your money in here. It will rather leave you with more harm. You have to look at other angels as well, let's do it like that and everything will be fine, okay?

Respondent: Alright

Interviewer: So this is our little words of encouragement we are living you with.

Respondent: Thank you so much

Interviewer: Is there anything else you would like to share?

Respondent: No, please.

Interviewer: We also thank you so much. Keep fighting

Respondent: God bless you.

Interviewer: God bless you too. ***Name withheld*** we thank you so much, keep fighting.

Interview ends.

CLIMATE MENTAL HEALTH INITIATIVE

TRANSCRIPT

INDIVIDUAL INTERVIEW_15

Date: 29th June, 2024

Transcription code

(…) – Incomplete sentence

(xxx) – Not audible

[overlapping talk begins

] overlapping talk ends

(..) pause

'Dressss' lengthening of a word

Becau – cut off, interruption of a sound

I DON'T – Loud sounds/words

(Left hand on neck) body conduct

Interviewer: Fifth author

Interview starts – Participant 15

Interviewer: Good afternoon. I really thank you for giving me the opportunity to speak with you. Please do you agree for have to have this conversation?

Respondent: Yes, please I agree.

Interviewer: Please thank you. So please you will talk about your age, your marital status, you have children or the church you attend.

Respondent: I attend Christ Apostolic Church, and I don't live close to the sea. For about a week, I haven't gone to the city, but today, since our area has been without power, I came here for some fresh air. When I arrived, I was surprised at how much this place has changed. It's not the same as it used to be. I remember standing around here before, but now, I don't recognize it. I've been here for a while, and the way the boys are playing and enjoying themselves really stands out to me. This area used to be a place where people came to relax, and I've even brought my children here

in the past. Seeing it today has truly surprised me. The sea is destroying our buildings. All houses on this lane have been destroyed by the sea. Nobody can stop the sea from coming and we can't do anything about it. It's been a long time since I sat here, and I wonder how my children would react if they came back and saw how much things have changed, especially since they are now in school and have grown up

Interviewer: Please how long have you stayed in this town?

Respondent: oh! I came here with my children. Initially, we lived in Dansoman, Korle Gonno. It's been 15 years since we moved here.

Interviewer: But how many are your children?

Respondent: They are four

Interviewer: Are they all up to 18 years?

Respondent: The one who is 16 years is in S.H.S. the ones left have grown up.

Interviewer: Does the water situation, like what's happening now, affect you regularly?

Respondent: Where I live, thank God, the water hasn't affected us. However, those who are here have faced challenges. I have a friend who used to live nearby, and I was surprised to learn that the water had reached her area. She told me it was a money issue that kept her there. The last time I saw her, she mentioned she had moved to Ablekuma, where her brother provided her a place to stay. When I went to the roadside recently, I couldn't see her house anymore. I learned that her house had been demolished, and now if she returns, she wouldn't even recognize the area where she used to live. I hadn't passed by there in over three years, but today, as I was coming here, I noticed that many people's belongings have been destroyed. The beach has receded significantly, and it's clear that the damage has been extensive.

Interviewer: So, how is your friend doing? How is her situation?"

Respondent: When the water situation worsened, my friend had only been renting her house for about three years. She has children and no husband, so not having a stable place to live was very concerning for her. It was painful to see the water rising, and she didn't know what the future held. She realized she needed to plan ahead. She spoke to one of her brothers, who mentioned a building in Ablekuma where she could move. At that time, her house hadn't been cleaned properly, but she decided to pack up and leave with her children. Thankfully, God helped her make that move.

Now, when I pass by, I see that the area has changed significantly. The water used to be far from where we lived, and there were places where boats were kept and children played. Dansoman

beach used to be a well-known spot, and people would come here to enjoy themselves. Seeing everything now is truly surprising and painful; the water has caused a lot of destruction.

Interviewer: And so, it has really affected affected those here.

Respondent. It's not a small issue. Yesterday, I saw a brother who used to sell here; he was a Rasta, and this was his spot. Today, I'm really surprised by how things have changed. This spot used to be a place where people would sit and observe what was happening at sea, but now it's completely different. I'm astonished by the transformation.

Interviewer: Does it bring worries?

Respondent: Oh, of course, when your belongings get destroyed, it brings a lot of worry. Just one day, everything can be gone, and that's a heavy burden to bear. There was a woman, a friend of mine, who lived here. She hadn't sleep for about a week back then. Every night, she lay awake, afraid that the sea would cause more damage. She was scared that the last room might collapse on her children while they slept. She had seen it happen to others, and she couldn't help but think about it. Her house was destroyed by the sea, and her children almost got trapped under the rubble. Thankfully, they managed to escape, but it was a close call. Now, she didn't live here anymore. She moved away, seeking safety, and her family supported her to relocate to a safer place. I didn't blame her; if I had the means, I would have done the same. But I was still there, holding on, hoping things wouldn't get worse. It was hard to sleep knowing that danger was always so close, and I was worried for my children's safety. It was a constant fear that was hard to shake off. They say the sea defences that were built have only been done to a certain level and then stopped. When you're working on something, you need to stay focused. If it's not done properly, it will lead to destruction. If the water overflows this way, it can cause significant damage. Honestly, it can overflow onto the road and disrupt everything. It's a serious concern.

Interviewer: Sometimes, do they consider what might happen in the future? When you live here and the water overflows, it raises concerns. In the future, if it continues like this, could it overflow again? It's a worrying thought.

Respondent: That's very true; water has more power than anything else. Water and fire are strong forces. If water decides to overflow, it can reach anywhere, even where I am by the roadside. I live behind the road, but if the water chooses to overflow, there's nothing I can do to stop it. When you watch TV and see what's happening to others, it's clear that if the water decides to take you away, there's nothing you can say or do about it.

Interviewer: mmmm

Respondent: It has also affected the finances of many who depend on the beach business. During certain periods, the area gets crowded, and everyone brings their things to sell or enjoy. In the past,

you could come here and find something to do. But now, we come and just stand around, wondering if we can still enjoy the beach like before.

Interviewer: So, since this has happened, what do you do? Even though things have been destroyed, some people are still here. How do they cope with the situation?

Respondent: Honestly, before God, when I heard about what happened here and came to look for my friend, I realized I hadn’t been back in a while. It’s been about three days since I’ve been to the city because they turned off our electricity, and it’s really hot here. I’ve stayed for a long time, and my phone is even off. I’m waiting a bit because I hope the lights will come back on soon, but it’s so hot. In our area, the heat is intense, so I’m just sitting here for some fresh air while I see what’s happening.

Interviewer: So, when you look at the people here, what do they do to prevent what has happened?

Respondent: That’s why I said I can’t really say much. I don’t live here. I’ve heard that things have collapsed, but I haven’t had the chance to come and see it until today.

Interviewer: But when you think about it, what have they been able to do?

Respondent: Oh, if you’re a visitor, you need to find a good place to stay. Sometimes, it's not your own house; you might be renting from someone. With the collapsed buildings, you can't rely on the landlord to wait for your lease to end. You need to find somewhere safe to stay because it's a matter of safety. I hope you understand?

Interviewer: And what do you think we can do about what has happened?

Respondent: Oh, it all depends on God and the government to support us in this situation. Without God's help and government assistance, we may struggle to find a solution. If we don’t have the strength, we can’t do anything about what’s happening.

Interviewer: so aside God and the government, what else can we do to help?

Respondent: Oh, all of us our best…

Interviewer: How?

Respondent: How? While some people can support others, not everyone is able to help. For instance, if I'm in need and I approach someone, like saying, 'Madam, I have this issue,' and if they can help, that’s great. Similarly, if I have the means, I can also support others. That’s how we help each other.

Interviewer: Sometimes, do government officials come and talk to you?

Respondent: Honestly, I sell in the city, and regarding the housing issues, I haven't spoken to anyone about it.

Interviewer: Nobody has also come and asked you questions?

Respondent: Questions? No, no

Interviewer: But what can the government do to support the situation?

Respondent: The government needs to see what's happening everywhere because ultimately, everything falls on them. When situations get dire, whether it rains or shines, we hold the government accountable. Do you understand what I'm saying? For instance, when the roads are in bad condition, we ask the government for help. When water issues arise, we also turn to them for assistance. We are pleading with God to help the government so that they can help us find a safe place to go.

Interviewer: So, when you think about people here, like your sister and others in places like Madina, the water issues haven't affected you as badly, right?"

Respondent: Every day, I reflect on how things have changed. When I was younger, the water was far away, and we had coconuts in this area. Now that we've grown, the population has increased, and more houses are being built. As more buildings go up, the water levels rise, and the sand shifts, causing issues. I remember an old man who lived nearby where there used to be coconuts and fresh air. Now, with all the new construction, I worry that in the future, our generation will suffer the consequences. Every day, I see how we take sand from the water to build houses, which could lead to problems down the line. I feel it's important to speak out about these issues because they affect us all.

Interviewer: mmmmmmmmm.

Respondent: Originally, Dansoman beach was a beautiful place filled with coconuts. Before I had children, we would come from Dansoman just to enjoy the beach. Back then, the water wasn’t as close to the shore as it is now. As more people arrived and began building, claiming ownership of the land, it became difficult to address the issues that arose. When problems occur, we often blame the government, even though it's not the government's fault that individuals built their houses in problematic areas. When flooding happens, we criticize the government for the damage caused, but we chose to build there ourselves. In this world, it seems that whenever something goes wrong, the government takes the blame. As our population grows, the situation only gets more complicated. I remember when this area was just Dansoman beach.

Interviewer: Then please I thank you a lot for giving us the opportunity to speak with you. I really thank you so much. Then please we will end here

Respondent: I also thank you.

Interviewer: is there something else you want to add?

Respondent: Thank you.

Interviewer: Please we thank you.

CLIMATE MENTAL HEALTH INITIATIVE

TRANSCRIPT

INDIVIDUAL INTERVIEW_16

Date: 29th June, 2024

Transcription code

(…) – Incomplete sentence

(xxx) – Not audible

[overlapping talk begins

] overlapping talk ends

(..) pause

'Dressss' lengthening of a word

Becau – cut off, interruption of a sound

I DON'T – Loud sounds/words

(Left hand on neck) body conduct

Interviewer: Fifth author

Interview starts – Participant 16

Interviewer: Good afternoon. Thank you for agreeing to speak with me today. Let's start by getting to know you a bit. May I ask how old you are?

Respondent: Good afternoon. Oh, I'm 32 years old.

Interviewer: Ah, 32. That's a good age,plenty of wisdom and experience! And are you originally from this community?

Respondent: Yes, I was born here, grew up here, and now my family is not here.

Interviewer: That's wonderful. Speaking of family, are you married?

Respondent:: Yes, I'm married. My husband and I have four children,two boys and two girls.

Interviewer: That's a beautiful family! How old are your kids?

Respondent: My eldest is 17; she's in senior high school. The second is 14, then the younger two are 9 and 6. They keep us busy, but we thank God.

Respondent: I can imagine! It's not easy raising children. What about your extended family? Are they close by or in another town?

Respondent: Most of them are here in another community far from here. My parents are retired, but they still live here, and my younger siblings are around too. Family is very important to us.

Respondent: That's true, especially in our culture. Family is everything. If I may ask, what role does religion play in your life?

Respondent: Oh, religion is central. I'm a Christian,a Presbyterian. My faith keeps me going, especially in hard times. We pray every morning as a family, and I go to church every Sunday.

Interviewer: That's admirable.

Respondent: Yes, we all worship together. My husband is very active in the men's fellowship, and my eldest is in the youth choir. We make it a point to involve the children in church activities so they grow up with a strong foundation.

Interviewer: That's a good way to instill values. How has your faith helped you through challenges?

Respondent: It has helped me a lot. When things are hard,like when the sea destroyed our home,I turned to prayer. It gave me peace of mind and the strength to start again. I always say, "God will not abandon us."

Interviewer: That's inspiring. Faith really does give us hope, especially during tough times. Thank you so much for sharing this with me.

Interviewer: I see that your beauty salon is very close to the sea. How has the sea affected your business?

Respondent: Hmm… the sea has been a big problem. You might think it's just waves, but over time, the water keeps coming closer and closer. My room used to be there too, but I have rented now, I just work here, if the sea persists in destroying my shop, I will go back to my hometown. At first, the water would just come during high tides, but gradually, it started washing away the land and then the walls. Eventually, my room collapsed completely. It wasn't all at once,it happened gradually. Now I've rented a place further away, but I still work here at my shop. Oh, the sea has really been a problem for me. My salon used to be in a better spot, further from the water, but over time, the sea kept coming closer. Now, it's right next to the salon. Sometimes, the

waves splash water near the building, especially during storms. It’s stressful. I’ve even thought about moving my salon somewhere safer, but where will I get the money for that? This is my only source of income. I will go to my hometown when the sea destroys my shop. The sea keeps coming forward, and it has destroyed a lot. There were a lot of rooms here, but now they’ve all to rent somewhere else. The water came, little by little, until it collapsed everything. It has made life very hard.

Interviewer: How has sea level rise affected business?

Respondent: My business is greatly affected by the sea. I worry that my building will collapse, but I'm not too bothered; I can always return to my hometown if that happens. Customers still come to get their hair done whenever I open the shop. In fact, I have some customers here right now! I don’t have any issues with them coming in. The real problem is the water damaging the buildings on this lane, and mine is the last one standing. I'm just waiting for the water to destroy my shop, but there's nothing I can do about it. It’s not easy. Sometimes, I try to fix small damages myself by using sand to protect the shop. But honestly, it’s like a losing battle. The sea keeps coming.

Interviewer: How do you feel when you think about rising sea levels?

Respondent: I feel worried all the time; it seems like there’s no solution. While I'm working, hearing the waves makes me anxious about whether my shop will still be here tomorrow. I constantly worry that the water will come for my shop or my neighbor’s. It's really scary. Plus, I have the added stress of thinking about how to rebuild my business elsewhere.

Interviewer: How do you cope with the challenges caused by the sea level rise?

Respondent: Hmm… it’s not easy. For now, I just pray. I also save money to travel back to my hometown, but that would mean starting over completely. Honestly, I just take it one day at a time. I rely on my God to stay strong. Praying helps me calm down when I feel overwhelmed. Sometimes, I talk to people in the community about it. We all feel the same, so it helps to share our fears and ideas. But it’s hard. At the end of the day, you’re just hoping that things won’t get worse.

Interviewer:

Respondent: Hmm, my sister, this government has failed us. It’s like they don’t care about us. We’ve been crying out for years, but nothing has been done. The sea keeps eating our land, destroying our homes and shops, and the only thing we hear is promises,nothing concrete.

Interviewer: Can you share some specific promises they made that haven’t been fulfilled?

Respondent: Oh, plenty! They said they would finish the sea defence project. They came, interviewed us, and then disappeared. That was four years ago. Since then, the sea has destroyed

even more houses. They said they would relocate people whose homes were destroyed, but where are they? We're still here, struggling.

Interviewer: mmmmmmmmmmm. What do people in the community say about this?

Respondent: We are all tired. People are angry. Some even say we shouldn't vote anymore because it's like we're wasting our time. But what can we do? If we don't vote, they will say we don't deserve help. It's like we are trapped.

Interviewer: That sounds very tough. Do you think the community has any hope for future help from the government?

Respondent: Hope? Hmm, hope is small. Unless they bring new leaders who really care about us. But even then, We only hope because we don't have another choice. You see, we are living by the sea because we don't have money to move anywhere else. Some people here used to own big houses, but now the sea has destroyed everything. How can you start over with nothing? If the government won't help us rebuild, what can we do?

Interviewer: I see.

Respondent: Oh, we've gone to the assemblyman, to the district office, and even written letters to the MP. Nothing changes. They always say, "We are working on it," or "Funds are not available." It's like they don't see us as human beings.

Interviewer: That's really difficult. Do you think the leaders understand how serious the situation is here?

Respondent: If they understood, they would act. The sea is literally swallowing our homes. Some people have been forced to sleep outside. If they really understood, they wouldn't ignore us.

Interviewer: How does this affect the community's trust in leadership?

Respondent: Trust? My sister, the trust is gone. How can you trust someone who keeps lying to you? They come here, take pictures, write reports, and nothing happens. If they cared, they would show it through action, not words.

Interviewer: If you could speak directly to the leaders, what would you say?

Respondent: I would tell them to come and see how we're suffering. Let them live here for one week, just one week, and see if they can survive. Then they will know how urgent it is to help us.

Interviewer: That's a powerful message. Thank you so much for sharing your story with me. It's clear that the community needs urgent attention.

Respondent: Thank you too. I hope someone will hear this and do something. We are tired of being ignored.

Interviewer: Thank you for your time

CLIMATE MENTAL HEALTH INITIATIVE

TRANSCRIPT

INDIVIDUAL INTERVIEW_17

Date: 15th June, 2024

Transcription code

(…) – Incomplete sentence

(xxx) – Not audible

[overlapping talk begins

] overlapping talk ends

(..) pause

'Dressss' lengthening of a word

Becau – cut off, interruption of a sound

I DON'T – Loud sounds/words

(Left hand on neck) body conduct

Interviewer: Fifth author

Interview starts – Participant 17

Interviewer: Good afternoon, madam. Thank you so much for your time and agreeing to talk to us. Let's start by getting to know you a little better. Can you please tell me your age and where you're from?

Respondent: Good afternoon, I am 47years and I live in Shiabu. This is my house.

Interviewer: Lovely to meet you.

Respondent: Eh, I'm 47 years old. Ei, time flies, ooo! Just yesterday, I was a young girl running around the market, and now here I am.

Interviewer: (laughs) That's life for you. So, tell me a bit about your daily activities. What do you do?

Respondent: Hmm, I'm a fish seller. I smoke fish. I've been doing this for over 20 years. My husband is the fishermen. He brings the fish, and I smoke it. I used to have a very thriving fish business here. Just in front of this house. But now everything is gone as you can see. I had a nice, big house with five rooms. But now, look at me. Only two rooms left of the house, and it's because the sea took everything else**.** Honestly, I feel like my heart is sinking every time I look at what's left. You see, my house was all I had. The sea just came, took away my dreams, and left me with this emptiness. It's painful to even walk around the place and see those rooms that are no longer a home, just a reminder of what's gone. The flooding, nothing can survive it. The sea doesn't care. It's just water, moving where it wants. It's been happening for years, but every year, it gets worse.

Interviewer: Wow, that sounds like a great team effort with you and your husband. But I hear a hint of sadness in your voice, especially when you say things have changed. Can you tell me more about what happened to your business?

Respondent: *Sighs* My dear, everything was going well back in the day. I had a big smoking oven, and my fish was the talk of the town. People from all overused to come to buy. But now, see… The fish we get from the sea isn't as much as it used to be. The sea has really messed up our business.

Interviewer: That's really unfortunate, How do you feel about these changes, and how has it affected your life and your family?

Respondent: Hmm, it's not easy at all. My husband comes home tired from fishing, but the fish are smaller now, and some days, he can't even catch enough. The floods destroy our market stalls. My husband… he used to be the one who brought in the fish. He would go out every morning, come back in the evening with fresh fish for me to smoke. We worked together, side by side, for years. I couldn't have done it without him. But now, it's different. He doesn't go fishing anymore. After the sea destroyed our house, the business started struggling. The customers disappeared, and business dropped. My husband, he tried to keep going, but the work was too hard. The fishing hasn't been the same either. The waters have changed, the fish aren't as plentiful as before. The government also closes the sea a few times. My husband couldn't find any other work after that. There was no income. It was hard for him, and it's been hard for me too. So, he left. He relocated to another place, trying to find some work elsewhere. He only comes back occasionally now.

Interviewer: I see with all these changes, how do you cope with it all?

Respondent: When I wake up, I stand close to the sea and pray to the sea to give me some time to stay here till I get money to leave. Do you know the sea can hear us? It listens to us. Her voice softens with a mixture of hope and sadness I've always believed the sea listens. Maybe that's why I talk to it every day, hoping it'll help me hold on just a little longer.

Interviewer: That's really difficult,

Respondent: Some days, I want to leave, you know? I want to leave Shiabu, move far away from the sea and find peace. I dream of a new life, a place where I can rebuild, where I don't have to wake up to the sound of the sea crashing on my doorstep. But where can I go? The truth is, I don't have the money to leave. Even if I sold what was left, it wouldn't be enough to start over. I'm stuck. My children are not in the position to help me either. So I sit here and hope, hoping for a miracle.

Interviewer: It seems like you've found a deep connection with the sea, despite all the pain it has caused. How does that prayer help you cope with what you're going through?

Respondent: It helps in some way, you know? When I stand there, I feel like I'm not alone, like someone, something is hearing me. The sea, it's so vast, and yet, I feel like it understands my pain. I'm not asking for much, just a little more time to figure things out. I can't leave right now, but if the sea gives me time, I'll find a way. I'm not ready to give up yet.

Interviewer: I see

Respondent: The sea listens to me, and I believe that somehow, things will get better. I just need a little time. When I pray it doesn't destroy my rooms which is left. I still have hope, sometimes, that's all we can hold on to.

Interviewer: I can only imagine how hard this has been for you. Have you been able to seek help the government?

Respondent: *Sighs deeply* I have tried. But honestly, I'm very disappointed with how things have been handled. *Pauses, voice shaking with emotion* You see, the previous government, they built a sea defence for us here in Glefe. It gave us hope, you know? It was meant to protect our homes, our businesses. But then, when this new government came into power, they didn't continue the project.

Interviewer: I see.

Respondent: *Nods vigorously* Yes! It's like they just left us hanging. They promised they would continue the project, but nothing happened. The sea keeps destroying everything, and no one is doing anything about it. The new government, they don't care about us. They don't understand what we're going through. *Her voice rises, frustration clear* I voted for them, you know? I believed their promises, but now, I can't say the same.

Interviewer: Do you feel like the promises made by the government have been broken?

Respondent: Oh, they have definitely lied to us. The promises were sweet, but they haven't delivered. *Shakes head* They come, take our votes, and then disappear. The sea keeps eating up our land, our homes, and nothing changes. I'll tell you one thing: I will not vote for this government again. They have shown us no fairness, no respect. I can't trust them anymore.

Interviewer: How does it feel to be in a community that is suffering while the government seems to ignore the situation?

Respondent: It's painful. I feel like we are invisible to them. We've been here for generations, living by the sea, trying to make a living. But now, the very thing that gave us life is destroying us, and the people we trusted to help… they are nowhere to be found. I look around at my neighbors, all of us struggling, and it feels like no one is listening.

Interviewer: How has the community responded to the situation?

Respondent: *Shakes head* We're all just waiting for something to change. But nothing happens. The government comes, gives us promises, and leaves. The sea doesn't care about promises. It doesn't care about who is in power. It keeps coming, and we keep losing. We've come together as a community, trying to support each other, but we can only do so much. The bigger problems... those need the government's help. We need the sea defence to be finished, and we need support for the people who have lost their livelihoods. The government promised to help us, but now I see that it was all a lie. I can't trust them anymore. I'll never vote for them again.

Interviewer: what do you hope for your community and for yourself?

Respondent: *Pauses,* I hope for change. I hope the government sees how much we are suffering and that they act before it's too late. For now, I just want to leave this place and survive. If the government can't help, then maybe we need to help ourselves.

Interviewer: I hear you. Thank you so much for sharing with us today.

Respondent: Thank you, too. I hope something good comes from all this, so people like me can continue to feed our families. God help us all.

CLIMATE MENTAL HEALTH INITIATIVE

TRANSCRIPT

INDIVIDUAL INTERVIEW_18

Date: 20th February, 2024

Transcription code

(…) – Incomplete sentence

(xxx) – Not audible

[overlapping talk begins

] overlapping talk ends

(..) pause

'Dressss' lengthening of a word

Becau – cut off, interruption of a sound

I DON'T – Loud sounds/words

(Left hand on neck) body conduct

Interviewer: First author

Interview starts – Participant 18

Interviewer: Good morning once again and I would like to interview you concerning your experiences with living with this community. Some questions include: How have you experienced climate-related incidents in the past 10 years? How does climate change (sea level rise) currently affect your life, family, or business? How do you cope with the challenges caused by the sea level rise? How do you feel when you think about climate change (sea level rise)? Please are you willing and available to...

Respondent: Yeah, I'm ready.

Interviewer: Okay, thank you so much. So first, I will ask you what you do for a living, your marital status (if you are married), and your age.

Respondent: I am 35years old. let me say I'm married.

Interviewer: okay

Respondent: Something like that.

Interviewer: Ok

Respondent: I do barber for a living.

Interviewer: oh ok. Please do you have any child?

Respondent: Yeah, I have one kid.

Interviewer: Oh ok

Respondent: A boy.

Interviewer: How old is he?

Respondent: He is now 10 months old.

Interviewer: Oh ok. Ok. Thank you but how long have you lived here?

Respondent: Almost 20 years now.

Interviewer: Oh, ok so you've lived in this community for 20 years?

Respondent: Something like that.

Interviewer: Ok, so with the climate change, I mean concerning how the weather patterns have changed and the level of the sea rise, do you think it's affecting you?

Respondent: Oh, not now. At first, it was affecting us but for now, we are ok with the sea defence we are ok.

Interviewer: ok how? Can you explain?

Respondent: Oh, at first, the sea used to disturb us a lot. At first, the sea used to disturb us a lot but now due to Mahama's project, we have helped us a lot.

Interviewer: Okay, how was it disturbing you first?

Respondent: In August, the sea used to flood this area significantly. However, since the construction of the sea defence here, a lot has changed for the better. It has really helped us and improved the situation. Many people began to think differently, considering relocating because of the sea. But now, some have returned and are working on their own projects despite the risks. While the sea was once a major disturbance, things have improved, and we are now in a better place.

Interviewer: Ok so you’ve noticed a lot of changes.

Respondent: Yeah, I can say we’ve noticed some so many changes.

Interviewer: Can you give examples?

Respondent: On what?

Interviewer: So how it used to be and now how things are personally with you?

Respondent: Oh personally, things have changed because of the government and things. Things have changed actually due to the coronavirus and things, life have changed. Things are not the same now.

Interviewer: Before the sea defence was built, were you often worried about the flooding? How do you feel about it now?

Respondent: The sea defence was a major problem, but we've managed to address that issue. Now, we are facing different challenges economically, psychologically, and mentally. There are many problems we still need to deal with.

Interviewer: mm Psychologically what problems?

Respondent: Oh, it is affecting us a lot because we have no money, so we used to think a lot.

Interviewer: mmm

Respondent: We used to think a lot.

Interviewer: What do you think the people in the community feel about the sea defence? How do they view the changes from how things used to be to how they are now?

Respondent: Initially, I said that the defence was our major problem, but now that has been dealt with, so we no longer have an issue with the sea. When it comes to the weather, that is something natural, it comes naturally. In the past, during December, the harmattan would start early in the month. But now, it has changed. Even in December, you don’t experience the usual harmattan climate. Instead, it starts in January. For instance, this year, the harmattan began in February, but previously, it would have started as early as December. So, I believe this is something natural. It’s not our fault.

Interviewer: Oh, okay. But do you think that, as you’re describing, how in December you didn’t see the harmattan and now you’re experiencing it, do you think it’s having any influence on you personally? On your life? Or are you just okay with it?"

Respondent: Oh, we are okay with that because it's not anyone's fault. It's natural. God does things in His own way. It's not up to us to tell Him what to do. No, it comes naturally, so we have nothing to say about it.

Interviewer: Oh, okay. And do you also think that it's the same for the community, that whether the harmattan comes or not, it doesn't really matter?

Respondent: oh, oh Oh, we're here whether it's rain or shine, we're in. We have nothing to say, and we can't do anything to change it. So, we just have to live by what has been written, that's all.

Interviewer: Oh, okay. So, before the defence was put in place, how were you coping with the sea rise and the flooding?

Respondent: We couldn't cope with the sea. The sea had covered much of the land, so there was no way to manage it. In the past, the sea was disturbing us a lot, it was really affecting us. That's why I mentioned earlier that so many people relocated from this area because of the sea. But now that the sea defence has been built, we're okay.

Interviewer: Mm, was it disturbing their houses?

Respondent: Yes, it has been disturbing us, but what I've realized is that, like those involved in sand winning, there are people who sell the sand. The sand winning has already started. When they were constructing the sea defence, the contractors told us not to allow anyone to fetch the sand, but I can see that they are still doing it, and it is really hurting us and affecting us a lot. For instance, you can see that sometimes around early August, the sea becomes very rough, unlike how it used to be before. Because of the sand winning, they collect sand from the back of the defence, which really affects us as it penetrates through the ground.

Interviewer: Even with the sea defence it still penetrates it?

Respondent: It sometimes happens, but it is not as severe as it was before. The situation is still affecting us significantly because of what they are doing. If you tell them to stop, they might become aggressive or start arguing with you, so we've decided to leave everything as it is. We don't say anything anymore; we just let them do whatever they think they are doing.

Interviewer: Oh ok. Thank you. Thank you so much. But even though there are these challenges, how are you coping with them?

Respondent: We can't do anything about it. I'm focused on my work, so I have nothing to say about what they are doing. I can't speak out or take action, so I'm just trying to manage my own responsibilities.

Interviewer: hmm so how are you also managing with...

Respondent: As in the sea defence?

Interviewer: No, no. So, about all these problems you've mentioned, specifically, them taking the sand away, and…

Respondent: I'm praying that if I find something better to do, I will leave this country. That's my mindset; I feel like I have nothing to do here. My thoughts aren't focused on Ghana. When we see people doing these things, we might tell the police, but when we go to Dansoman Police Station to report, they don't take any action. This country has so many problems, and honestly, I don't know what to think anymore. My mind is not here in Ghana. I'm just saving for a reason. At this point, I should be in school; I should have furthered my education. Life has taken a different turn for me. I was once a footballer, but now I'm doing barbering due to life's challenges. I feel stuck and unable to express my frustrations. I'm not planning to stay here; I'm focused on moving ahead. There's so much corruption. When people are taking the sand, if you ask them to stop, they will say they've bribed the security. The security personnel see what's happening but don't say anything. If you confront them and get hurt, I have a child at home to care for. I can't put myself in danger because of this situation. If the sea defence breaks and it affects me, it won't only impact me; it will affect the entire community. Some people sit back and think, 'It's not my problem,' just because the sand is being taken from a different area. But these actions are affecting us all. I hope you understand what I'm saying.

Interviewer: yeah.

Respondent: Yeah, because if they're fetching it here and it's not over there, those people won't come to help me. They'll just sit there, looking at me as if I should stand up and fight for my rights. But we all need to come together, unite, and fight against them so they won't come back again. So, if you're watching me do whatever you want me to do, and it hurts me, I need to let them do what they want so I can focus on my own business. Oh, my mind isn't in Ghana right now, I swear before man and God. I'm not thinking about it here; I'm just planning to move on. We're all just living our lives, doing whatever we can, so that when the chance comes, we're ready.

Interviewer: mmm but in your own way what what do you think we can do?

Respondent: Hmm, you see, they came to demolish those buildings over there. If you've noticed, they've already torn down some of them.

Interviewer: ok

Respondent: It was along the gutter where they did the demolition. If there are people the government is supposed to be helping, it's those who live in places that aren't conducive for them. You understand? If you've identified that a location is unsuitable, then you should take action to make improvements to the land, which would benefit both the community and you.

You see, they're playing politics with the gutters and the sea defence projects that should be addressing many of our problems. I don't blame the government; I blame the Pambros company. In any country, a large company like Pambros Salt should be doing more for us. They should be contributing significantly, yet they sit back and watch us struggle. If they could at least give us one hospital, that would make a difference. Just one hospital in this community would be a good start because a company like Pambros needs to step up. Initially, we thought the gutters and other issues were the company's responsibility, but eventually, we were told it was the government handling those projects. Now, they've halted the work. Who will pick it up from here? I doubt that if Mahama comes back, he will continue these projects. It's a cycle,each leader seems to start fresh without continuing the work of their predecessor. If Akufo-Addo comes in and does his thing, and then Mahama returns, he won't continue either. Each will have their agenda, while people are desperate for shelter. You've demolished buildings where families have lived for 25 or 30 years,that's all they have. Now those families, some of whom have children, are left with nowhere to go. They have to start everything over, and we all know that starting fresh in Ghana is not easy. All of this is impacting us deeply. For instance, my nephew used to sell belts, but now that his house has been demolished, he's renting and can hardly take care of his four children. This situation can lead to social issues; those kids could end up roaming the streets and potentially becoming involved in crime. We're calling on the government, but we also need to take action ourselves. Honestly, we might need to consider leaving this country for the sake of our future, leaving it for the older generation. We are wasting our time here. I've been around and played with notable figures like John Paintsil and Charles Taylor. I was a footballer, and I'm not doing this for any recognition. I've been in the company of big names and agents, and if I told you about the cars I've been in, you wouldn't believe it. I've played with people like Emmanuel Boateng and Eric, who was captain of the under-17 team a few years back. I've been with several clubs in Kumasi and other places, but here I am, struggling because of the situation in Ghana.

Interviewer: so, in a nutshell, what do you think we should do?

Respondent: I don't think we should take any action. This is how things are; it's a chain reaction we're all caught up in. I'm not the only one facing these problems,many of us are still struggling with the same issues. So, I don't believe anything will change. In Ghana, it feels like we can't do anything. We're just hoping that everyone gets what they want so they can move on because this place isn't conducive for us to live. Here in Ghana, the elderly aren't helping us. I sit here and think too much if you see my face, you might think I'm older than I am because of all the stress. It shouldn't be this way. In developing countries, you wouldn't see people in this situation. I've been around, played with many people, and sat in nice cars like G-Wagons, but all they want is to use you. They just want to exploit you as a player or as someone to follow them around. Recently, during the voting period, a **name was withheld,** and another man was involved in giving delegates 3,500 each. They handed out that amount in just one day! Meanwhile, we are starving, and they expect us to vote for them or do things for them. I won't waste my time on that. I focus on my life, pray, and come to work. If I make 50 or 80 Ghanaian cedis, that's for me and my family, and I manage it. The next day, I'll save 10 or 20 cedis for future needs. I don't go

out unnecessarily. Yesterday, I was in the room with my wife by 7 o'clock. I don't go anywhere. Here in Ghana, I don't know what to expect anymore."

Interviewer: ok please so are there any mental health policies or interventions?

Respondent: You see, those people loitering around who we think are mad. We are actually madder than them before both man and God. Those people we see walking around and acting like they've lost themselves. They aren't truly mad. We, on the other hand, have gone madder than they have because we don't have money or anything to our name. We're thinking and stressing more than they are. You see, I can walk alongside a mad person, but the way I'd be talking to myself or mumbling under my breath. It's worse than what the mad person would even do. They wouldn't do what I'd be doing.

Interviewer: mmm but have they been any support?

Respondent: who who is in to support?

Interviewer: Mentally?

Respondent: It will affect you if you have no money. As a man, you're not truly a man. I'm sorry to put it that way. If you're a boy or a man, you'll understand what I'm saying. You see, if you're sitting there with nothing in your pocket, sister, you'll find yourself talking plenty, just like I'm doing now. I'm sitting here, talking a lot, and sweating. It's true. When you don't have money on you, life becomes very hard. We are really suffering.

Interviewer: so there is nothing like that?

Respondent: There's nothing. Who is there to help you? We're all suffering. You see that man sitting down with his wife? He's supposed to go to work, but nothing better is happening, so he's just sitting there, thinking. If I'm walking and talking to myself, am I not more "mad" than the people we call mad? Boss, if I'm walking and talking anyhow, haven't I gone madder than the man we call mad over there? It's true. We're suffering. Look at me and the work I'm doing. I can work on Sundays and make GHS 38 or 40 (3.8 to 4 million in old currency). But to do that, I have to start working from 4 a.m. till late. I go to the city to work at Kantamanto, and they'll give me just GHS 20 or GHS 15. What can I even do with that? When I wake up every morning, I leave GHS 40 at home. By the end of the day, my child has already spent GHS 25, and that's just for basic needs. You know how small children are, pampers alone is a problem. Pampers isn't bought, Voltic water isn't bought, and even his chop money for the evening hasn't been sorted. Every night before he sleeps, he drinks Vita Milk. Do you see the calculations I'm doing? If my child's expenses are GHS 25, and my wife needs GHS 40 or GHS 50 a day, and I've already left GHS 40 this morning, what about the evening? How much am I spending in a day? Now imagine I go to Kantamanto, and they give me GHS 20. What am I supposed to do with that? Or someone offers me GHS 70 (7 million in old currency) for security work. How am I supposed to manage that when

transport alone from here to Spintex Road is expensive? Does it even make sense? So, I stay here and work on Sundays, where I can make GHS 38 or 40. On Saturdays, I might make GHS 15 or 18 (1.5 to 1.8 million). Am I not better off than the person sitting there doing nothing? Now, I have to save and stay focused. I don't have money, so I can't afford to have side chicks or waste my time. I have to stay calm, focus on my work, and do what I can. If I make enough money, God willing, then I'll travel.

Interviewer: mm When the government decides to step in and do something to help, what would you want them to do?

Respondent: If the government, by the grace of God, ever gives me the platform to speak, it will cause problems because there's so much to address. Honestly, I don't know what the government can do to help. I'm not speaking just for myself but for all of us. Everyone is affected. Even you, as you're speaking to me, may have your own problems you're facing. So many things are troubling us, and we need to work on them. The truth is, I don't know what the government can do to help unless God touches their hearts. If you were to ask me right now what the government should do to help, I genuinely don't know. Before God, I don't have a clear answer because my mind isn't even in Ghana anymore. My mind is abroad. If the government or anyone could take me to the USA or the UK and pay my fees to get there, I'd be safe. Whatever happens after that isn't my concern. I'll figure it out. That's my dream. If they can't help me travel, then at least give me capital to start my own business or establish something for myself. Those are the only two things I want: help me travel abroad or help me start a business. If I can get one of these, I'll handle the rest, including taking care of my family.

Interviewer: But this community here…

Respondent: in this community unless you ask them, everyone has a problem. For me, I just want to fly, just like that. That's my issue. I can't speak for others and their problems, but from my perspective, my thoughts, and my experience, I can say that I want to travel or find something I can do as a small business here to earn a little for myself. Because honestly, I don't know what else to say.

Interviewer: Yes, please. Then I really thank you

Respondent: oh, I also thank you.

Interviewer: Your time and …

Respondent: Oh, forget! I'm not worried about time; I always have time. If you can come every day until it reaches the government, I would be happy. I really need to meet Akufo-Addo in person, face-to-face, because I truly need to talk to him directly."

Interviewer: okay

Respondent: I also thank you a lot.

Interviewer: yes, please

Respondent: You should also force and do that work for us. If it can get to them, we will really like it. The leaders.

Interviewer: We are praying.

Respondent: They gave each person 35 million, along with a flat-screen TV,32 inches, and shared fridges too. And look at all the money they're spending just for something as simple as voting. How does this benefit the nation? A few people are enjoying, but then they talk about "sharing the national cake equally." If that's the case, why aren't we all benefiting? If something is meant to help the entire nation, everyone should get a share. But instead, some people are receiving 35 million, while people like me don't even see Ghc5. What are they trying to prove? The money always seems to favor one side, like those connected to NPP or big organizations like Zoomlion. Look at the woman who hid such a huge amount of money in her room, where did that money come from? Meanwhile, I just need a little to survive, and I can't even get that. Someone is talking about moving to London with 1.2 billion, yet I can't even get a fraction of that. In Ghana, you can work for 20 years and still not see that kind of money because it never trickles down to the people who really need it. As I'm sitting here, my father's job has been gone for over two years. I've taken on the responsibility of catering for my entire family. I'm not the only one struggling. I have a sister in secondary school, another at Tippers Academy,one of the biggest schools here in Glefe and I'm the one helping to pay for their education. As the eldest, I'm responsible for my younger siblings, my wife, and my child. Yesterday, our electricity meter got spoilt. When I went to ask for the price to replace it, it was 1.1 million (10 cedis). Even something as small as that feels like a heavy burden. So, with everything I'm doing, helping my siblings and running my household, where will the money to sustain us come from? Do you understand what I'm saying?

Interviewer: ok

Respondent: All of this is my debt, so what else am I supposed to do? We're all facing problems,everyone has their struggles. Just yesterday, I lost almost 200 cedis on my phone. The person who did the edges took 80 cedis, and the one who did the meter also took 80 cedis. So, how much is left from the two million? I have to stake bet to survive.

Interviewer: so, as I said earlier, I thank you a lot

Respondent: I also thank you.

Interviewer: yes please, so please I'm ending it

Respondent: if you don't leave it here, we will say things.

CLIMATE MENTAL HEALTH INITIATIVE

TRANSCRIPT

INDIVIDUAL INTERVIEW_19

Date: 20th July, 2024

Transcription code

(…) – Incomplete sentence

(xxx) – Not audible

[overlapping talk begins

] overlapping talk ends

(..) pause

'Dressss' lengthening of a word

Becau – cut off, interruption of a sound

I DON'T – Loud sounds/words

(Left hand on neck) body conduct

Interviewer: Fifth author

Interview starts – Participant 19

Interviewer: We have to ask him first before we can record.

Respondent: Yes

Interviewer: How old are you?

Respondent: 50 years

Interviewer: Thank you for taking the time to speak with me today. We're conducting a study to better understand the experiences of people living by the sea.

Respondent: Have you noticed how the defence efforts were effective? When you go to the seashore, you can see that many buildings have collapsed. The Glefe people have more peace now than the Shiabu people. We want the government to help us complete the defence so that we, the

Shiabu people, can also find peace here. The water is currently causing destruction, and the price of cement has gone up. It's frustrating because someone can buy cement to build a house, but the water will just ruin everything. We are suffering here, and we need help to finish the defence so we can have peace of mind. All the worry is about the sea defence. They are not continuing. That is all our problems. If they continue the defence, everything will be ok

Interviewer: Hmm, regarding the work you're doing, like when you go to the sea to fish, have you noticed any changes in the water?

Respondent: You see, there hasn't been any change in the water. Since we started working on the sea, we've gotten new nets, and we also have a type of fishing light called 'nkrante.' Right now, that's what we're using. We don't have any problems with the sea; we catch enough to eat every day. Some people say that the light destroys the sea, but those who benefit from it don't complain. They go out every day and catch enough to eat. So, please investigate for us to see how you can help; we would really appreciate it."

Interviewer: Do you see that the water, which is causing destruction here, has displaced people from their jobs?

Respondent: Yeah, that's true

Interviewer: Which kind of work do you have here that has caused the rain.

Respondent: There are toilets and showers here; we have facilities set up. However, the water has destroyed everything. The man sitting there has a mother-in-law who owns a spot, but now the water has ruined it all, and we are suffering because of it. All the food we could have eaten has gone to waste.

Interviewer: Has the suffering you're experiencing brought worries and problems to you?

Respondent: Yeah, it is true.

Interviewer: Really?

Respondent: Yeah

Interviewer: Is there anything here that has caused sickness or health issues?

Respondent: Oh, I know many people here who are suffering. Some walk along the seashore at night, collecting things to sleep on. The water has displaced them, leaving some without a place to sleep. We are seriously suffering here. If you could help us with some protection, we would really appreciate it.

Interviewer: Are you saying that you want to request the government to continue the defence efforts?

Respondent: Yeah

Interviewer: But when the defence comes, wont it affect those of you who to on the sea? Wouldn't it affect you?

Respondent: We want to address the small problems affecting us. If the boats here can be repaired, it would allow people to pass through, and we would be very happy.

Interviewer: Oh, ok so they should rather do the boat and create a way so that the sea people can pass through?

Respondent: Yes, we would be very happy. You see, we've also built a small harbor in Accra, which can help us as well. If the defence efforts come here, it will provide us with some freedom. So, we really want the defence to come.

Interviewer: Has the way the water is disturbing the area caused people to leave?

Respondent: Yes, that's true. Many people have left this area because they don't have a place to sleep. Some have gone to the bush to start families there, just to find a place to sleep.

Interviewer: What is your future in this community?

Respondent: Oh, if I can say that our future is, I can't really tell

Interviewer: Your future, how is it like?

Respondent: I can't really predict our future because of the suffering we're experiencing here. We're seriously struggling. Just look at the state of the toilets and the shore; we create a lot of rubbish. If we had proper toilets and the defence came to help improve our situation, we would be grateful. Every time we go to sleep, we would pray for you and honor you for what you've done for us. Please help us so that this place can improve.

Interviewer: emm when you go for fishing, the quantity of fish that you get, has it now increased or decreased?

Respondent: Oh, it has reduced.

Interviewer: It has reduced right? What do you think might be the cause of the reduction?

Respondent: You see, the government imposed a ban on fishing to allow more fish to come back. But now, when you go to Takoradi or Cape Coast, they are using lights to catch all the fish, so the fish aren't coming back. Even with the ban, the fish still aren't returning

Interviewer: How long have you been here, considering how it used to rain?

Respondent: oh, I was born here so I've been here for like 20 years

Interviewer: For like 20 years?

Respondent: Yeah

Interviewer: So, in the 20 years you've been here, has the rainfall pattern changed, or is it the same as it used to be?

Respondent: Oh, it has changed small.

Interviewer: How?

Respondent: It has changed. You see, each season used to have its own rainfall pattern. Sometimes, we go through a season where it hardly rains at all. Each season brings its own unique weather.

Respondent: The pressure is really affecting us and destroying our things. If you want to help, you need to be proactive so that we can improve our situation. Last time, water damaged our belongings, and people came promising support for sea defence, but we haven't seen any action from them. They appeared on UTV and said they would return, but we've been waiting and nothing has happened. Those who suffered damage from the water can't even respond properly when you ask them questions; they might even insult you.

Interviewer: yeah, it's true.

Respondent: Or you don't understand what I'm saying?

Interviewer: Oh yeah that's true.

Respondent: They might insult you because when you ask them questions or try to explain things, they don't follow through on their promises. All the older people here are very angry about the situation.

Interviewer: Chairman, we thank you.

CLIMATE MENTAL HEALTH INITIATIVE

TRANSCRIPT

INDIVIDUAL INTERVIEW_20

Date: 20th July, 2024

Transcription code

(…) – Incomplete sentence

(xxx) – Not audible

[overlapping talk begins

] overlapping talk ends

(..) pause

‘Dressss’ lengthening of a word

Becau – cut off, interruption of a sound

I DON’T – Loud sounds/words

(Left hand on neck) body conduct

Interviewer: Fifth author

Interview starts – Participant 20

Interviewer: Thank you for taking the time to speak with me today. We’re conducting a study to better understand the experiences of people living by the sea. Some questions include how the sea affects your daily life and how you cope with the challenges. The interview will take about 30 minutes. Do you have any questions before we begin?

Respondent: No

Interviewer:

Respondent: We have noticed that the sea level has risen and changed significantly over the past few months

Interviewer: oh ok. Continue

Respondent: Yeah, because because Normally, we experience sea level rise during September and October, but it has now begun to occur as early as February and March

Interviewer: Continue

Respondent: Yeah.

Interviewer: What will you say about the sea defence? What would you say? Do you want the sea defence to continue? Do you want them to continue?

Respondent: Oh yeah. I want them to continue. We will use this area as a harbour because we are currently facing difficulties here. (pointing to the rubbish dump)

Interviewer: Why is there a lot of rubbish at the seashore? Is the sea responsible for bringing in the rubbish?

Respondent: Oh no no no

Interviewer: Who is dumping it?

Respondent: Our wives, our ladies, Our women, they came here at dawn to do that. I used to go out at night, and I would see a woman dumping rubbish. When you talk to her...

Interviewer: Have the things that the sea is destroying affected people's jobs?

Respondent: Actually, the landlords and landladies are now becoming tenants themselves.

Interviewer: Landlords are becoming tenants?

Respondent: I was here. He was also here (points at a neighbour)

Interviewer: oh, he also has to go and rent?

Respondent: Yeah. A lot of people, a lot of people a lot, a lot.

Interviewer: Do you think what is happening here affects people mentally?

Respondent: I believe it has made one person very sick.

Interviewer: The man has become a sick?

Respondent: Because yeah because …

Interviewer: He lost all his properties?

Respondent: Yes, he lost everything, even his speaker; it was taken away.

Interviewer: I see. So, wow!

Respondent: We are suffering. As I am going to sleep, I am afraid that I will wake up tomorrow and the sea has taken my shop. I am always thinking. When I sleep, I can't sleep. Thinking and thinking. Bossu, I beg come back another day ok. Come back later. I have to leave. Come back later seriously.

Interviewer: hmm thank you very much sir. Thank you. I, please how old are you?

Respondent: I'm 50 years. But I was born here. I have lived here for 33 years

Interviewer: oh, so you were born here and you've lived here for 33 years?

Respondent: Yeah, oh I've been going to my hometown.

Interviewer: So, you have 33 years' experience here?

Respondent: Yeah. Initially, there weren't many buildings here, so when it rained, we didn't experience any flooding. But now, there are many roads in this area, and when it rains, it becomes a problem. Before, we used to approach the chiefs and offer voluntary services to raise money, so they wouldn't be bothered when they saw someone building a house on the road.

Interviewer: Alright thank you very much. We are very grateful. Thank you so much.
