# Supplementary material for: Climate change impacts and mental health in poor urban coastal communities in Ghana
Source: PLOS Ment Health. 2025 Apr 8;2(4):e0000284. doi: 10.1371/journal.pmen.0000284 (PMC12798396; doi:10.1371/journal.pmen.0000284)
Supplement: S1 Table — (DOCX) [file pmen.0000284.s002.docx]

**S1 Table: Demographic Characteristics of Participants**

| **Category** | **Subcategory** | **Community** | | |
| --- | --- | --- | --- | --- |
|  |  | **Shiabu** | **Glefe** | **Gbegbeyise** |
| Gender | Female | 26 | 6 | 3 |
|  | Male | 14 | 4 | 4 |
|  | 20-30 years | 9 | 2 | 0 |
| Age Group | 31-40 years | 18 | 7 | 6 |
|  | 41-50 years | 10 | 1 | 1 |
|  | 51-60 years | 3 | 0 | 0 |
| Total Participants | | 40 | 10 | 7 |
