# Supplementary material for: Climate change impacts and mental health in poor urban coastal communities in Ghana
Source: PLOS Ment Health. 2025 Apr 8;2(4):e0000284. doi: 10.1371/journal.pmen.0000284 (PMC12798396; doi:10.1371/journal.pmen.0000284)
Supplement: S2 Table — (DOCX) [file pmen.0000284.s003.docx]

**S2 Table: Thematic Analysis of Collected Data**

| **Codes** | **Basic Theme** | **Organising Theme** | **Global Theme** |
| --- | --- | --- | --- |
| *The water was very far from our homes...*  …*the water was not this close to us.*  *…If someone told you the sea would be this close, it would’ve been a lie”* | *Shock and disbelief about rising sea level* | Experiences of Climate Change Impacts (Sea level rise) | Experience of mental health challenges and coping due to climate change impacts (sea level rise) |
| *I used to have a thriving business here, but everything was washed away…*  *All houses on this lane have been destroyed by the sea…* | *Loss of Livelihoods and Properties* |  |  |
| *…Now everyone knows his loss has taken over his head, his heart is in pain…*  *… I almost lost my legs too* | *Pain and despair* | Climate-related mental health challenges due to sea level rise |  |
| *…my fish business has also collapsed.* *I have little money…*  *I have used the little money I have left after the sea destroyed …*  *…no one is helping us with the water issue.*  *…but nothing is done.* | *Financial strain*  *Learned helplessness and hopelessness* |  |  |
| *I haven't slept for about a week…*  *…I am afraid…*  *…I'm scared that the last room might collapse on my children while they sleep.* | *Feelings of Anxiety*  *Fear of expanding businesses* |  |  |
| *… I don’t know if the water will destroy my business tomorrow.*  *…she doesn’t know when the sea will destroy the shop* |  |  |  |
| *…moved to live with their family members in Accra.*  *They are no longer here because their family members supported them with accommodation”*  *Some people come here to encourage me…* | *Social support* | Participants coping resources (psychological) of sea level rise |  |
| *…* *I will go back to my hometown*  *…I will move there and operate our business there.*  *My body is here but my soul is not here. I am saving money to leave this community…* | *Relocation* |  |  |
| *…pray to the sea to give me some time to stay here till I get money to leave…*  *…but we pray that the sea doesn’t destroy our homes. God is the only one helping us now.* | *Spirituality* |  |  |
| *If they continue the defence , everything will be ok.*  *We are pleading with the government to finish building sea Defence …*  *We hope that they continue to build the Defence for us…*  *…I am forced to spend money to buy rocks to protect my house and hub…*  *…we use the sacks and put sand in them to protect our houses…* | *Techno-Managerial Interventions (Sea Defence )*  *Sand and rock protection* | Participants coping resources (physical) of sea level rise |  |
